# Combined use of Oxford Nanopore and Illumina sequencing yields insights into soybean structural variation biology

**DOI:** 10.1186/s12915-022-01255-w

**Published:** 2022-02-23

**Authors:** Marc-André Lemay, Jonas A. Sibbesen, Davoud Torkamaneh, Jérémie Hamel, Roger C. Levesque, François Belzile

**Affiliations:** 1 Département de phytologie, Université Laval, Quebec, Canada; 2 Institut de biologie intégrative et des systèmes, Université Laval, Quebec, Canada; 3 UC Santa Cruz Genomics Institute, Santa Cruz, CA USA; 4 Département de microbiologie-infectiologie et d’immunologie, Université Laval, Quebec, Canada

**Keywords:** Structural variation, Soybean genomics, Oxford Nanopore sequencing, Transposable elements, Population studies, Crop genomics, Structural variant genotyping

## Abstract

**Background:**

Structural variants (SVs), including deletions, insertions, duplications, and inversions, are relatively long genomic variations implicated in a diverse range of processes from human disease to ecology and evolution. Given their complex signatures, tendency to occur in repeated regions, and large size, discovering SVs based on short reads is challenging compared to single-nucleotide variants. The increasing availability of long-read technologies has greatly facilitated SV discovery; however, these technologies remain too costly to apply routinely to population-level studies. Here, we combined short-read and long-read sequencing technologies to provide a comprehensive population-scale assessment of structural variation in a panel of Canadian soybean cultivars.

**Results:**

We used Oxford Nanopore long-read sequencing data (~12× mean coverage) for 17 samples to both benchmark SV calls made from Illumina short-read data and predict SVs that were subsequently genotyped in a population of 102 samples using Illumina data. Benchmarking results show that variants discovered using Oxford Nanopore can be accurately genotyped from the Illumina data. We first use the genotyped deletions and insertions for population genetics analyses and show that results are comparable to those based on single-nucleotide variants. We observe that the population frequency and distribution within the genome of deletions and insertions are constrained by the location of genes. Gene Ontology and PFAM domain enrichment analyses also confirm previous reports that genes harboring high-frequency deletions and insertions are enriched for functions in defense response. Finally, we discover polymorphic transposable elements from the deletions and insertions and report evidence of the recent activity of a Stowaway MITE.

**Conclusions:**

We show that structural variants discovered using Oxford Nanopore data can be genotyped with high accuracy from Illumina data. Our results demonstrate that long-read and short-read sequencing technologies can be efficiently combined to enhance SV analysis in large populations, providing a reusable framework for their study in a wider range of samples and non-model species.

**Supplementary Information:**

The online version contains supplementary material available at 10.1186/s12915-022-01255-w.

## Background

Structural variants (SVs), commonly defined as genomic variations involving at least 50 nucleotides, are a key source of sequence and functional variation in eukaryotes [1–4]. Indeed, SVs such as deletions, insertions, duplications, and inversions account for more variation in sequence content than single-nucleotide variants (SNVs) in several species (e.g., [5–7]). In addition to their implication in human health [8], SVs play a role in key phenotypes in crops such as soybean (*Glycine max*) [9], maize (*Zea mays*) [10, 11], tomato (*Solanum lycopersicum*) [12], wheat (*Triticum aestivum*) [13], and rapeseed (*Brassica napus*) [14]. Moreover, there is now clear evidence for the significant role played by SVs on ecological and evolutionary processes in various non-model species [15].

Despite their undeniable functional importance, genome-wide population-scale assessments of SVs have lagged behind compared to SNVs due to the lack of power of short reads for SV discovery [2]. Tools that discover SVs from short reads typically rely on one or several types of evidence, either in the form of split reads (SV breakpoint found within an individual read), discordant read pairs (unusual orientation or distance between reads of a pair), or read depth (abnormally high or low coverage at a given position) [16]. Other methods rely on local or genome-wide de novo assembly to discover SV breakpoints at base-pair resolution. These methods can generally detect a larger number of SVs, but they tend to struggle with SVs located in repeated regions and on shallowly sequenced samples [17]. Unfortunately, benchmarks of tools that discover SVs from short reads consistently document sub-optimal sensitivity and precision, issues that can only be partly relieved by combining results obtained with different tools [18–20].

The increased availability of long-read sequencing technologies such as Oxford Nanopore and PacBio in recent years has benefited the study of SVs [21]. Indeed, their increased read length allows them to both cover the span of larger variants, such as long insertions and inversions, and to map more confidently in the low-complexity regions where SVs tend to occur [2]. Several mapping-based methods for SV discovery from long reads have already been developed (e.g., [22–25]) and benchmarked [26], typically performing better than methods using short reads. These approaches have recently been applied to provide genome-wide assessments of SVs in crops such as tomato [12], rice (*Oryza sativa*) [27], and rapeseed [28].

Despite the greater power of long reads for SV discovery, their high cost and typically higher basecalling error rates make them unlikely to replace short-read technologies in the short term. In the meantime, methods that allow short-read data to use the insights gained from long reads are much needed in order to scale the study of SVs from the small cohorts sequenced with long-read technologies up to entire populations. In particular, using short-read data to genotype SVs discovered from long reads shows great promise to allow scaling up the insights gained from long reads. Indeed, any SV whose sequence is sufficiently well resolved could be genotyped from short reads as long as these can accurately map to the SV breakpoints. Although methods for genotyping SVs from short reads do exist (e.g., [29–31]) and have been applied to SVs discovered from long-read sequencing data (e.g., [32]), these approaches have yet to be widely adopted in plant genomics and best practices for their application in highly repetitive genomes such as that of soybean and other non-model species are still needed.

Previous studies have addressed the question of soybean structural variation using either comparative genomic hybridization [33, 34], short-read sequencing [6, 35], or pangenome approaches [32, 36, 37]. These studies have notably found evidence for an enrichment of SVs in genes related to defense response [33, 34, 37] and a role of SVs in determining traits such as seed coat pigmentation and iron uptake [32]. The use of a combined analysis of short and long reads could nevertheless provide new insights into soybean SV biology by allowing the study of sequence-resolved insertions efficiently and at a larger scale. Studies of transposable element (TE) polymorphisms in soybean, for example, have been limited to the identification of TE insertion boundaries [38], but long reads allow for the identification of full-length TE insertions [39].

In this study, we use an approach that combines short-read and long-read sequencing to improve discovery and genotyping of SVs in a soybean population. We first evaluate the overall performance of predicting and genotyping SVs from short reads in soybean and identify best practices for doing so. We next quantify the sensitivity and precision of genotyping Oxford Nanopore-discovered SVs using Illumina sequencing data. Finally, we combine short-read and long-read approaches to generate a comprehensive set of SVs from a panel of Canadian soybean varieties and apply this dataset to analyze population structure, relate SV location and frequency to potential impacts on gene function, and gain insights into soybean TE biology.

## Results

### Benchmarking of Illumina-discovered variants

Our first objective was to assess the performance of SV discovery and genotyping in soybean based solely on short-read sequencing data. To do this, we merged SVs discovered using four different tools (de novo assembly + AsmVar, Manta, smoove, and SvABA) to create a set of candidate SVs and genotyped them in 102 samples using Paragraph. Note that we used different tools for SV *discovery* (also referred to as SV *calling*) and SV *genotyping*; we direct readers to the “Methods” section and to a graphical summary of the methods applied in this paper (Additional file 1: Figure S1) [6, 24, 29–31, 40–54] to get an overview of the pipeline. The total counts of filtered SV calls based on Illumina data per discovery tool, SV type, and SV size class are summarized in Table 1. Genotypes called by Paragraph for 17 of the 102 samples were compared against a set of SVs called from Oxford Nanopore data with a mean average depth of 12X using Sniffles and processed through a SV refinement pipeline (Additional file 1: Table S1). The Oxford Nanopore SVs were used as a truth set due to the long reads’ superior power at SV discovery compared to short reads. Comparison between Paragraph genotype calls and this ground truth was performed by using the sveval R package to compute genotyping sensitivity and precision for various SV types and size classes. Sensitivity was defined as the fraction of SVs in the ground truth set (SVs called by Sniffles from Oxford Nanopore data) genotyped as the alternative allele (non-reference) from the Illumina data. Precision was defined as the fraction of SVs genotyped as the alternative allele from the Illumina data that were also observed in the truth set. We only considered homozygous genotype calls for these benchmarks since we are analyzing inbred lines (see Additional file 1: Figure S2 for heterozygosity rates of the population).

**Table 1:** Number of SVs called from Illumina data per calling tool, SV type, and size class

	[50 bp–100 bp[				[100 bp–1 kb[				[1 kb–10 kb[				≥ 10 kb^a^		
Calling program	DEL^b^	INS^c^	DUP^d^	INV^e^	DEL	INS	DUP	INV	DEL	INS	DUP	INV	DEL	DUP	INV
asmvar	11,018	3575	0	0	14,877	2243	0	0	5748	1	0	0	4681	0	0
manta	9664	3358	453	0	12,378	1815	3114	0	11,463	0	4448	0	7325	5034	0
smoove	4168	0	22	45	6489	0	1208	149	4687	0	981	33	1794	975	47
svaba	7288	2284	673	21	6907	215	16,081	292	2969	0	1548	190	512	458	223
merged^f^	17,199	5023	656	61	22,980	3165	9810	296	13007	1	4316	135	10,640	4696	178

^a^ Insertions ≥ 10 kb are not shown because none were called

^b^ DEL: deletions

^c^ INS: insertions

^d^ DUP: duplications

^e^ INV: inversions

^f^ merged: the dataset merged using SVmerge

**Fig. 1:**
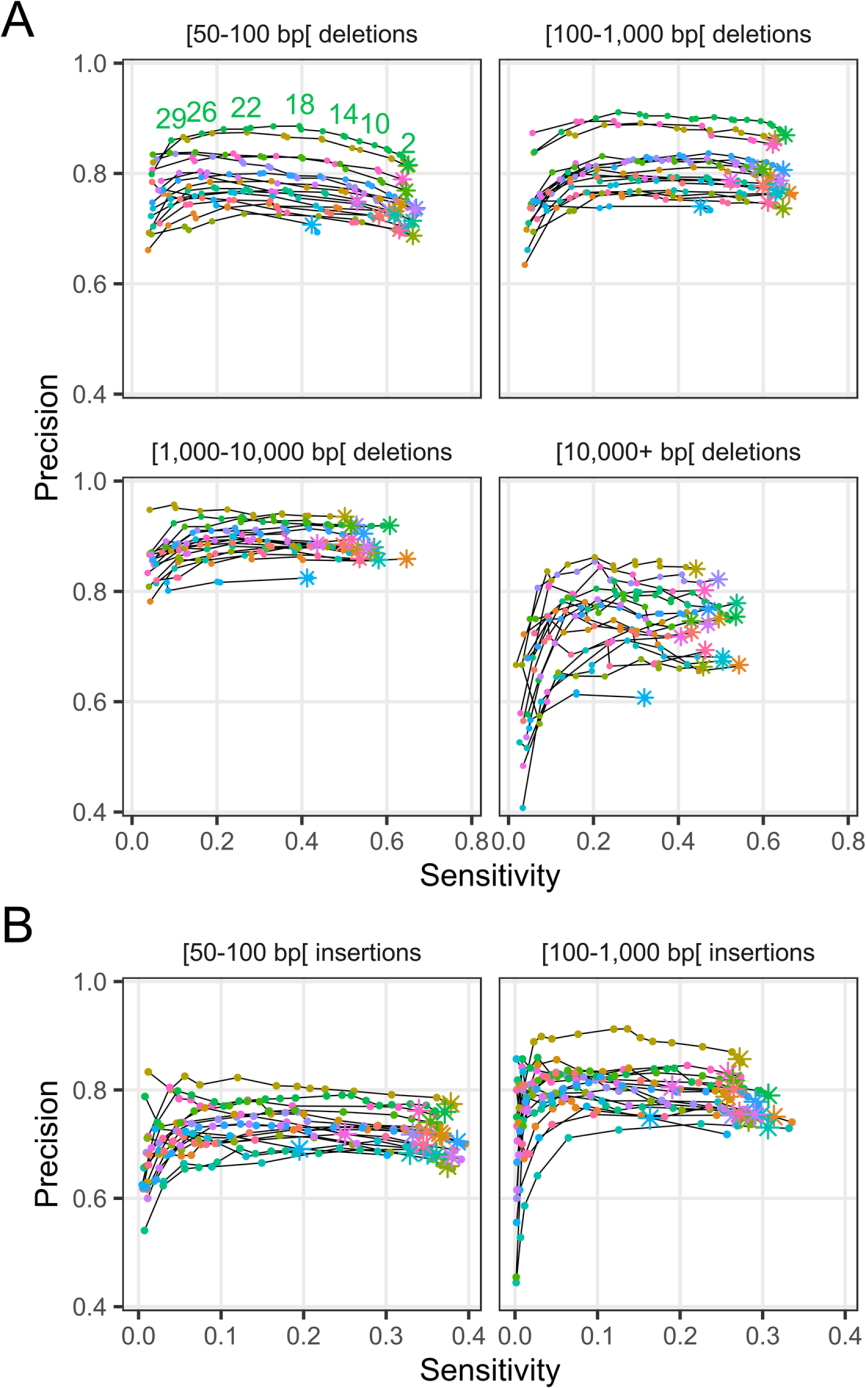
Genotyping sensitivity and precision of **A** deletions and **B** insertions discovered from the Illumina data. Sensitivity was defined as the fraction of SVs in the ground truth set (SVs called by Sniffles from Oxford Nanopore data) genotyped as the alternative allele (non-reference) from the Illumina data. Precision was defined as the fraction of SVs genotyped as the alternative allele from the Illumina data that were also observed in the truth set. Each line and color represents one of 17 samples. The different plots correspond to different SV lengths. The points correspond to different filtering thresholds on the minimum number of Illumina reads required to support a genotype call. The asterisks indicate a minimum number of supporting reads of 2; points to the left of these for a given line represent increasingly stringent filtering threshold values (i.e., a greater number of reads supporting a genotype call). Some of the threshold values for the minimum number of reads supporting a genotype call are shown for a single sample in the upper left plot of panel **A**

Results show that the genotypes of deletions and insertions could be called confidently with as few as two (2) supporting Illumina reads, which was used as a minimum threshold for all subsequent analyses (Fig. 1). At this threshold, sensitivity ranged between 50 and 65% and precision ranged from 70 to 95% for deletions, while sensitivity ranged between 30 and 40% and precision ranged from 65 to 85% for insertions (Fig. 1). Precision was typically higher for intermediate-sized deletions (100–10,000 bp) than for either extremes, while sensitivity was highest for smaller ones (50–1,000 bp). Precision was higher for larger insertions (100–1000 bp) than for small ones (50–100 bp), at the expense of lower sensitivity; virtually no insertions larger than 1 kb could be called from the Illumina data (Table 1). Sensitivity increased markedly when repetitive regions were ignored, with sensitivity increasing by up to 10–20% depending on the SV type and size class, while precision remained roughly similar (Additional file 1: Figure S3). Results for inversions showed moderate precision (in the range of 40–70%) and low sensitivity (range of 10–20%), while results for duplications showed both low precision (range of 10–20%) and sensitivity (15–20%) (Additional file 1: Figure S4). Poor performance was expected for inversions and duplications given the high complexity of those types of SVs. Excluding repeat regions did little to improve the results for duplications, but it did improve sensitivity by roughly 10% for inversions (Additional file 1: Figure S5). We observed a correlation between the Oxford Nanopore sequencing depth of a sample and the genotyping precision of deletions, insertions, and duplications for that sample, with this effect being most important for duplications (Additional file 1: Figure S6). This suggests that samples that were less deeply sequenced with long reads (< 10× sequencing depth) may have failed to reveal some SVs, thus resulting in a seemingly lower precision. Indeed, subsampling analyses performed on some samples sequenced with Oxford Nanopore showed that the number of SVs discovered started to reach a plateau at ~10× in most cases and that sequencing depth generally had no effect on their accuracy (Additional file 1: Supplemental Methods and figures S7 to S10). Assuming this plateau extends to higher coverages, most samples do provide a fairly robust dataset for benchmarking, although a few would have benefited from increased Oxford Nanopore sequencing depth.

Next, we assessed whether filtering SVs based on their frequency in the population resulted in a higher-quality SV set by removing putative false variants. Precision-recall curves computed for a range of homozygous ALT count (see “Methods” for more details) thresholds indicated that a filter based on a minimum of four alternate alleles observed across the population yielded a good compromise between sensitivity and precision for insertions and deletions (Additional file 1: Figure S11). This threshold was used to filter the set of SVs for all downstream analyses. Filtering on the homozygous ALT count did not succeed in significantly increasing the genotyping performance of duplications and inversions (Additional file 1: Figure S12), so we decided to drop these SVs from downstream analyses. We also investigated whether a filter based on the number of distinct tools reporting a SV could be used to improve sensitivity and precision (Additional file 1: Figure S13). However, the drop in sensitivity when requiring more than one tool was generally too large to compensate for the increase in precision. Researchers valuing precision over sensitivity could however use this filter, as the gain in precision was considerable in some cases, like for large deletions.

As a consequence of their different approaches to SV discovery, and consistently with the drop in sensitivity observed when requiring multiple calling tools to consider a variant (Additional file 1: Figure S13), the various tools used showed different profiles in terms of the number of variants of different sizes and types discovered (Additional file 1: Figure S14). The performance of the different tools used for calling SVs is shown for a single representative sample in figures S15 and S16 (Additional file 1). These results place Manta as the most important contributor of unique true positive SV calls for both deletions and insertions, followed by AsmVar. There is an obvious decrease in the false positive rate when combining evidence from several calling tools. However, individual tools still made significant contributions that justified their inclusion, with the exception of SvABA insertions which contributed few true positive SVs compared to the number of false positives (Additional file 1: Figure S16). SvABA insertions were still used for downstream analyses, but could be excluded for applications where the need for precision outweighs the need for sensitivity.

### Re-genotyping Oxford Nanopore-discovered variants

In addition to using the SVs discovered from the Oxford Nanopore data as a truth set for benchmarking SV discovery, we also assessed whether these could be accurately genotyped using Illumina data. For that purpose, we merged the calls made from the Oxford Nanopore data of all 17 samples using SVmerge. These were used as input to Paragraph and re-genotyped using Illumina data from the same 17 samples. The genotypes were compared to the SV calls made by Sniffles directly from the Oxford Nanopore data results using the sveval package as was done for the Illumina SVs.

**Fig. 2:**
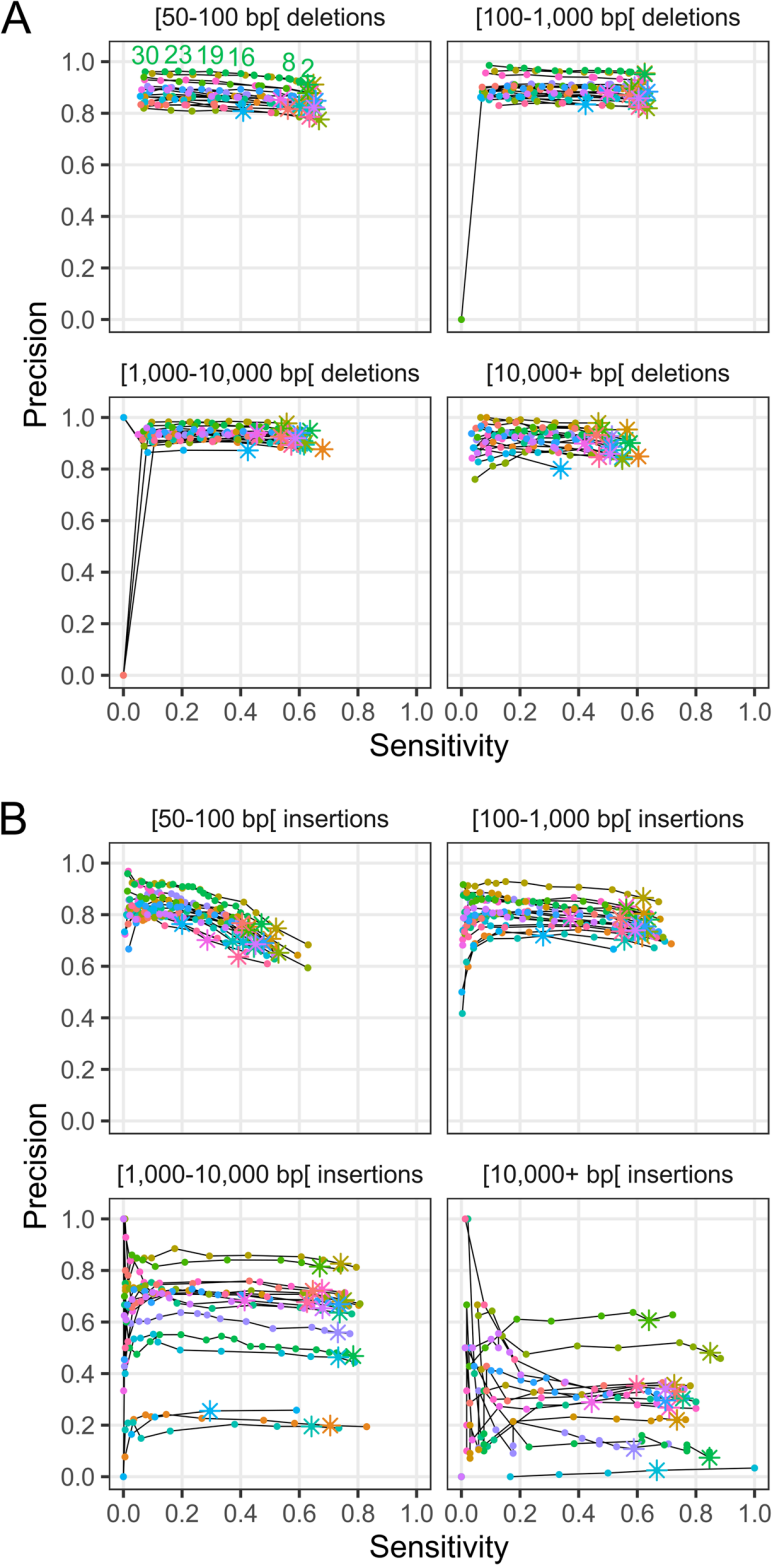
Genotyping sensitivity and precision of **A** deletions and **B** insertions discovered from the Oxford Nanopore data. Sensitivity was defined as the fraction of SVs in the ground truth set (SVs called by Sniffles from Oxford Nanopore data) genotyped as the alternative allele (non-reference) from the Illumina data. Precision was defined as the fraction of SVs genotyped as the alternative allele from the Illumina data that were also observed in the truth set. Each line and color represents one of 17 samples. The different plots correspond to different SV lengths. The points correspond to different filtering thresholds on the minimum number of Illumina reads required to support a genotype call. The asterisks indicate a minimum number of supporting reads of 2; points to the left of these for a given line represent increasingly stringent filtering threshold values (i.e., a greater number of reads supporting a genotype call). Some of the threshold values for the minimum number of reads supporting a genotype call are shown for a single sample in the upper left plot of panel **A**

As was the case for Illumina SVs, two (2) Illumina reads were sufficient to confidently call SV genotypes in most samples (Fig. 2). At this threshold, sensitivity ranged from 55 to 65% and precision ranged between 80 and 95% for deletions, while sensitivity ranged from 50 to 60% and precision ranged between 60 and 80% for smaller insertions (Fig. 2). For deletions, sensitivity and precision were fairly consistent across size classes. For insertions, however, precision varied immensely from 20 to ~80% for 1–10 kb insertions and from essentially 0 to 60% for insertions larger than 10 kb. Further analysis showed that there was a correlation between the precision of insertion genotyping in these size classes and the N50 of Oxford Nanopore reads of a given sample (Additional file 1: Figure S17). Therefore, it is likely that the poor precision observed for some samples is the result of limitations of the truth dataset rather than true genotyping errors. Indeed, larger insertions could not be validated in low-N50 samples because the small length of the reads prevented their discovery in those samples. Yet, those large insertions could still be genotyped using the Illumina data provided that they were discovered in other samples with higher N50. As was the case for variants discovered from the Illumina data, sensitivity was higher when we excluded repeat regions, with sensitivity reaching 80% in some cases (Additional file 1: Figure S18).

Duplications discovered by Sniffles showed low sensitivity and precision with both being in the 20–40% range (Additional file 1: Figure S19a). Inversions, however, could be accurately genotyped from the Illumina data, with a precision typically greater than 70%, but their sensitivity was low at about 10–20% (Additional file 1: Figure S19b). Concentrating on non-repeat regions moderately improved the results for duplications (Additional file 1: Figure 20a) but did so to a larger extent for inversions, with sensitivity reaching over 20% and precision being generally over 80% (Additional file 1: Figure S20b).

### Population-scale genotyping of the joint Oxford Nanopore-Illumina SV dataset

In order to produce a population-scale SV dataset that could be used for downstream analyses, we merged the SVs discovered from the Illumina and Oxford Nanopore data to create a candidate SV set by obtaining their union using SVmerge. The SVs in this set were subsequently genotyped with Paragraph using the Illumina data of the 102 samples. Benchmarking results for deletions and insertions expectedly showed a precision that was in-between that of the previous two benchmarks (Illumina SVs and Oxford Nanopore SVs), both when considering all regions (Additional file 1: Figure S21) and non-repeat regions only (Additional file 1: Figure S22).

The dataset was further filtered using knowledge gained from the benchmarking results presented above. Namely, we filtered out genotype calls with fewer than two (2) supporting reads and removed SVs with fewer than four (4) alternate alleles observed among homozygous genotype calls (homozygous ALT count). Inversions and duplications were also removed for downstream analyses due to their poor performance in the benchmarks. Unless otherwise noted, all downstream analyses were performed on the genotyped insertions and deletions in both repeated regions as well as non-repeated regions. For simplicity, we refer collectively to insertions and deletions as SVs in the context of downstream analyses, despite duplications and inversions being excluded.

**Fig. 3:**
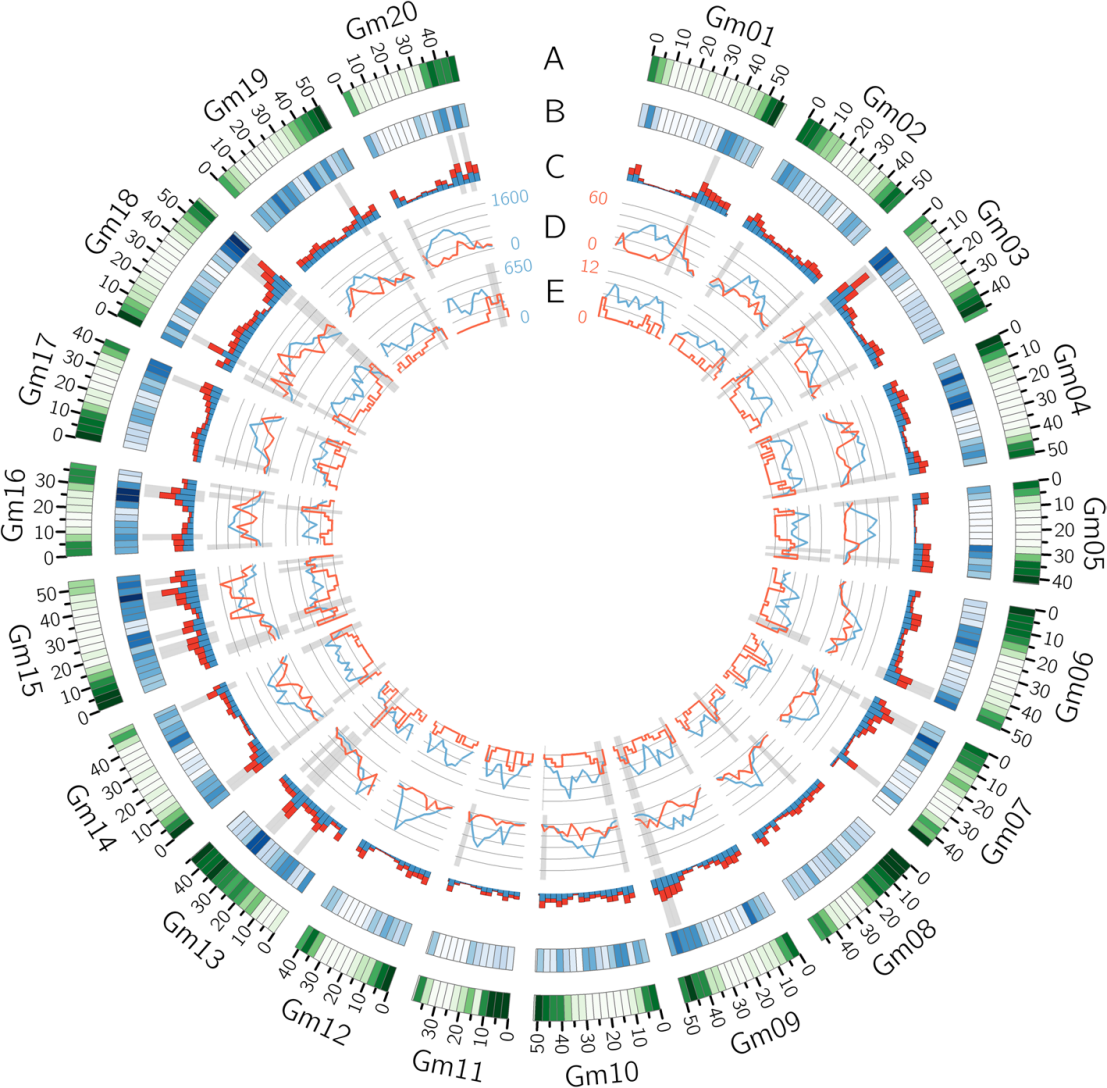
Circos plot of the distribution of various features within 3-Mb bins along the reference assembly version 4 of Williams82. Results shown are based on the population-scale (102 samples) genotyping of SVs discovered using both Illumina and Oxford Nanopore data. **A** Gene density. **B** Density of SNVs called by Platypus. **C** Number of deletions (blue) and insertions (red) discovered within each bin. The bins with the 10% highest SV density (insertions and deletions considered together) are highlighted in gray. **D** Number of reference (blue) and polymorphic (red) LTR Copia and LTR Gypsy elements (summed together). **E** Number of reference (blue) and polymorphic (red) DNA transposable elements. The gray highlights in tracks **D** and **E** show the bins with the 10% highest polymorphic/reference ratios

The distribution of deletion and insertion calls within the reference genome is illustrated in Fig. 3c. There is a visible tendency for SVs to be more frequent in gene-rich euchromatic regions (Fig. 3a) where predicted SNVs are also more densely distributed (Fig. 3b), although this may be due only to a higher discovery power in euchromatic regions. The presence of SV hotspots on chromosomes 3, 6, 7, 16, and 18 (Fig. 3c) is consistent with results previously obtained using comparative genomic hybridization by McHale et al. [34] and by a pangenome approach [37].

### Population genetics analyses

To assess the quality of our population-scale SV dataset, we verified whether population genetics patterns inferred from SVs (deletions and insertions) yielded similar results to those inferred from SNVs, which are more commonly used for population genetics inference. For this purpose, we first computed population structure using fastStructure on both the SNV and SV datasets. The fastStructure analysis based on SNVs was used to assign all individuals to one of five (5) populations based on the population with the highest ancestry (*q*-value) for each individual. Both SNV and SV datasets were also used to compute a principal component analysis (PCA) and a neighbor-joining tree for which support for nodes was assessed by 100 bootstrap iterations.

**Fig. 4:**
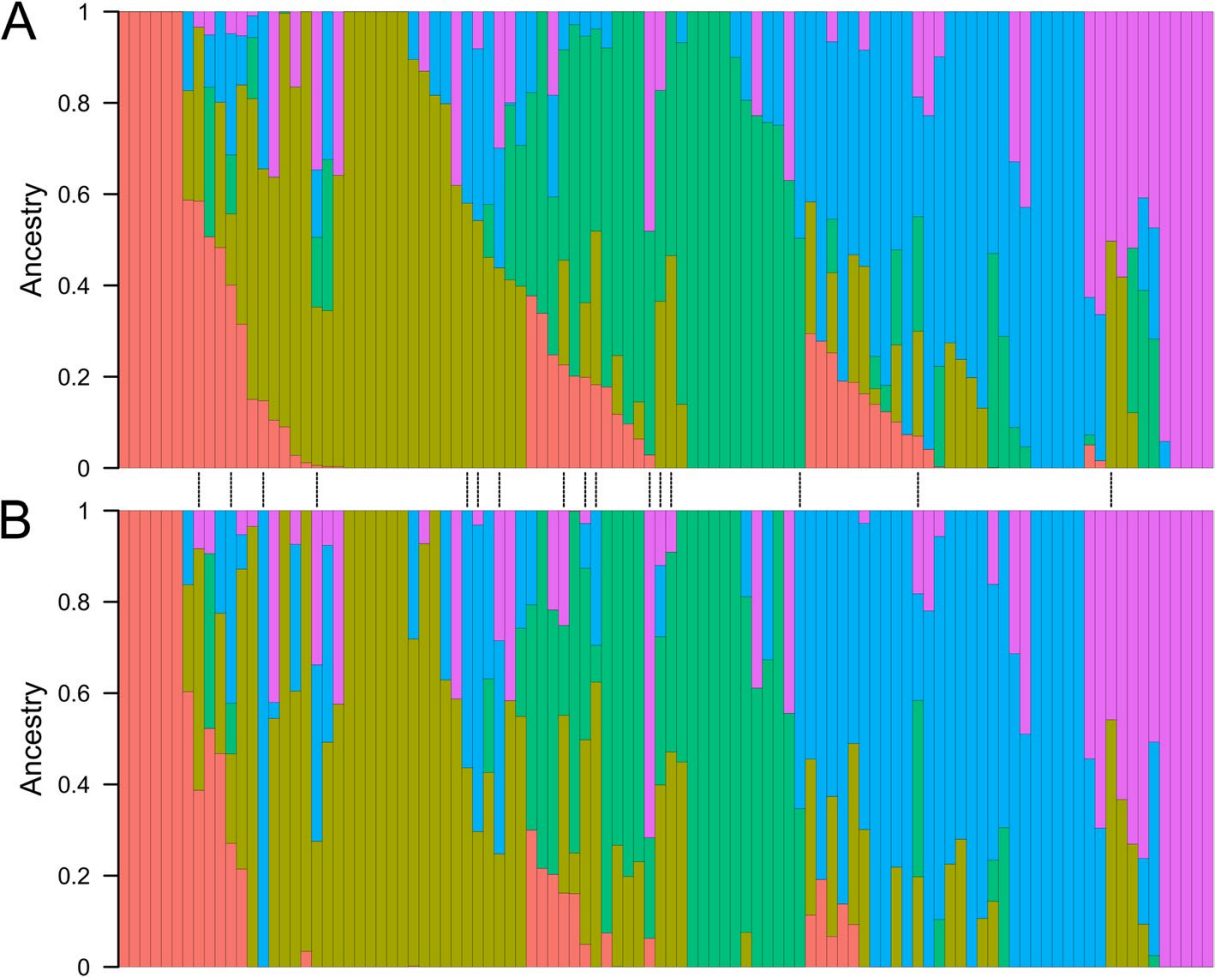
Population structure computed on all 102 Canadian soybean cultivars using fastStructure with *k* = 5 on **A** SNVs called by Platypus from Illumina data and **B** SVs discovered from Illumina and Oxford Nanopore data, and subsequently genotyped with Illumina data using Paragraph. The proportion of ancestry attributed to each of five populations is shown along the *y*-axis for 102 cultivars displayed along the *x*-axis. The order of the cultivars and the color scheme are identical in both panels. The vertical dotted lines between panels denote the 16 cultivars for which the assigned population (i.e., the population with the highest ancestry for that cultivar) differs

Population structure analyses yielded very similar results for the SNV and SV datasets, with only 16 out of 102 samples not being assigned to the same population by both analyses (Fig. 4). Out of these 16 samples, none had been assigned to a population with > 0.6 ancestry by the SNV analysis, suggesting that all individuals with clear ancestry were classified similarly by the SNV and SV analyses. The PCAs did not cluster the samples belonging to different populations into starkly distinct groups because the panel under study does not display a strong structure to begin with. Still, both the SV (Additional file 1: Figure S23a) and the SNV PCA (Additional file 1: Figure S23b) roughly grouped individuals according to their assigned population. Moreover, the PCA made from the SV genotype calls was at least as good at clustering together the samples belonging to the same population as the PCA made using SNVs was. Neighbor-joining trees made from SNVs and SVs similarly grouped samples roughly according to their population assigned by the SNV fastStructure analysis (Additional file 1: Figure S24). Out of the 100 nodes in the SNV phylogenetic tree, 36 were found in the SV phylogenetic tree and 57 were found in at least one bootstrap iteration of the SV tree. A two-sided Mantel test with 999 permutations computed on the distance matrices obtained from SNVs and SVs also found a significant correlation between both matrices with a Z-statistic of 560.8 and a *p*-value of 0.001. Overall, these results constitute a proof of concept that the population-scale SV dataset is the reflection of a biological reality and not an artifact.

### Potential impact on genes

**Fig. 5:**
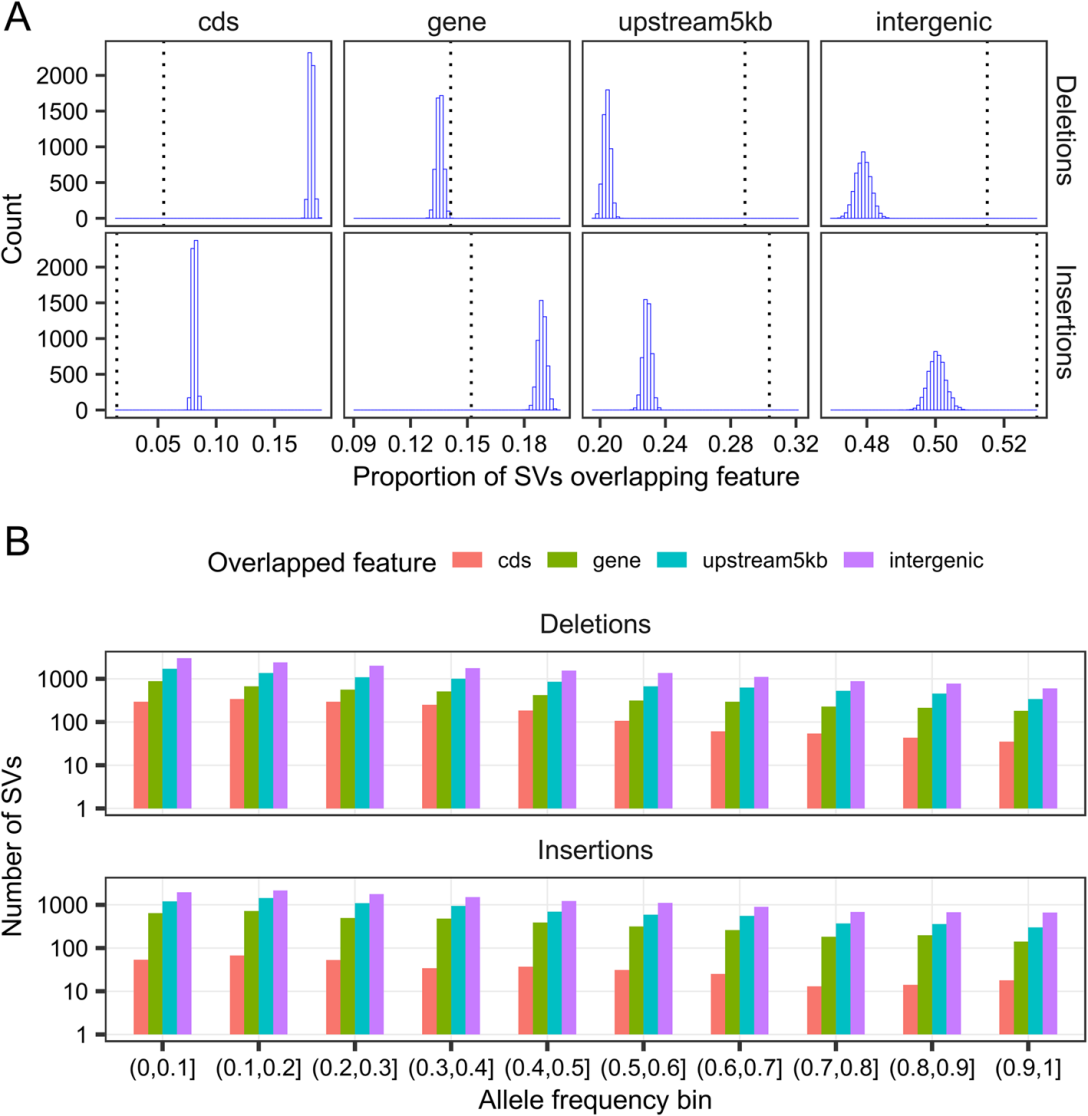
Analysis of the overlap of SVs with gene models. **A** Distributions of the proportions of deletions and insertions overlapping various genic features as generated by a randomization test (5000 iterations). Observed proportions for each SV type and genic feature are indicated by a vertical dotted line. One-sided *p*-values are < 2 × 10^−4^ for all comparisons except for deletions overlapping genes, for which the *p*-value is 4 × 10^−4^. **B** Distribution of the allele frequencies of deletions and insertions depending on the genic features they overlap. Note the logarithmic scale on the *y*-axis. cds: SVs overlapping coding sequences; gene: SVs overlapping non-coding genic sequences; upstream5kb: SVs overlapping regions 5 kb upstream of genes, but not any genic sequences; intergenic: SVs that do not overlap any of the other features

SVs can have a large impact on gene integrity or expression. Therefore, we annotated the SVs (deletions and insertions) in our dataset according to the genic features they overlapped. SVs occurred disproportionately less within coding sequences than would be expected based on the proportion of the genome covered by these features, both when considering the whole genome and when restricting the analysis to non-repeat regions (Additional file 1: Table S2). A slight underrepresentation of SVs was also observed within non-coding genic sequences, although this pattern was much clearer when concentrating on non-repeat regions. Both analyses also revealed a clear pattern of overrepresentation of SVs within regions 5 kb upstream of genes. The proportion of SVs overlapping intergenic regions appeared to be less than expected when the analysis was performed on the whole genome, but this is most likely due to the fact that intergenic regions tend to be more repetitive and thus more difficult to probe. Indeed, when restricting the analysis to non-repeat regions, the proportion of SVs falling within intergenic regions was higher than their proportion within the reference genome, suggesting enrichment of SVs. We also compared the observed proportions of SVs overlapping various genic features to what would be expected by random chance using a randomization test that shuffled the positions of SVs within 100-kb bins and computed the resulting overlaps. The 100-kb bins were used to locally restrict the SV position to take into account the repeat heterogeneity of the genome. This test confirmed the underrepresentation of SVs within coding sequences and their overrepresentation within intergenic sequences and regions 5 kb upstream of genes (Fig. 5a). The pattern for non-coding genic sequences, however, diverged from other lines of evidence by suggesting slight overrepresentation of deletions. Insertions, on the other hand, appeared to be underrepresented within non-coding genic sequences, similar to the results shown in Table S2 (Additional file 1).

The distributions of insertion and deletion frequencies depending on the features overlapped are shown in Fig. 5b. Statistical testing of the pairwise differences in mean SV frequencies depending on the genic features overlapped clearly showed that deletions overlapping coding sequences were less frequent (the frequency being lower by roughly 0.05) than those occurring elsewhere in the genome (Additional file 1: Table S3). For insertions, the only significant differences indicated a higher frequency (by roughly 0.02) in intergenic regions than in non-coding genic sequences or sequences 5 kb upstream of genes. A difference of similar magnitude was also observed between mean insertion frequency within intergenic regions and coding sequences, but the difference was marginally non-significant.

Finally, we conducted an enrichment analysis to check for over- and underrepresentation of gene ontology (GO) Biological Process terms and PFAM protein domains in genes whose coding sequence is impacted by SVs that are frequent (≥ 0.5) in the population. Genes impacted by high-frequency SVs were highly enriched for functions involved in defense response, and somewhat less so for functions involved in the regulation of various pathways (Additional file 1: Table S4; Additional file 2). Underrepresented GO Biological Process terms were almost all related to various metabolic or biosynthetic processes (Additional file 1: Table S5; Additional file 3). As was observed for GO Biological Process terms, the PFAM domain enrichment analysis showed that genes impacted by high-frequency SVs are overwhelmingly enriched in domains involved in defense response, such as NB-ARC, TIR, and Leucine-rich repeat domains (Additional file 1: Table S6; Additional file 4). No PFAM domains were observed to be underrepresented (Additional file 5).

### Transposable elements

Many SVs, especially larger ones, result from the mobilization of TEs [12, 55]. With this in mind, we checked whether we could gain insights into soybean TE biology from our SV dataset. To do so, we first queried the sequences of all insertions and deletions larger than 100 bp in our dataset against a database of soybean TEs. Insertions and deletions that matched a TE with high confidence were annotated with the corresponding TE type.

**Fig. 6:**
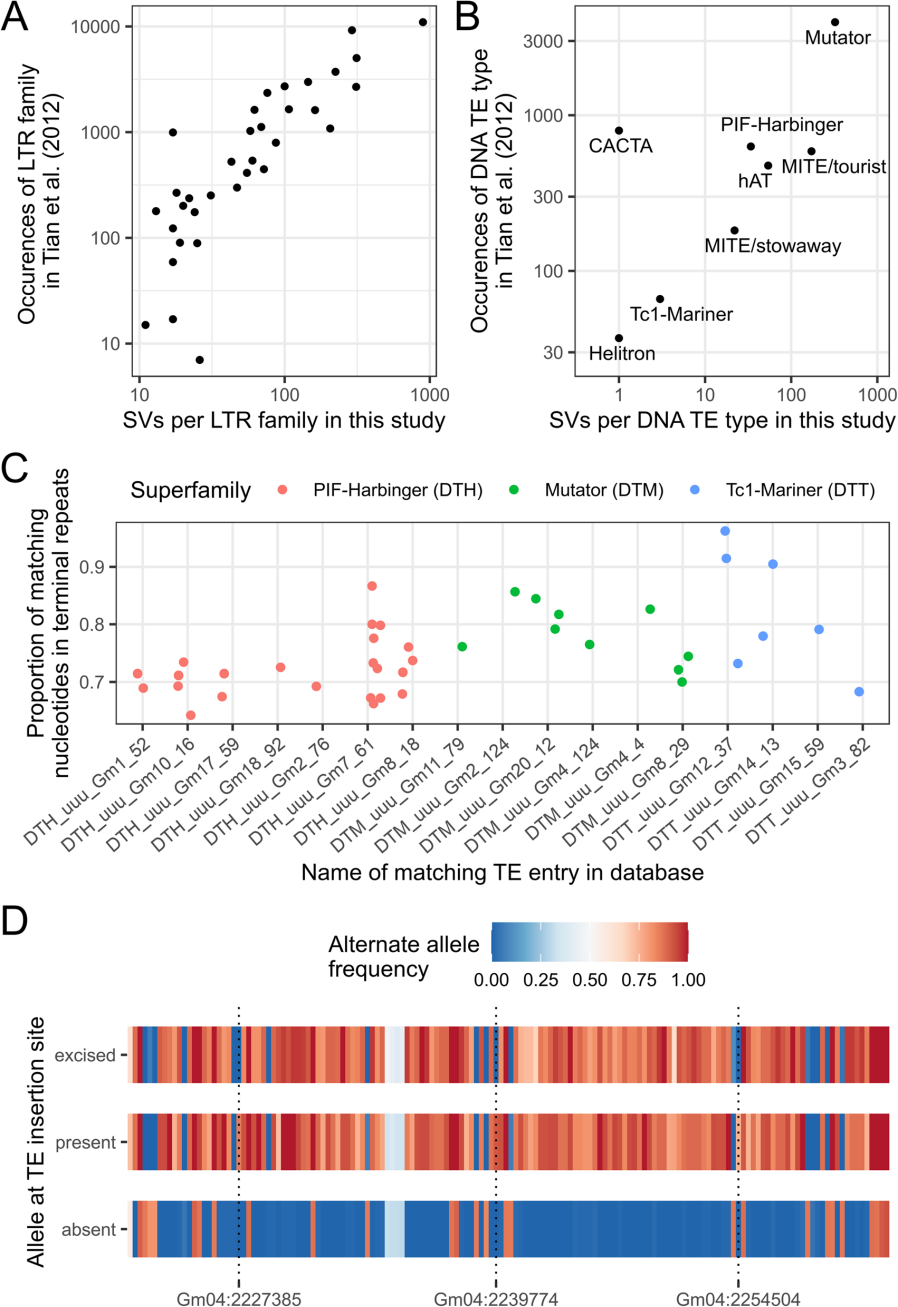
Analysis of the polymorphic TEs found in this study. Comparison of the number of polymorphic TEs per **A** LTR family and **B** DNA TE type found in Tian et al. [38] and in this study. Differences in *y*- and *x*-scales are partly explained by the fact that counts for Tian et al. are summed over occurrences in all samples whereas our data counts each SV only once. Note that all scales are logarithmic. **C** Proportion of matching nucleotides between the two terminal repeats for TE sequences corresponding to 40 different SVs grouped by DNA TE superfamily and by the identifier of the TE sequence they matched in the SoyTEdb database. **D** Alternate allele frequencies of 156 SNVs located in a ~39-kb linkage disequilibrium block between positions Gm04:2,220,398 and Gm04:2,259,326. Frequencies were computed for three different groups of samples depending on their genotype at the TE insertion site (Gm04:2,257,090). absent: absence of the TE insertion, which corresponds to the reference allele (71 samples); present: presence of the 480-bp Stowaway MITE (9 samples); excised: presence of a 6-bp insertion at the insertion site, putatively left by excision of the TE insertion (14 samples). The locations of three SNVs whose frequency in the “present” and “excised” groups diverge are shown with dotted vertical lines

A total of 2586 deletions and 2391 insertions were annotated as TEs by this approach (Table 2; Fig. 3d,e; Additional file 6). These represent 8.4% and 9.1% of all deletions and insertions, respectively, and 14.9% and 17.4% of those larger than 100 nucleotides. The proportion of polymorphic TEs of different classes found within our dataset is consistent with their prevalence in the reference genome, except for DNA TEs which represent a much smaller proportion of the polymorphic elements compared to their prevalence in the genome. The number of polymorphic elements per LTR retrotransposon family (Fig. 6a) and per DNA TE type (Fig. 6b) were largely consistent with results previously reported for non-reference soybean TEs [38] except for DNA TEs of the CACTA superfamily for which we found almost no polymorphic instances.

**Table 2:** Number and span of polymorphic and reference transposable elements of different types

	REF^a^ (%)				DEL^b^ (%)				INS^c^ (%)			
TE type	*N*^d^		Mb^e^		*N*		kb^f^		*N*		kb	
Copia LTR retrotransposons	91,241 (35.1)		170 (43.0)		1154 (44.6)		5594 (43.8)		1303 (54.5)		6692 (63.1)	
Gypsy LTR retrotransposons	71,390 (27.5)		139 (35.2)		949 (36.7)		5745 (45)		718 (30)		2949 (27.8)	
Non-LTR retrotransposons	8078 (3.1)		10 (2.5)		144 (5.6)		449 (3.5)		99 (4.1)		307 (2.9)	
DNA TE	89,300 (34.3)		76 (19.2)		339 (13.1)		989 (7.7)		271 (11.3)		654 (6.2)	

^a^ REF: transposable elements ≥ 100 bp in the reference genome

^b^ DEL: deletions relative to the reference that are annotated as TEs

^c^ INS: insertions relative to the reference that are annotated as TEs

^d^ *N*: number of reference elements, deletions or insertions matching given TE type

^e^ Mb: total length of reference elements of a given type, in Mb

^f^ kb: total length of polymorphic elements matching given TE type, in kb

We identified terminal inverted repeat (TIR) and target site duplication (TSD) sequences from local assemblies of the TE sequences for 40 different polymorphic SVs collectively representing 17 entries in the SoyTEdb database. The polymorphic TEs for which we could identify TIR and TSD sequences were essentially miniature inverted-repeat transposable elements (MITEs) ranging in size from 198 to 681 bp (longer sequences were too challenging to assemble properly). From these data, we computed the proportion of matching nucleotides between the two inverted repeats and averaged the values over the all samples bearing the TE insertion for a given SV (Fig. 6c). A high proportion of matching nucleotides can indicate the potential for active transposition because intact transposons should have identical or nearly identical TIRs. While the proportion of matching nucleotides was under 0.8 in most cases, three polymorphic TEs matching the Tc1-Mariner superfamily and annotated as Stowaway MITEs presented a proportion of matching nucleotides > 0.9 (Additional file 7).

We generated multiple alignments of the local assemblies at all sites where at least one sample had recognizable TIR and TSD sequences. A visual analysis of these multiple alignments revealed that for all but one SV, the sequences that did not bear the insertion presented a single occurrence of the TSD sequence. This observation is consistent with a scenario where the TE never inserted into the sequence, instead of having excised from it. The one exception to this observation is that of a 480-bp insertion of a Stowaway MITE at position 2,257,090 of chromosome Gm04. In this case, a visual analysis of the multiple alignment revealed that three different alleles are segregating in the population at the insertion site: (1) the reference allele (no insertion at the target position), (2) a 480-bp insertion that corresponds to the TE insertion, and (3) a 6-bp insertion of nucleotides TACGAG (Additional file 1: Figure S25; Additional file 8). Interestingly, this insertion is by far the one for which the percent similarity between the two TIR sequences was highest among the ones studied, at 96.3%. We hypothesized that the 6-bp insertion resulted from the excision of the TE, with the TA nucleotides being remnants of the classical Tc1-Mariner TSD and the other nucleotides having been added during DNA repair following excision. If this is the case, then the haplotypes surrounding the insertion site should be very similar between the individuals with the TE insertion and those with the 6-bp insertion. Using a combination of SV calls made by Paragraph and indel calls made by Platypus, we assigned 71 individuals as homozygous for the reference allele, 9 individuals as homozygous for the TE insertion allele, and 14 individuals as homozygous for the 6-bp insertion allele. We computed the alternate allele frequencies within each of these three groups for 156 SNVs located in a 39-kb linkage disequilibrium block surrounding the insertion site (Fig. 6d). The results clearly show high genetic similarity between individuals bearing the TE insertion and those bearing the 6-bp insertion, consistent with the latter being derived from excision of the TE insertion. In fact, only three (3) SNVs showed contrasting allele frequencies (difference in allele frequencies > 0.5) between these two groups (Fig. 6d), whereas 129 alleles were contrasted between the reference allele haplotype and the TE insertion allele haplotype. This suggests that the excision of the TE is a relatively recent event and that this TE may still be active in soybean.

Interestingly, one of the polymorphic *Copia* insertions found in our dataset matches an insertion in the Glyma.20G090000 gene (also known as the PhyA2 gene corresponding to the E4 maturity locus) known to impact time to maturity in soybean [56]. In our dataset, this TE insertion had a frequency of 0.207, with 20 samples genotyped as homozygous for the alternative allele and a single one genotyped as heterozygous.

## Discussion

The rapid development of long-read sequencing platforms such as PacBio and Oxford Nanopore in recent years has greatly enhanced the potential for studying structural variation. Although studies using long reads to survey structural variation in crops have started to emerge (e.g., [12, 27, 28]), they did not explicitly address the question of using short reads to scale up SV analysis from the small cohorts sequenced using long reads to larger populations, as has been done in humans (e.g., [57, 58]). This question is of interest because long-read sequencing remains too expensive at the moment to apply at a large scale and because large amounts of already-existing short-read sequencing data could be leveraged in that way. Scaling up the study of SVs is a necessary prerequisite to getting a clear understanding of genome evolution and function, and applying this knowledge to real-world problems [1, 15]. In this study, we demonstrate that a relatively small cohort of 17 samples sequenced to ~12× coverage with Oxford Nanopore can be combined with Illumina data to drive the study of SVs in a population of 102 Canadian soybean lines and gain insights into SV biology.

In this work, benchmarks of SVs discovered from Illumina data alone revealed sensitivity rates between 50 and 65% and precision rates between 70 and 95% for deletions, while sensitivity ranged between 30 and 40% and precision ranged from 65 to 85% for insertions. While these results may appear disappointing, they are in line with large-scale benchmarks performed on short-read SV callers and are consistent with known limitations to SV calling from short reads [18, 20]. Our benchmarking results for inversions and duplications proved to be so unsatisfactory that we decided not to consider them at all in downstream analyses, but again such poor results were expected based on previously published benchmarks [20]. For applications where precision is to be valued over sensitivity, our benchmarks provide guidance as to how combining evidence from various SV callers or concentrating on non-repeated regions improves the accuracy of SV genotyping.

We called SVs based on relatively low-depth Illumina sequencing, with effective sequencing depth ranging between 5× and 15× for most samples (Additional file 1: Table S7). While deeper coverage would necessarily have resulted in higher sensitivity and precision, at least two reasons explain how we could achieve reasonably good performance despite limited coverage. First, the inbred status of the cultivars studied makes it easier to call variants because all reads originating from a genomic location where a variant is located should support the existence of that variant, instead of only half of the reads in the case of heterozygous genotypes. This also makes it possible to confidently call the genotype of a variant as homozygous from only a few reads. Second, the two-step approach adopted here (discover SVs on all samples first, and genotype them using a dedicated SV genotyper afterwards) made it possible to efficiently combine information across all samples. Therefore, a variant could be genotyped from as few as two reads in a given sample even if the coverage at that variant’s position did not allow it to be discovered in that sample in the first place. The results presented in this study therefore show that low Illumina sequencing depth should not limit the population-scale study of SVs as long as appropriate methods for SV discovery and genotyping are used.

We were especially interested in calling and genotyping large (> 1 kb) insertions given the potential of long reads to relieve the inherent limitation of short reads to span large repeats and effectively assemble into long insertions. Indeed, for insertions discovered from short reads only, estimated sensitivity was low at ~ 40% for those in the range 50–100 bp and ~ 30% for those in the range 100–1000 bp; virtually no insertions larger than 1000 bp were discovered. The improved sensitivity obtained when focusing on non-repeated regions (up to ~ 60% for insertions in the range 50–100 bp) shows that a large part of the problem indeed comes from repeated regions. However, entirely removing these regions from analyses is an unsatisfactory solution as polymorphisms in these regions may still be relevant to a particular study question. To compensate for limitations in SV discovery from short reads, we assessed whether Illumina reads could be used to genotype SVs discovered from Oxford Nanopore data on a smaller cohort of 17 samples. The greatest added value of this approach arguably comes from the possibility to accurately genotype large (> 1 kb) insertions in many samples without having to sequence all samples using more costly long-read sequencing. This is an encouraging result because it shows that such insertions can be successfully genotyped using Illumina data even though they could not be discovered from this same data. This is because long reads provide the full contiguous sequence of insertions, which the Illumina reads can then map to. Combined with a novel pipeline for refining the breakpoints and sequence content of SVs discovered from Oxford Nanopore sequencing data prior to genotyping, this approach should enable the study of SVs in large populations for which short-read data is already available.

Reliably benchmarking the performance of SV genotyping depends on the existence of a gold standard truth dataset, which is the main limitation of our study as there exists no such standard yet for soybean. Our ground truth dataset was based on SVs called from Oxford Nanopore data only using a single SV caller, Sniffles. While Sniffles was warranted for this purpose based on published benchmarks [26], combining other SV callers and data types (such as PacBio sequencing or optical mapping) may have resulted in a more accurate ground truth dataset. Indeed, Oxford Nanopore reads alone do not provide a perfect truth for benchmarking, especially for SVs under 100 bp [2]. This limitation was apparent for some of the results observed in our study. For example, some of the samples appeared to have low genotyping precision for larger insertions, but this was most likely due to these insertions not being discovered in samples with lower read N50 and thus appearing as false positive genotype calls. Similarly, the sequencing depth of the Oxford Nanopore data used here was not sufficient to provide a solid reference dataset for benchmarking duplications. Nevertheless, subsampling analyses and the analysis of the relationship between Oxford Nanopore sequencing depth and genotyping precision revealed that samples sequenced at 10× depth or higher provided sufficient coverage for the discovery of deletions and insertions, the two most abundant types of SVs. Moreover, as expected, the proportion of false positive calls by Sniffles did not vary with sequencing depth for these SVs, therefore providing robust results for use in benchmarks. For the few samples with < 10× sequencing depth, however, lower sequencing depth did result in fewer SVs being discovered in the Oxford Nanopore data and thus an apparent drop of genotyping precision in those samples.

Follow-up analysis on our population-scale SV dataset confirmed that this dataset reproduced previously described population genetics patterns, a validation approach commonly used in other population-scale SV studies (e.g., [59, 60]). We indeed found that a structure analysis yielded highly similar results whether it was computed from SVs or SNVs. Similarly, a PCA and a phylogenetic tree based on SVs summarized the population structure just as well as the same analyses based on SNVs. Together, these results suggest that the SV genotype calls on the 102-sample population are accurate and can be used to draw inferences on population structure just as well as SNVs.

Perhaps more importantly, the SV dataset produced here met our expectations regarding the genome-wide distribution of SVs and their location relative to predicted gene models. The location of SV hotspots found here is consistent with previously reported results [34, 37]. Moreover, GO term and PFAM domain enrichment analyses confirmed previous observations that SV-enriched genes are involved in plant defense response [33, 34, 37]. Several lines of evidence in our results also suggest a strong functional constraint on the location of SVs in the soybean genome. Notably, SVs were strongly depleted within coding sequences compared to what would be randomly expected, and insertions were depleted within non-coding genic sequences. There was also a clear tendency for enrichment of SVs in regions upstream of genes, but whether this is simply due to lower functional constraints or a role of SVs in regulating gene expression remains to be investigated. Functional constraints on the frequency of SVs could also be observed from our data, as deletions impacting coding sequences were less frequent than those occurring elsewhere in the genome and insertions were enriched within intergenic regions, which are arguably less functionally important. Based on these results, we suggest that many of the deletions located within coding sequences may have a deleterious impact and could therefore become targets for breeding.

The large insertions and higher power of SV discovery within repetitive regions that was afforded by the Oxford Nanopore sequencing data gave us an opportunity to study soybean TE biology more deeply than previous reports. The numbers of TEs associated with various superfamilies was largely consistent with results previously reported by Tian et al. [38], except for DNA TEs of the CACTA superfamily which were a lot less common in our data. We observed the same pattern of general concordance with previously reported results except for CACTA elements when comparing our data to that of Istanto [61]. The reason why we found almost no polymorphic CACTA elements compared to these studies is unclear, but we hypothesize that it may be due to our more stringent requirements for TE annotation. Indeed, we required the length of the queried SVs to be close to that of their matching counterpart in the database. Many of the SVs in our dataset indeed matched CACTA elements following the BLASTN query, but almost all of them failed to pass the filter. Our annotation results are probably conservative for other types of TEs as well because the database we used is likely incomplete, as it is based on the analysis of a single reference genome.

Our data also allowed us to generate original findings related to DNA TEs in soybean, which have received relatively little attention from past studies. We report results that suggest that most DNA TE insertion polymorphisms in soybean result from past insertion of TEs rather than from excision of existing TEs. The relatively low proportion of polymorphic DNA TEs compared to their prevalence in the genome also suggests that these elements are overall fairly inactive in soybean. However, we did document one case in which recent excision of a Stowaway MITE from its insertion site appears to have occurred, such that three alleles (the reference allele without the insertion, the TE insertion, and the allele resulting from the excision of the TE) are present within the population. This element represents a prime candidate to study the potential activity of DNA TE transposons in soybean.

## Conclusions

In conclusion, our study shows that Oxford Nanopore and Illumina sequencing data can be efficiently combined to study structural variation in soybean. In particular, large insertions that cannot be discovered from short-read data alone could be genotyped using short-read data and thus allow the insights gained from long-read sequencing to scale up to a larger population. This framework, combined with a novel pipeline for refining the SVs discovered using Oxford Nanopore data, should extend easily to other species and allow the wealth of already-existing Illumina data to be leveraged for SV analysis. In addition to confirming previous results regarding the chromosomal distribution of SVs in soybean and their association with genes involved in defense response, we also report novel insights into functional constraints to the occurrence of SVs and into soybean TE biology. Moreover, the SV catalog described here is freely available and can be used as a resource for SV genotyping by the soybean research community. Beyond the work presented here pertaining to SV discovery from Oxford Nanopore data specifically, we envision that genetic variation discovered by studies such as ours and others (e.g., [32, 36, 37]) could eventually be integrated in a single pangenome for soybean. Together with emerging tools for using pangenomes as a reference, such as Giraffe and the associated vg toolkit [57], this should help further the study of complex topics such as structural variation and transposable elements.

## Methods

### Illumina sequencing and read processing

Sample selection and acquisition of Illumina sequencing data has been described in previous work [6]. Briefly, 102 Canadian soybean cultivars and breeding lines were selected to encompass the full range of genetic variation found among Canadian short-season germplasm and sequenced on the Illumina HiSeq 2500 platform. Paired-end reads ranging in size from 100 to 126 nucleotides were obtained depending on the sample (see Additional file 1: Table S7 for metadata on Illumina sequencing data). This sequencing data is available on the NCBI Sequence Read Archive (SRA) through BioProject accession number PRJNA356132 [62].

All reads were adapter- and quality-trimmed using bbduk from the BBtools suite v. 38.25 [63]. We aligned reads using bwa mem v. 0.7.17-r1188 [64] with default parameters. Paired-end alignment mode was used except for reads that were left unpaired following adapter and quality trimming, which were aligned in single-end mode. We used a reference genome consisting of assembly version 4 of the Williams82 reference cultivar [65] concatenated with reference mitochondrion and chloroplast sequences retrieved from SoyBase [66]. Reads aligned using paired-end and single-end mode were then merged, sorted, and indexed using samtools v. 1.8 [46] and read groups were added using bamaddrg [67]. The sorted and indexed BAM files were used as input for all downstream analyses requiring mapped reads.

### Structural variation discovery from short reads

We called SVs on all 102 samples using four different tools: AsmVar [68], Manta [69], SvABA [70], and LUMPY-based [71] smoove [72]. We selected this combination of tools based on the complementarity of their SV detection approaches, widespread use within the community, and performance reported in published benchmarks. Manta and LUMPY (on which smoove is based) performed consistently well across various published benchmarks [18–20]. SvABA also performed well in the benchmarks published by Kosugi et al. [20]. Finally, we also chose AsmVar because of its purely assembly-based approach (contrasting with the other tools) and its use in a previous large-scale variation discovery project [73]. When calling SVs, we limited the analysis to deletions, insertions, inversions, and duplications since their alterations in sequence can be precisely represented to allow genotyping from the alignment of sequence reads. While we did not explicitly study translocations, alterations in sequence that result from a translocation may still appear in the dataset as an insertion at one genomic location and a deletion at another, even if they are not annotated as such. Similarly, we did not explicitly study copy number variants, but variants defined under this umbrella term are still included in our dataset as deletions and duplications.

AsmVar calls SVs by comparing de novo genome assemblies to a reference genome. Prior to assembly, we merged reads that were still paired after trimming using FLASH v. 1.2.11 [74]. The rationale behind this was that the short size of the inserts in our sequencing data allowed several of the read pairs to be merged into longer sequences. Reads were grouped into three libraries (single-end reads from bbduk, single-end reads merged by FLASH, and paired-end reads left unmerged by FLASH) and assembled with SOAPdenovo2 v. 2.04 [75] using the sparse_pregraph and contig commands, and a k-mer size of 49. Contigs were not further assembled into scaffolds because we aimed to only call SVs whose sequence was entirely resolved. The resulting contigs were aligned to the reference genome using LAST v. 1047 [76] by first calling the lastal command with options -D1000 -Q0 -e20 -j4 and then the last-split command with options -m 0.01 -s30. Variants were called on the LAST alignments using ASV_VariantDetector from the AsmVar tool suite (version of 2015-04-16) with default parameters. The pipeline was run on each sample independently and results were subsequently concatenated to obtain a single AsmVar VCF file. Variants with a FILTER tag other than “.” were filtered out from the resulting call set.

We ran manta v. 1.6.0 with default parameters in 10 batches of 10 or 11 randomly grouped samples because it did not scale well to the whole population. We used the candidate SVs (and not the genotype calls themselves) identified by each run for further processing and filtered them by removing unresolved breakends (SVTYPE=BND). The filtered variants were then converted from symbolic alleles (i.e., DEL, DUP, INS) to sequence-explicit ALT alleles using bayesTyperTools convertAllele v. 1.5 [31] and combined into a single VCF file using bcftools merge (version 1.10.2-105) [46].

We ran SvABA v. 1.1.3 separately on all samples using the command svaba run with options --germline -I -L 6. SvABA produces two different variant sets: one for indels, which are already coded as sequence-explicit, and another for SVs which are coded as paired breakends. We therefore classified SVs into defined types (DEL, DUP, INV) based on breakpoint orientation and converted them to sequence-specific ALT alleles using an in-house R script. The resulting sequence-explicit variants were merged using bcftools merge.

We ran smoove v. 0.2.4 on all samples using a series of commands. First, smoove call was run separately on each sample using default parameters. The variants identified were then merged into a single VCF file using smoove merge, smoove genotype with options -x -d, and smoove paste. Symbolic alleles (<DEL>, <DUP>, and <INV> alleles) were converted to explicit sequence representation using bayesTyperTools convertAllele.

A series of common filters were applied to the SV output of all four tools before using them for downstream analyses. Specifically, we removed variants spanning less than 50 bp or more than 500 kb, those located on unanchored scaffolds or organellar genomes, or any variant that was not classified as either a deletion, insertion, duplication, or inversion. The 500-kb threshold was chosen to ensure computational efficiency in downstream applications, but SVs larger than these are also expected to be very rare in soybean germplasm [32]. We also converted multiallelic variants into biallelic records and standardized the representation of all alleles using bcftools norm.

### Oxford Nanopore sequencing

We selected 17 samples for Oxford Nanopore sequencing among those sequenced by Illumina. Sixteen (16) of them were randomly selected among a subset of 56 lines belonging to a core set of Canadian soybean germplasm, while the remaining sample (CAD1052/OAC Embro) had been selected and sequenced before the others based on its higher Illumina sequencing depth. Although sample selection did not explicitly maximize the number of potential SVs assessed, we did verify that the resulting set covered the range of variation found in Canadian soybean germplasm based on an existing phylogenetic tree [6].

Our sample preparation and sequencing protocols evolved throughout the project as we gained experience with Oxford Nanopore sequencing. Therefore, we outline our latest methods here, but more details regarding the procedures used for each sample can be found in Table S8 (Additional file 1). Accessions selected for sequencing were germinated in Jiffy peat pellets (Jiffy Group, Zwijndrecht, Netherlands) on the benchtop. Young trifoliate leaves were collected between 2 and 3 weeks after germination, flash frozen in liquid nitrogen upon harvest, and stored at − 80 °C until DNA extraction. Single trifoliate leaves weighing between 20 and 60 mg were used for each extraction. Liquid nitrogen-frozen leaves were pulverized on a Qiagen TissueLyser instrument (Qiagen, Hilden, Germany) with metal beads for four cycles of 30 s each at 30 Hz. The resulting powder was immediately transferred to a CTAB buffer (2% CTAB, 0.1 M Tris-HCl pH 8, 0.02 M EDTA pH 8, 1.4 M NaCl, 1% (m/v) PVP) and incubated at 60 °C in a water bath for 45 min. The lysate recovered after centrifugation at 3500 rcf for 10 min was then subjected to an RNase A treatment for another 45 min at 60 °C, followed by the addition of an equal volume of 24:1 chloroform:isoamyl alcohol to the sample and stirring to an emulsion. Following centrifugation at 3500 rcf for 15 min, the supernatant was recovered and mixed with a 0.7 volume of cold isopropanol. This mix was stored at – 80 °C for 20 min and centrifuged at 3500 rcf for 30 min, after which the liquid was removed. Tubes were rinsed twice with cold 70% ethanol, with a centrifugation step after each addition of ethanol. After the last rinsing, tubes were left to dry for 3 min after which pellets were resuspended in 100 μl elution buffer (Tris-HCl 0.01 M and EDTA 0.001 M, pH 8) at 37 °C for an hour, and then stored at 4 °C until use.

Samples were size-selected using the Short Read Eliminator kit of Circulomics (Circulomics, Baltimore, MD, USA) following the manufacturer’s instructions. The size-selected DNA resuspended in the SRE kit’s EB buffer was then purified using SparQ magnetic beads and resuspended in ddH_2_O. Typically, between 500 ng and 1 μg of this DNA was used for Oxford Nanopore library preparation using the SQK-LSK109 genomic DNA ligation kit (Oxford Nanopore Technologies, Oxford, UK). The library was prepared according to the manufacturer’s instructions except for the following details: (1) DNA fragmentation was not performed prior to library preparation, (2) 80% ethanol was used instead of 70% ethanol, (3) the bead elution time following DNA repair and end-prep was increased from 2 to 10 min, (4) the bead elution time following adapter ligation and clean-up was increased from 10 to 15 min and carried out in a water bath set to 37 °C. Typically, between 150 and 400 ng of the prepared library quantified using a Qubit fluorometer (Thermo Fisher Scientific, Waltham, MA, USA) were used as input to a FLO-MIN106D flowcell (R9 chemistry) and run on a MinION for 48 to 72 h using default voltage settings. While most accessions were sequenced on a single flow cell, three accessions for which the initial yield was low (< 9 Gb) were sequenced a second time (using DNA from a different plant) to provide sufficient data for downstream analyses. More details regarding the Oxford Nanopore sequencing of the samples can be found in Table S9 (Additional file 1).

### Structural variation discovery from Oxford Nanopore data

Raw FAST5 sequencing files were basecalled on a GPU using Oxford Nanopore Technologies’ guppy basecaller v. 4.0.11 with parameters --flowcell FLO-MIN106 --kit SQK-LSK109. Basecalled FASTQ files obtained from a single flow cell were concatenated into a single file which was used for downstream analyses. Adapters were trimmed using Porechop v. 0.2.4 [77] with the option --discard_middle. Adapter-trimmed reads were aligned using NGMLR v. 0.2.7 [24] with the option -x ont. The resulting alignments were sorted and indexed using samtools.

At this stage, we merged the BAM files of samples that were sequenced on two different flowcells and called SVs using Sniffles v. 1.0.11 [24]. We ran Sniffles with parameters --min_support 3 (minimum number of reads supporting a variant = 3, default = 10), --min_seq_size 1000 (minimum read segment length for consideration = 1000, default = 2000), and --min_homo_af 0.7 (minimum alternate allele frequency to be considered homozygous = 0.7, default 0.8). We chose relaxed parameters compared to the defaults because our samples are inbred cultivars and heterozygosity should therefore be nearly non-existent. In order to evaluate the impact of sequencing depth on SV calling with Sniffles, we performed a subsampling analysis using some of the samples and report the detailed methods and results in Additional file 1 (Supplemental Methods and Supplemental Figures S7 to S10).

We applied a series of filters to the SVs in order to remove any spurious calls that could affect downstream analyses. Any variants called on organellar genomes or unanchored scaffolds were filtered out, along with any variants smaller than 50 nucleotides or larger than 500 kb. We only retained deletions, insertions, inversions, and duplications for further analyses, discarding unresolved breakpoints (SVTYPE=BND) as well as other complex types such as DEL/INV, DUP/INS, INVDUP, and INV/INVDUP. We removed variants called as heterozygous since heterozygous genotype calls are very likely to be spurious in these inbred lines. In order to avoid calling artificial variants in ambiguous regions of the genome (stretches of “N” due to imperfectly assembled regions of the reference genome), we also removed variants that overlapped any “N” in the reference as well as any insertion located less than 20 nucleotides away from any “N” in the reference.

The location of SVs as well as the insertion sequences reported by Sniffles are necessarily imperfect as they are based on error-prone Oxford Nanopore reads (on average 8–10% error rate based on the percent identity of our alignments). We therefore assembled a pipeline to refine the breakpoint location and the sequence content of the deletions and insertions found by Sniffles. Duplications and inversions were not considered for SV refinement because the inherent complexity of these variants made it difficult to accurately assemble them from our data. We briefly describe the pipeline here, but more details can be found in Additional file 1 (Supplemental Methods, Table S10 and Figures S26 to S28). Our breakpoint refinement pipeline starts by locally assembling all reads that were mapped by NGMLR to positions ± 200 bp from the location of the SV using wtdbg2 v. 2.5 [42]. The same reads are then aligned to the assembled sequence using minimap2 v. 2.17-r974 [43] to polish the assembly sequence using the consensus module of wtdbg2. The resulting polished assembly is subsequently aligned to the local region of the reference genome using AGE (commit 6fa60999, github.com/abyzovlab/AGE) [40]. The coordinates of the SV and insertion sequence content are then optionally updated from the information provided by the AGE alignment. When the alignment did not suggest suitable replacement coordinates or insertion content for a given SV, we simply used its representation as initially defined by Sniffles for downstream analyses instead. Following breakpoint refinement, the representation of the alleles was standardized using bcftools norm.

### Structural variant genotyping and benchmarking

We genotyped SVs on all 102 Illumina samples with Paragraph v. 2.4a [29] using three different candidate SV sets. The first candidate SV set included only variants discovered from the Illumina data and was used to assess the performance of SV discovery from Illumina data alone. The second candidate SV set included only variants discovered from Oxford Nanopore data and was similarly used to assess the performance of genotyping those variants with Illumina data. The third and last candidate SV set comprised variants discovered using both Illumina and Oxford Nanopore data and was used for the population-scale analyses on population genetics, location of variants relative to gene models, and polymorphic TEs. Despite the superior performance of long-read data for SV discovery, we decided to also include variants discovered from the Illumina data in the final SV set as they encompassed all samples.

For genotyping SVs discovered from Illumina data, the VCF files of all discovery tools (AsmVar, Manta, SvABA, smoove) were merged together using SVmerge (commit 6a18fa3d2, github.com/nhansen/SVanalyzer) [45] with parameters -maxdist 15 -reldist 0.2 -relsizediff 0.1 -relshift 0.1. Parameters were chosen in order to merge slightly differing representations of alleles that were putatively identical from a biological point of view while preserving true allele diversity at a given position.

SVs discovered from Oxford Nanopore data were also merged across samples using SVmerge with the same parameters as described above. However, for Oxford Nanopore variants, we modified SVmerge’s default behavior which selects an allele randomly from a given SV cluster. Instead, we forced the random selection to be made among the alleles that had been refined by the SV refinement pipeline, if any, to favor those alleles whose representation was hopefully closer to biological reality.

For the last candidate SV set combining Illumina and Oxford Nanopore variants, the two datasets described above were merged using SVmerge. The default behavior of SVmerge was again overridden by systematically sampling among the alleles found by Illumina whenever a SV cluster contained alleles found by both Illumina and Oxford Nanopore. Despite the greater power of Oxford Nanopore data in discovering SVs, our reasoning was that if a variant was discovered by both sequencing technologies, then the Illumina data was likely more precise given its higher basecalling accuracy. While many false positive SV calls are likely to have made their way in the candidate SV dataset, our approach has been to feed Paragraph with a candidate set as exhaustive as possible for genotyping and rely on the accuracy of its genotyping model to avoid genotyping false positive SVs. Under this assumption, the genotypes of SVs that simply do not exist in the dataset should be called as homozygous for the reference allele in all samples. Consistent with this expectation, 32.3% of the SVs in the raw output of Paragraph had an alternate allele frequency of 0.

The methods used for genotyping were identical for all three candidate SV sets. We prepared the VCF files for input to Paragraph by removing variants located less than 1 kb away from chromosome ends and padding the allele representations as required by Paragraph. We genotyped the 102 Illumina samples aligned by bwa mem following the recommendations outlined by Paragraph for population-scale genotyping, i.e., the variants were genotyped independently for each sample with multigrmpy, setting the -M option to 20 times the average sequencing depth for the sample.

We compared the genotyping results of the three genotyped datasets against the Oxford Nanopore SV set in order to assess genotyping sensitivity and precision. For this analysis, the set of variants called from the Oxford Nanopore data by Sniffles and subsequently refined was considered to be the truth. Structural variation calls made from Oxford Nanopore data may also be erroneous, especially for smaller variants [2], so this approach of treating Oxford Nanopore dataset as the ground truth is necessarily imperfect but nevertheless provides a good comparison basis for our purposes.

We compared the SV genotype calls to the ground truth set using the R package sveval v. 2.0.0 [30]. For each of the 17 samples for which Oxford Nanopore data was available, we compared the genotype calls made by Paragraph to the SVs identified in the Oxford Nanopore data for that sample. SVs genotyped as homozygous for the alternate allele by Paragraph and present in the Nanopore set were considered true positives, while SVs genotyped as homozygous for the alternate allele by Paragraph but absent from the Nanopore set were considered false positives. Note that, for benchmarking purposes, we essentially ignored heterozygous genotype calls made by Paragraph since the truth set only contained homozygous calls as expected for inbred lines. Sensitivity was defined as the ratio of the number of true positive calls to the total number of SVs in the truth set, and precision as the ratio of the number of true positive calls to the sum of true and false positive calls. We computed sample-wise precision-recall curves for various SV size classes and SV types by using a range of read count thresholds (number of reads required to support a genotype call) to filter the Paragraph genotype calls. We required sveval to explicitly compare insertion sequences by setting ins.seq.comp = TRUE, but we otherwise used default settings. We extended sveval’s functionality by also assessing duplications under the same overlap conditions as the package already provides for deletions and inversions. Benchmarks were performed both on the complete set of SVs and on a subset of SVs located in non-repeat regions in order to assess the contribution of repeats to genotyping errors. A SV was defined as belonging to a repetitive region if it had a 20% or higher overlap to regions in the repeat annotation for the Williams82 assembly version 4 retrieved from Phytozome [78].

For the SVs discovered by Illumina, we computed additional precision-recall curves by filtering the SVs in the dataset genotyped by Paragraph based on two different metrics of SV quality: (1) the number of times the alternate allele is observed in homozygous genotype calls across the whole population (referred to hereafter as the homozygous ALT count) and (2) the number of calling tools (out of a maximum of four) that originally reported the SV. The more stringent homozygous ALT count was used instead of alternate allele frequency as a measure of the frequency of the SV in the population since true SVs are expected to be homozygous for the alternate allele in these inbred lines. Note that both of these quality measures (homozygous ALT count and the number of tools supporting an SV) effectively filter SV records and not individual genotype calls. The objective of these analyses was to see whether filtering on SV frequency or calling-tool support for variants could result in a higher-quality dataset.

### Population genetics analyses

We used the set of merged Illumina and Oxford Nanopore SVs genotyped by Paragraph to evaluate whether SV calls could replicate population genetics analyses (structure, PCA, and phylogenetic tree) made from SNV calls. We applied methods similar to Torkamaneh et al. [6] in order to compute population structure for the 102-sample population. We called SNVs using Platypus v. 0.8.1.1 [52] with parameters --minMapQual=20 --minBaseQual=20 --maxVariants=10 --filterReadsWithUnmappedMates=0 --filterReadsWithDistantMates=0 --filterReadPairsWithSmallInserts=0. We filtered Platypus calls to keep only biallelic SNVs located on any of the 20 reference chromosomes. We only retained SNVs with a minor allele frequency ≥ 0.05, proportion of missing sites ≤ 0.4, and heterozygosity rate ≤ 0.1. The resulting 1.27 M SNVs were converted to PLINK BED format [53] and used as input to fastStructure v. 1.0 [79] using *k* = 5 as determined by Torkamaneh et al. [6]. A PCA was also computed on those SNVs using PLINK v1.90b5.3 with default parameters. Finally, we used the same SNV dataset to build a neighbor-joining phylogenetic tree with the PHYLIP package v. 3.697 [80] using pairwise distances computed with PLINK. We computed support for each node in the tree using a bootstrapping approach by sampling with replacement from the VCF file of SNVs 100 times and computing a new neighbor-joining tree from each such sample. The number of bootstrap trees supporting each node was then assessed using the prop.clades function the ape R package v. 5.4.1 [81].

The same population genetics analyses were also performed on the population-scale dataset of Illumina/Oxford Nanopore SVs genotyped with Paragraph. For these analyses, we filtered SV genotype calls by setting those with less than two supporting reads to missing. We also removed duplications, inversions, and records with a homozygous ALT count < 4 or a proportion of missing sites ≥ 0.4. The resulting filtered SV dataset was used to compute population structure with fastStructure, PCA with PLINK, and neighbor-joining tree with PLINK and PHYLIP using the same methods as described for SNVs. To test for correlation between the pairwise distance matrices computed from SNVs and SVs, we performed a two-sided Mantel test with 999 permutations using the mantel.test function of the ape R package.

Since the statistical methods implemented by the programs used above do not depend on an evolutionary model of changes in DNA sequence but rather simply on genotype calls, these methods could be applied to the SV dataset in exactly the same way as for SNVs.

### Potential impact on genes

We annotated deletions and insertions based on their overlap with various gene features. We retrieved the positions of the gene models for Williams82 assembly 4 from Phytozome [78] and determined for each SV whether it overlapped any of the following genic features: coding sequences, non-coding genic sequences, and regions 5 kb upstream of genes. These categories were mutually exclusive, such that an SV overlapping both coding and non-coding sequences was only labeled as “coding sequences.” Similarly, an SV was only labeled as “5 kb upstream” if it did not overlap any genic sequences. The SVs that overlapped none of the features described above were labeled as “intergenic.”

We first used these annotations to assess whether SVs were over- or underrepresented within particular genic features by comparing the observed proportions of deletions and insertions overlapping each feature to what would be expected by chance. We used three different measures of random expectation of the proportion of SVs overlapping genic features. The first measure was a naive comparison to the proportion of the genome corresponding to each genic feature. This comparison is however biased because repetitive regions (which are largely non-genic) are less effectively queried for SVs than non-repetitive genic regions. Therefore, we also replicated the analysis by excluding repeated regions, which provided a second measure of random expectation. Finally, we performed a randomization test by estimating the distribution over the proportions of SVs that would be expected to overlap each genic feature by random chance. This was done by shuffling the start positions of SVs within the 100-kb genome-tiling bins in which they are located 5000 times and annotating them with the genic features overlapped. We used 100-kb bins tiled along the whole genome instead of shuffling the positions genome-wide to take into consideration the heterogeneity of the genome while allowing SVs to be repositioned in a gene-agnostic manner.

We also used the genic feature annotations to study differences in mean alternate allele frequencies of SVs depending on the features they overlapped. We averaged the frequencies of insertions and deletions overlapping each of the four genic features and computed the difference between the mean SV frequencies for each of the six possible pairwise combinations of features. SVs with a frequency of 1 in the population were excluded from this analysis because they might be due to errors in the reference assembly. Statistical significance was assessed using a randomization test by shuffling the genic feature annotations 10,000 times to get a distribution of mean SV frequency differences between feature groups under a random scenario. We computed one-sided *p*-values by comparing the observed values to the random distributions thus generated, using a significance threshold of *α* = 0.05 / 6 = 0.0083 to compensate for multiple testing.

Finally, we carried out enrichment analyses of GO [82] Biological Process terms and PFAM domains [83] to assess whether high-frequency gene-impacting SVs were associated with particular biological functions. We identified insertions and deletions with an alternate allele frequency ≥ 0.5 and < 1 among those overlapping coding sequences and found 546 genes overlapped by such SVs. These genes constituted our gene set of interest for the enrichment analyses. We used the GOstats Bioconductor package v. 2.56.0 [54] along with GO and PFAM annotations for Williams82 assembly version 4 retrieved from Soybase on April 20, 2021, to test this gene set for over- and underrepresentation of particular GO Biological Process terms or PFAM protein domains. We only tested GO terms and PFAM domains that were represented by at least 20 and 10 genes, respectively. For the GO terms, we used the conditional test as implemented in GOstats and the GO.db annotation package v. 3.12.1 [84]. We applied a Bonferroni correction to the *p*-values of both the GO and PFAM enrichment tests by multiplying the *p*-values by the number of terms/domains tested.

### Transposable elements

We annotated TEs in the SVs discovered using the SoyTEdb database [48] downloaded from SoyBase [66]. We queried the deleted or inserted sequences of all deletions and insertions ≥ 100 bp against SoyTEdb using blastn v. 2.11.0+ [85] with default parameters. Any queried sequence that aligned to a TE in the database with at least 80% of the query length and 80% of the length of the TE sequence was considered a match and annotated accordingly with the classification of the best-matching TE. All alignments that matched these criteria had an extremely small E-value (< 10^−80^) and therefore no additional filtering on this was needed.

The annotated SVs were then used to determine both the proportion of polymorphic TEs belonging to each category and the physical location of polymorphic TEs in the genome. We also computed the proportions of TEs ≥ 100 bp in each category within the reference repeat annotation from Phytozome and compared those to the estimated proportions in the SV dataset. The estimated number of polymorphic TEs within various LTR retrotransposon families and DNA TE types were also compared to the number of non-reference TEs found by Tian et al. [38] to check whether our results were consistent with previous reports.

Soybean DNA TEs have received little attention compared to retrotransposons, which are more prevalent and polymorphic in this species (e.g., [38, 86]). DNA TEs that have TIR typically transpose using a “cut and paste” mechanism. This mechanism generates a TSD upon insertion into the genome and leaves this TSD as well as possible additional nucleotides upon excision due to DNA repair [87]. In order to study the dynamics of polymorphic DNA TEs within our population, we devised a pipeline based on local assembly and multiple sequence alignment of the DNA TE insertions. Briefly, the pipeline locally assembles Oxford Nanopore reads surrounding the sites of polymorphic DNA TEs for all samples using wtdbg2 and aligns these assemblies to each other using MAFFT v. 7.475 [50] before identifying TIR and TSD sequences with Generic Repeat Finder v. 1.0 [51]. For more details on the pipeline, see Supplemental Methods (Additional file 1). Our goal with this pipeline was to determine whether the insertion/deletion polymorphisms at various sites were due to novel TE insertion, TE excision, or a combination of both phenomena. We applied this pipeline to SVs that were annotated as TIR DNA TEs and whose matching sequence in the SoyTEdb database was matched by at least three SVs. We limited ourselves to TE sequences that were matched by at least three SV events under the assumption that TEs present in multiple copies were more likely to have been recently active. For insertions that had both TIR and TSD sequences unambiguously identified, we computed the proportion of matching nucleotides in the alignment of the two terminal repeats and averaged the values across all local assemblies bearing the insertion in order to get a single value for that SV.

### Software used

Unless otherwise stated, all statistical analyses and data manipulation were conducted in R version 3.5.0 or 4.0.3 [88] and Bioconductor version 3.08 or 3.12 [89]. Analyses made use of Bioconductor packages Biostrings v. 2.58.0 [49], GenomicRanges v. 1.42.0 [44], Rsamtools v. 2.6.0 [90], rtracklayer v. 1.50.0 [91], and VariantAnnotation v. 1.36.0 [92]. All scripts used for the analyses described in this paper are available on GitHub [93].

## Supplementary Information

**Additional file 1.** Supplemental methods, supplemental tables S1 to S10 and supplemental figures S1 to S28.

**Additional file 2. **Statistics of the conditional hypergeometric test for the overrepresentation of GO Biological Process terms using the GOstats R package. *P*-values are Bonferroni-corrected *p*-values.

**Additional file 3. **Statistics of the conditional hypergeometric test for the underrepresentation of GO Biological Process terms using the GOstats R package. *P*-values are Bonferroni-corrected *p*-values.

**Additional file 4. **Statistics of the hypergeometric test for the overrepresentation of PFAM domains using the GOstats R package. *P*-values are Bonferroni-corrected *p*-values.

**Additional file 5. **Statistics of the hypergeometric test for the underrepresentation of PFAM domains using the GOstats R package. *P*-values are Bonferroni-corrected *p*-values.

**Additional file 6.** SVs identified as polymorphic transposable elements among the dataset of combined Illumina/Oxford Nanopore variants genotyped with Paragraph. Positions of the SVs and metadata about their best blastn match in the SoyTEdb database are described.

**Additional file 7.** Proportion of matching nucleotides in TIR of SVs for which intact TSD sequences and matching TIR were identified with GenericRepeatFinder.

**Additional file 8. **Multiple alignment of the Williams82 assembly version 4 reference sequence and local *de novo* assemblies of 7 samples at the site of a 480-bp Stowaway MITE insertion (Gm04:2,257,090). Samples OAC Petrel and Roland bear the 480-bp insertion, while Alta bears the 6-bp TACGAG insertion; other samples match the reference sequence. Asterisks mark the locations of the TSD sequences in samples OAC Petrel and Roland.

## Data Availability

All plant materials used in this study were collected in compliance with institutional, national, and international guidelines and legislation. All data generated or analyzed during this study are included in this published article, its supplementary information files and publicly available repositories. The Illumina sequencing data used in this study is available on the SRA repository through BioProject accession number PRJNA356132 [62]. The Oxford Nanopore sequencing data generated during the current study is available in the SRA repository through BioProject accession number PRJNA751911 [94]. The SoyTEdb database is available on SoyBase [95]. Gene Ontology annotations for Williams82 assembly version 4 [96] as well as chloroplast and mitochondrion genome sequences [97] are also available on SoyBase. The non-reference transposable elements found by Tian et al. can be downloaded from the supplementary data to their paper [38]. The reference genome sequence and annotation of soybean cultivar Williams82, assembly version 4, are available on Phytozome [98]. VCF files generated during this study and results of the permutation test for the analysis of the proportion of SVs overlapping various genic features are available on the figshare repository [99]. The code for all the analyses described in the paper [93] and the breakpoint refinement pipeline [100] are available on GitHub. Versions of the code at the time of submission are archived on the figshare repository for the breakpoint refinement pipeline [101] and the analysis code [102].

## Abbreviations

GO: Gene ontology
MITE: Miniature inverted-repeat transposable element
PCA: Principal component analysis
SRA: Sequence read archive
SV: Structural variant
SNV: Single-nucleotide variant
TE: Transposable element
TIR: Terminal inverted repeat
TSD: Target site duplication
