# Supplementary material for: Combined use of Oxford Nanopore and Illumina sequencing yields insights into soybean structural variation biology
Source: BMC Biol. 2022 Feb 23;20:53. doi: 10.1186/s12915-022-01255-w (PMC8867729; doi:10.1186/s12915-022-01255-w)
Supplement: Supplementary file 1 — Additional file 1. Supplemental methods, supplemental tables S1 to S10 and supplemental figures S1 to S28. [file 12915_2022_1255_MOESM1_ESM.pdf]

Supplemental data to "Combined use of Oxford Nanopore and Illumina sequencing yields insights into soybean structural variation biology"

Marc-André Lemay   Jonas A. Sibbesen   Davoud Torkamaneh
Jérémie Hamel   Roger C. Levesque   François Belzile

Supplemental methods

1 Description and testing of the SV refinement pipeline **4**
1.1 Details of the steps in the pipeline 4
1.2 Parameters determining whether SVs are updated or not 6
1.3 Testing the effect of refined SVs on genotyping performance 6
1.3.1 Application of the pipeline 6
1.3.2 Input SV datasets 7
1.3.3 Genotyping methods 7
1.3.4 Benchmarks: raw versus refined variants 8
1.4 Conclusion on the SV refinement pipeline 9
1.5 Software availability 9

2 Detailed methods on the DNA transposable element analysis **10**
2.1 Description of the analysis pipeline 10
2.2 Detailed analysis of a 480-bp Stowaway MITE insertion 11

3 Subsampling analysis of four samples sequenced using Oxford Nanopore sequencing **14**

Supplemental tables

Table S1 Number of SVs per Oxford Nanopore sample 17
Table S2 Number of deletions and insertions overlapping various genic features and proportion of these features within the reference annotation of Williams82 18

Table S3 Randomization test on the mean allele frequencies of SVs depending on the genic features overlapped 19
Table S4 List of Gene Ontology Biological Process terms that are overrepresented ($\alpha = 0.05$) among coding sequences impacted by SVs with frequency > 0.5 20
Table S5 List of Gene Ontology Biological Process terms that are underrepresented ($\alpha = 0.05$) among coding sequences impacted by SVs with frequency > 0.5 21
Table S6 List of PFAM domains that are overrepresented ($\alpha = 0.05$) among coding sequences impacted by SVs with frequency > 0.5 22
Table S7 Metadata on the Illumina sequencing data of the 102 Canadian accessions 23
Table S8 DNA extraction and preparation methods for Oxford Nanopore sequencing 27
Table S9 Metadata on Oxford Nanopore sequencing runs 28
Table S10 Classification of deletions and insertions for four samples used to test the SV refinement pipeline 29

Supplemental figures

Figure S1 Graphical summary of the methods used in this paper 31
Figure S2 Heterozygosity rates of the population of 102 Canadian soybean cultivars 32
Figure S3 Sensitivity and precision of Illumina deletion and insertion genotyping in non-repeat regions 33
Figure S4 Sensitivity and precision of Illumina duplication and inversion genotyping 34
Figure S5 Sensitivity and precision of Illumina duplication and inversion genotyping in non-repeat regions 35
Figure S6 Relationship between Oxford Nanopore sequencing depth and genotyping precision of Illumina SVs 36
Figure S7 Effects of sequencing depth on the sensitivity and precision of deletion discovery from Oxford Nanopore sequencing data 37
Figure S8 Effects of sequencing depth on the sensitivity and precision of insertion discovery from Oxford Nanopore sequencing data 38
Figure S9 Effects of sequencing depth on the sensitivity and precision of duplication discovery from Oxford Nanopore sequencing data 39

Figure S10 Effects of sequencing depth on the sensitivity and precision of inversion discovery from Oxford Nanopore sequencing data 40
Figure S11 Effect of homozygous ALT count on genotyping performance of Illumina deletions and insertions 41
Figure S12 Effect of homozygous ALT count on genotyping performance of Illumina duplications and inversions 42
Figure S13 Effect of the number of supporting calling tools on genotyping performance of Illumina deletions and insertions 43
Figure S14 Size distribution of the SVs reported by various calling tools using Illumina data 44
Figure S15 Number of true positive and false positive deletion calls reported by various calling tool combinations 45
Figure S16 Number of true positive and false positive insertion calls reported by various calling tool combinations 46
Figure S17 Relationship between Oxford Nanopore read N50 and genotyping precision of Oxford Nanopore insertions 47
Figure S18 Sensitivity and precision of Oxford Nanopore deletion and insertion genotyping in non-repeat regions 48
Figure S19 Sensitivity and precision of Oxford Nanopore duplication and inversion genotyping 49
Figure S20 Sensitivity and precision of Oxford Nanopore duplication and inversion genotyping in non-repeat regions 50
Figure S21 Sensitivity and precision of joint Illumina/Oxford Nanopore deletion and insertion genotyping 51
Figure S22 Sensitivity and precision of joint Illumina/Oxford Nanopore deletion and insertion genotyping in non-repeat regions 52
Figure S23 Principal component analyses (PCA) of the whole Canadian soybean panel 53
Figure S24 Neighbour-joining phylogenetic trees of the population of 102 Canadian soybean cultivars 54
Figure S25 IGV screenshot of Oxford Nanopore and Illumina read alignments of three samples at the location of a polymorphic Stowaway TE insertion 55
Figure S26 Genotyping sensitivity of raw and refined Oxford Nanopore SVs for four samples using BayesTyper 56
Figure S27 Genotyping sensitivity of raw and refined Oxford Nanopore SVs for four samples using vg 57
Figure S28 Genotyping sensitivity of raw and refined Oxford Nanopore SVs for four samples using Paragraph 58

1 Description and testing of the SV refinement pipeline

One of the main objectives of this project is to use Illumina data to genotype SVs that were originally discovered using Oxford Nanopore sequencing data. This would enable the use of Illumina data to carry out population-scale genotyping of variants that were initially discovered in a smaller set of samples sequenced with long-read technologies.

Oxford Nanopore data, however, has a high error rate. This makes it challenging to accurately describe breakpoints and sequence content (for insertions) and may negatively impact our ability to genotype the variants discovered with this technology using short reads.

In order to address this issue, we assembled a pipeline that can be used to refine the SVs discovered by Sniffles [24]. By refinement, we mean that the position of the breakpoints (start and end position for deletions, and single location for insertions) and the sequence content (for insertions) can be updated to improve the quality of the variants. The current version of the pipeline ignores SVs other than deletions or insertions due to difficulties in assembling and aligning them properly.

The pipeline is based purely on Oxford Nanopore data (i.e. it does not need Illumina data e.g. for polishing or error correction) and therefore could potentially be used for SV refinement with any Oxford Nanopore or even PacBio dataset.

1.1 Details of the steps in the pipeline

The pipeline performs a local assembly of the Oxford Nanopore reads aligned to the region of interest and then uses AGE [40] to align this assembly to the appropriate region of the reference genome. This alignment is then used to update the breakpoints and inserted sequence of the variant if certain quality thresholds are met.

This alignment pipeline is largely inspired by the guidelines suggested by the authors of AGE in their newer LongAGE paper [41] and the authors of wtdbg2 [42] and consists of the following steps:

1. For every insertion or deletion, Oxford Nanopore reads overlapping the region of interest are extracted from the NGMLR [24] BAM file. The region of interest is a window spanning ± 200 nucleotides from the SV breakpoints (start and end of the breakpoint for deletions, and single location for insertions).
2. The reads extracted in this way are assembled using wtdbg2. We use preset values for Oxford Nanopore sequencing (`-x ont`) except for the following parameters: `-S 1` (no subsampling of reads), `-e 2` (minimum read coverage of 2 for an edge in the graph), `-L 1000` (minimum read size of 1 kb), `-g 500000` (expected genome size of 500 kb).

3. A consensus sequence for the assembly is produced using the `wtpoa-cns` command from wtdbg2 with default parameters.

4. This assembly is polished using Oxford Nanopore data. For this, the same reads that were used for assembly are aligned to the consensus sequence using minimap2 [43] with options `-ax map-ont -r2k`. These aligned reads are used to update the consensus sequence with `wtpoa-cns`.

5. Following assembly and polishing, the reference sequence is extracted $\pm$ 500 nucleotides from the SV breakpoints for the assembled contig to be aligned against it.

6. The largest of the assembled contigs (there is usually only one) is then aligned to the reference sequence using AGE with parameters `-allpos`, `-go=-1` (gap opening penalty of -1 to account for the high indel error rate of Oxford Nanopore data), `-indel` (the expected excised region is an indel) and `-both` (try alignment of both the sequence and its reverse complement, and report the best alignment).

Once the alignment is completed, the results are parsed and used to extract the location of the regions excised by AGE from both the assembled contig and the reference genome. In the case of a deletion, we expect a fragment of the reference genome to be excised, whereas in the case of a insertion, we expect a fragment of the assembled contig to be excised. From this information, the location of the excised fragment is transformed to reference-based coordinates and the inserted sequence is extracted from the assembled contig (using the reverse complement if applicable). The following metrics are then computed in order to compare the (refined) deletion/insertion suggested by the AGE alignment to the original (raw) one defined by Sniffles:

- For deletions, the reciprocal overlap between the raw deletion and its refined counterpart. The reciprocal overlap is the minimum of the two following values: proportion of deletion 1 covered by deletion 2, and proportion of deletion 2 covered by deletion 1. This overlap is computed using functions from the GenomicRanges R package [44].

- For insertions, the offset of the two insertions, i.e. the (absolute) physical distance in base pairs between the positions of the raw and refined insertion.

- For insertions, the Levenshtein (edit) distance between the two inserted sequences, as computed by the R function `adist`. From this distance, a *distance ratio* is computed, which corresponds to the Levensthein distance between the two insertions divided by the length of the longest insertion. This provides a relative measure of the difference in sequence content between the two insertions.

- In addition to the metrics related to the excised fragments themselves, the percentage of nucleotides that share identity and the percentage of nucleotides affected by gaps in the aligned flanking regions are also extracted from the output of AGE.

Note that SVs longer than 50 kb are excluded from the pipeline because of the high memory and computing time requirements of aligning to those variants using AGE. Although a newer version of AGE called LongAGE [41] requiring less memory has been recently released, we found it unsuitable for our needs due to cryptic error messages and slower speed.

1.2 Parameters determining whether SVs are updated or not

Our objective is to update the position of the breakpoints and the sequence content such that the SVs updated in this way are closer to the actual sequence, and thus both easier to genotype from Illumina data and more representative of reality. However, we must also avoid updating the variant in a way that is inaccurate (as would occur if, for example, AGE identified a deletion or insertion different from the target one). Therefore, we established conditions to decide whether or not an SV should be updated. Based on analyses carried out during the development of the pipeline, we have settled on the following thresholds to decide whether a variant should be updated:

- Reciprocal overlap ≥ 0.5 (for deletions)
- Offset ≤ 50 (for insertions)
- Edit distance ratio ≤ 0.5 (for insertions)
- Percent identity of the alignment ≥ 85
- Percentage of gaps in the assembly ≤ 15

1.3 Testing the effect of refined SVs on genotyping performance

The main objective of the SV refinement pipeline described above is to improve the genotyping of SVs with Illumina data. In order to verify whether the pipeline indeed improved genotyping, we compared the genotyping precision and sensitivity of raw and refined SVs using three different genotypers: BayesTyper v. 1.5 [31], vg v. 1.23.0 [30], and Paragraph v. 2.4a [29].

1.3.1 Application of the pipeline

For these testing purposes, we applied the SV refinement pipeline described above to the first four samples for which we obtained Oxford Nanopore data. SVs discovered using Sniffles were filtered as described in the main text (notably, only SVs genotyped as homozygous were kept) and then used as input to the pipeline. Insertions and deletions were refined if they met the required thresholds, which were as described above except for the minimum

reciprocal overlap which was set to 0.7 and the maximum distance ratio which was set to 0.45.

Table S10 shows the number of variants that could be refined or not for each sample. For all four samples, a majority of SVs subjected to the pipeline met the conditions for refinement.

There can be three main reasons why a variant is not refined:

- The proposed refined variant did not meet one or several quality thresholds when compared to the raw variant.
- The size of the variant was > 50 kb.
- No assembly or alignment was produced by the pipeline.

In either of these cases, we simply keep the original representation of the SV as called by Sniffles.

1.3.2 Input SV datasets

In order to assess the effect of refining SVs on genotpying performance, we generated an input dataset of raw variants and another input dataset of refined variants.

In order to generate input datasets for genotyping, the SV sets obtained from the four samples were merged using SVmerge [45] with parameters `-maxdist 15 -reldist 0.1 -relsizediff 0.1 -relshift 0.1 -seqspecific`. For refined variants, the default random selection behavior of SVmerge when combining several variants together was modified to systematically favor variants that had been updated by the pipeline.

Following this, the VCF files were coordinate-sorted, normalized (using `bcftools norm`, [46]) and duplicate records were removed.

1.3.3 Genotyping methods

The raw and refined SV datasets were both used as input to three genotyping programs, BayesTyper, vg and Paragraph.

BayesTyper We followed the methods suggested by the authors of BayesTyper for genotyping (`https://github.com/bioinformatics-centre/BayesTyper`).

Briefly, k-mers were counted from the adapter-trimmed fastq files using KMC [47] and a Bloom filter was applied using the `bayesTyperTools makeBloom` utility.

In parallel, `BayesTyperTools combine` had to be run to make the VCF input files suitable for input to BayesTyper.

Genotyping was then carried out using `bayesTyper cluster` and `bayesTyper genotype` sequentially with default parameters except for the use of the mitochondrial and chloroplastic genome sequences as decoy sequences.

Precision-recall curves for BayesTyper were generated by varying the genotype quality (GQ) threshold necessary to make a call.

vg Variation graphs were created from the VCF files using `vg construct` and then converted to PackedGraph format using `vg convert -p`. The graphs were then indexed prior to mapping using `vg index -L -x` to generate the xg index and `vg index -g` to generate the GCSA index. GCSA indexing was carried out on a version of the graph that had been pruned with `vg prune`.

Reads were mapped separately for each sample using `vg map`. These mappings were then used to call the SVs explicitly defined in the input VCF files using `vg pack -Q5` and `vg call -v`.

Precision-recall curves for vg were generated by varying the genotype quality (GQ) threshold necessary to make a call.

Paragraph VCF input files were prepared for input to Paragraph using similar methods to those described in the main text, i.e. variants located less than 500 nucleotides away from chromosome ends were removed and variants were padded with reference nucleotides where required. Samples were genotyped separately using the `multigrmpy` utility of Paragraph.

Precision-recall curves for Paragraph were generated by varying the minimum number of reads (DP) threshold necessary to make a call.

1.3.4 Benchmarks: raw versus refined variants

Benchmarks were carried out using the sveval R package [30] following the methods described in the main text.

Bayestyper Figure S26 shows the benchmarking results obtained for BayesTyper. Overall, the refined deletions resulted in a higher sensitivity but similar precision. The benefits of SV refinement were obvious for larger deletions, whereas results were sometimes worse for deletions in the range of 100-1000 nucleotides. The improvemement brought by refinement is more obvious for insertions than it is for deletions, most likely because the refinement pipeline also improved the sequence content of the insertions. The poor overall sensitivity of the

results obtained with BayesTyper when genotyping Oxford Nanopore SVs is probably due to the small tolerance of this program to errors in SV breakpoint location or sequence content, as has been previously documented [30]. Nevertheless, the improvement in sensitivity obtained by using refined SVs suggests that the SV refinement pipeline indeed improves the quality of the variants.

vg Figure S27 shows the benchmarking results obtained for vg. Refining the deletions does seem to have improved the results, although only very slightly. For insertions, however, the improvement resulting from refinement is obvious, both in terms of sensitivity and precision. Again, this suggests that the breakpoint refinement pipeline improved the accuracy of the sequence content of insertions, such that they could be more accurately genotyped by vg.

Paragraph Figure S28 shows the benchmarking results obtained for Paragraph. Using refined variants results in a slight increase in sensitivity for Paragraph, both for deletions and smaller insertions ($<$ 1,000 bp). The apparent drop in precision for insertions $\geq$ 1,000 bp is likely due to the fact that the Oxford Nanopore data represented a poor ground truth dataset for these samples in this size range, as explained in the main text. It is therefore expected that an increase in sensitivity from refining the variants will result in an apparent loss of precision in this size range.

1.4 Conclusion on the SV refinement pipeline

Overall, the results show that the breakpoint refinement pipeline results in higher genotyping sensitivity than using the raw variants as called by Sniffles. While the results are not immediately obvious when using Paragraph, the improvements obtained using BayesTyper and vg suggest that the quality of the SVs is indeed higher following breakpoint refinement. This is probably due to the high tolerance of Paragraph to errors in SV sequence (as documented in [29]) compared to the two other tools. However, even if the improvement in genotyping performance using Paragraph is not obvious, downstream analyses should still benefit from more accurate SV breakpoints and sequence.

1.5 Software availability

The SV refinement pipeline described here is available from `https://github.com/malemay/breakpoint_refinement`.

2 Detailed methods on the DNA transposable element analysis

2.1 Description of the analysis pipeline

To study potential DNA transposable element (TE) activity in more detail, we extracted the SVs corresponding to all DNA TEs in the SoyTEdb database [48] that were matched by at least 3 SVs. This represented 51 TEs in the database and 237 individual SVs.

For each of these SVs, we extracted the Oxford Nanopore reads at the location of the SV and assembled them individually for each sample following methods similar to those used for the breakpoint refinement pipeline. Briefly, reads overlapping positions ± 200 bp from the SV were used for assembly with wtdbg2 and polishing with wtpoa_cns after re-aligning the reads to the assembly with minimap2.

The assemblies were then used for multiple alignment across samples in order to identify TE boundaries as well as target site duplication (TSD) and terminal inverted repeat (TIR) sequences. We used the `PairwiseAlignment` function from the Bioconductor Biostrings package [49] to individually align all assemblies to a section of the reference genome ± 500 bp from the putative TE insertion site. This pairwise alignment was only done in order to extract the part of the assemblies that was of interest.

We then used the aligned sequences obtained from the pairwise alignment as input for multiple alignment. Multiple alignment was done with the MAFFT [50] suite of tools using the command `ginsi --reorder` which performs multiple global alignment.

We processed multiple alignments so as to only keep high-quality alignments that showed obvious TE insertion polymorphism. First, we manually checked all alignments and only kept those that showed an insertion/deletion polymorphism of the expected length. Second, for a given multiple alignment, several of the sequences often showed very poor similarity to the reference genome, possibly because of issues with their assembly. We removed such sequences by filtering out those that had less than 75% percent identity with the reference in the first and last 500 bp of the alignment (the nucleotides flanking the insertion/deletion site). Typically, only short TEs (< 500 bp, essentially MITEs) remained following these filtering steps because the longer ones were too challenging to assemble and align properly.

A total of 72 SV events representing 25 individual entries in the TE database remained following filtering. The sequences that remained following these filtering steps typically differed only in the presence or absence of the TE sequence. Sequences that contained the TE sequence could be analyzed further to identify TSD and TIR sequences from the TE insertion sequence. 70 out of the 72 SVs had at least one sequence bearing the insertion after filtering. In total, 301 sequences bearing the insertion could be queried for TSD and TIR sequences. 31 of these 301 were extracted from insertions that are present in the reference, whereas the 270 remaining insertion sequences were extracted from the local assemblies of

the Oxford Nanopore reads.

We analyzed the insertion sequences using the GenericRepeatFinder software [51] with the following command:

```
grf-main -i input_fasta -o .  -c 1 -p 30 -s 10 --seed_mismatch 8 --min_tr 12
```

The parameters `--min_space` and `--max_space` were adjusted to be -60 and +20 relative to the expected SV size, respectively. The minimum (`--min_tsd`) and maximum (`--max_tsd`) TSD length were set to 2 for the DTT superfamily and 3 for the DTH superfamily, while these values were 5 and 10, respectively, for the DTM superfamily. We used relatively relaxed parameters for seed matching because the presence of TSD and TIR sequences was expected and we wanted to maximize the probability of finding them.

We parsed the output of GenericRepeatFinder to get the location and sequences of the TSD and TIR. In many cases, several potential TSD/TIR sequences could be identified from a given input sequence. For DTT TEs, only entries with a TSD matching the expected TA sequence were kept. For DTH TEs, only entries with a TSD matching either TAA or TTA were kept. Despite those filters, several TSD/TIR candidates often remained for a given sample. Therefore, the candidates were ranked following an automated procedure according to their proximity to the expected insertion location and similarity to expected insertion size. For TEs in the DTM family, the highest priority was given to longer TSDs. For every sample, we considered the TSD/TIR entry with the highest priority as the only match for that sample.

We annotated the multiple alignments with the putative TSD/TIR sequences and visually checked for consistency between these sequences and the location of the insertion. Our analysis relied entirely on the automatically identified TSD/TIR sequences; samples for which the best matching TSD/TIR sequence was not consistent were discarded and were not analyzed further. In total, we identified high-quality TSD/TIR sequences for 42 individual SVs. For each of the TIR sequences identified, we computed the percentage of matching nucleotides between the two inverted repeats and averaged them over all insertion sequences for a given SV.

Some of the TIR sequences were identified directly from the reference sequence and may thus have been of higher quality than the error-prone assemblies generated from the Oxford Nanopore data. Therefore, we only show the results obtained from the de novo-assembled sequences in figure 6c of the main text. This results in 40 individual SVs collectively representing 17 sequences in the SoyTEdb database.

2.2 Detailed analysis of a 480-bp Stowaway MITE insertion

A closer look at the multiple alignments revealed that in almost all cases, the sequences without the insertion presented a single occurence of the TSD sequence. This observation is consistent with a scenario where the TE never inserted into the sequence, instead of having

excised from it. In a single case however, that of a 480-bp insertion of a Tc1-Mariner (DTT) element (actually a Stowaway MITE) at position 2,257,090 of chromosome Gm04, visual analysis of the multiple alignment revealed that three different alleles are segregating at the insertion site:

- The reference allele (no insertion at the target position)
- A 480-bp insertion that corresponds to the TE insertion
- A 6-bp insertion of nucleotides TACGAG

We speculate that this last allele results from the excision of the TE insertion. Indeed, the TA part of the insertion corresponds to the expected TSD sequence for a Tc1-Mariner element. The remaining CGAG nucleotides would likely have been inserted during the repair of the DNA break following excision of the TE. Interestingly, this insertion is by far the one for which the similarity between the two TIR sequences was highest among the ones studied, at 96.3%.

To investigate this question further, we analysed the SNV patterns surrounding the insertion site. If the TACGAG indeed resulted from the excision of the TE, then the haplotypes corresponding to the TE insertion and the 6-bp insertion should be more similar to each other than to the reference sequence, as the excision would have occurred within the TE insertion genetic background.

The SNVs with FILTER = PASS among those discovered by Platypus [52] were extracted from the VCF file of the 102 samples sequenced by Illumina to perform this analysis. The analysis of linkage disequilibrium (LD) using PLINK [53] in a 200-kb window surrounding the insertion location at Gm04:2,257,090 revealed a high-LD block spanning positions 2,220,398 to 2,259,326 that included the location of the insertion. The SNVs in that 39-kb window were therefore extracted from the Platypus VCF file and used for downstream analyses.

By combining the information obtained from Platypus and SV genotyping with Paragraph, we were able to classify samples into three groups depending on their status at the TE insertion locus:

- absent: Absence of the TE (reference allele)
- present: Presence of the TE insertion (as genotyped by Paragraph)
- excised: Presence of the 6-bp insertion putatively left by TE excision (as genotyped by Platypus)

The number of samples in each group was as follows:

- absent: 71

- present: 9
- excised: 14

All genotype calls for the TE insertion were homozygous and were therefore easy to classify as "present" when homozygous for the alternate allele. For the 6-bp insertion, however, some genotype calls were heterozygous. In all such cases, visual analysis of the aligned Illumina reads confirmed that these were most likely mis-genotyped and were actually homozygous for the alternate allele. Therefore, samples that were heterozygous at that site were classified as "excised" in addition to those that were homozygous for the alternate allele. Samples that were called as homozygous for the reference allele at the 6-bp insertion position were classified as "absent". Finally, eight (8) samples for which missing genotype calls did not allow unambiguous classification were discarded and not used for further analysis.

Using this classification, we computed the alternate allele frequency within each of the three allele classes of the 156 SNVs in the 39-kb LD block. These results are visualized in figure 6d of the main text. They show clear similarity between the "present" and "excised" haplotypes, which is indeed consistent with the 6-bp insertion being a remnant from the excision of the 480-bp TE.

3 Subsampling analysis of four samples sequenced using Oxford Nanopore sequencing

To investigate the effect of the relatively modest average sequencing depth (12X) of the Oxford Nanopore data used in this study, we performed a subsampling analysis of the data for four cultivars.

We adopted a subsampling approach in order to simulate lower coverage from the four cultivars that were sequenced with > 16X sequencing depth (Alta, Maple Isle, AC2001, OAC Lakeview). For each of the cultivars, we sampled 20%, 40%, 60% and 80% of the aligned reads using `samtools view --subsample` [46]. Five (5) replicates of each cultivar/sampling fraction combination were generated using a different randomly chosen seed for each sample. Each of the 80 subsampled `.bam` files were processed to generate a catalog of SV calls from each of them and benchmark their results using the same methods as described in the main text:

- SV calling with Sniffles using parameters `--min_support 3 --min_seq_size 1000 --min_homo_af 0.7`
- Filtering of variants by removing those meeting the following conditions:
  - Variants other than insertions, deletions, duplications and inversions
  - Variants called as heterozygous
  - Variants overlapping any `N` in the reference sequence or containing any `N` in their sequence
  - Variants located on unanchored scaffolds or organellar genomes
  - Insertions located less than 20 bp away form any `N` in the reference sequence
  - Variants smaller than 50 bp or larger than 500 kb
- Benchmarking of each such filtered SV dataset with the sveval R package, using the SVs called on the complete (not sampled) dataset for the corresponding cultivar as a ground truth

Breakpoint refinement was not performed on the SV datasets generated from these subsamples for computational efficiency reasons. For consistency, the complete (not sampled) truth SV sets against which subsampled datasets were compared were also not breakpoint-refined. However, we believe that the impact of breakpoint refinement on these comparisons would have been minimal and should not impact the conclusions drawn from these analyses.

Results of the subsampling analyses are presented in figure S7 (deletions), figure S8 (insertions), figure S9 (duplications) and figure S10 (inversions). In the context of this analysis, "sensitivity" represents the proportion of SVs in the complete dataset found in a

given subsample. Similarly, "precision" represents the proportion of SVs found in a given subsample that actually exist in the complete dataset.

The results obtained for deletions (figure S7) show that sensitivity exceeds 75% at a sequencing depth of 10X across all size classes and samples. The precision of deletion discovery is close to perfection across all size classes, and does not vary with sequencing depth. These results indicate that, for deletions, a sequencing depth of 10X is sufficient to detect the majority of variants and that the occurrence of false positive calls does not increase in samples that are sequenced at low depth.

The results obtained for insertions (figure S8) show that sensitivity exceeds 75% at a sequencing depth of 10X for insertions in the range 50 - 1,000 bp but decreases for larger insertions. Therefore, it appears that the effect of sequencing depth becomes more important as insertions increase in size. The precision of insertion discovery varies between 75% and close to 100% depending on insertion size, with the exception of insertions larger than 10 kb for a single sample (AC2001). These results suggest that insertion discovery is less accurate than deletion discovery. However, we observe the same pattern of independence between sequencing depth and precision as was observed for deletions, with the same exception as noted above. This again indicates that the accuracy of insertion calls from Oxford Nanopore data does not decrease in samples that are sequenced at lower depth.

The results obtained for duplications (figure S9) show that duplication discovery is more sensitive to sequencing depth than other SV types, with sensitivity below 75% at a sequencing depth of 10X. This result is not surprising given that duplication calling relies on observing higher coverage on some genomic interval compared to average, and such differences can be assessed more confidently in deeply sequenced samples. This result is also consistent with our observation that the apparent precision of duplication genotyping from Illumina data depends strongly on Oxford Nanopore sequencing depth (see figure S6). The precision of duplication discovery seen in figure S9 is also poorer than for other SV types and decreases slightly with decreasing sequencing depth, consistenly with difficulties in discovering and genotyping duplications as documented elsewhere in this paper.

Finally, the results obtained for inversions (figure S10) show sensitivity values similar to those observed for duplications, with sensitivity reaching between 50% and 75% at a sequencing depth of 10X. Inversion discovery appears to be highly sensitive to sequencing depth, as the relationship between sensitivity and sequencing depth does not seem to plateau. Precision rates are generally excellent ($> 90\%$) and do not depend on sequencing depth, which indicates that inversion discovery will generally be accurate in samples sequenced at lower depth. One exception to this result is smaller inversions (50 - 100 bp), as the small number of inversions observed in this size class (see table S1) caused stochastic variation in precision rates depending on the cultivar and subsample.

The observation that the precision rates of deletion, insertion and inversion discovery do not vary with sequencing depth is not surprising. Indeed, one would not expect a subsample of the reads to discover variants that were not already discovered from the full set of reads. However, Sniffles determines the coordinates of SVs by combining the information from all

reads supporting a given SV. Therefore, for some variants located in regions where reads are difficult to align properly, subsets of reads may describe variants differently from the complete set of reads. Similarly, variants that are supported by only a few misaligned reads might be called as homozygous from a sample of reads, but as heterozygous from the complete set and therefore filtered out. Both these situations would lead to false positive calls in the subsampled dataset relative to the full dataset, as assessed by the sveval package. Despite the possibility of those situations occurring, our results show that such false positive calls are rare for deletions, insertions, and inversions. Therefore, it is safe to call these SVs from low-depth Oxford Nanopore sequencing data without worrying about the impact of false positive calls.

Table S1: Number of filtered variants per SV type and size class discovered by Sniffles for 17 samples subjected to Oxford Nanopore sequencing. These SVs were used as a truth set for the benchmarks presented in this paper.

	[50bp – 100bp[				[100bp – 1kb[				[1kb – 10kb[				≥ 10kb			
	DEL[a]	INS[b]	DUP[c]	INV[d]	DEL	INS	DUP	INV	DEL	INS	DUP	INV	DEL	INS	DUP	INV
AC2001	2295	2591	23	3	2432	2643	541	56	1533	873	369	43	409	14	79	37
Alta	3389	3614	30	7	3441	4085	802	105	2321	1732	905	83	625	60	191	83
Maple Isle	3443	3546	19	7	3480	3910	597	90	2220	1492	496	69	533	46	147	70
Maple Presto	3037	3162	7	4	2904	2920	359	38	1616	362	197	29	361	0	34	26
OAC 09-35C	2861	2949	9	4	2887	3280	565	68	1970	1289	496	45	495	43	114	46
OAC Carman	2895	3006	13	4	2716	2830	425	30	1588	331	355	34	347	0	33	15
OAC Drayton	2473	2761	5	3	2551	2923	509	61	1686	1073	397	39	402	40	119	30
OAC Embro	2944	3176	6	4	2930	3150	408	47	1635	802	310	34	353	9	76	26
OAC Lakeview	2697	2993	16	4	2780	3313	650	69	1700	1122	602	54	384	32	125	46
OAC Madoc	2679	2945	10	2	2748	2935	449	61	1818	833	309	40	418	6	86	32
OAC Oxford	2678	2967	10	6	2772	3416	677	88	1809	1418	686	56	439	95	198	64
OAC Petrel	2670	2902	9	4	2746	3180	556	62	1893	1304	516	48	410	77	113	43
OAC Prudence	3095	3323	13	8	3249	3647	655	92	2140	1485	631	66	545	65	175	55
OAC Stratford	2676	2936	9	6	2794	3179	574	68	1865	1251	502	46	463	62	126	46
OT09-03	2983	3229	3	3	3057	3387	529	58	2013	1380	467	34	462	71	104	39
QS5091.50j	2433	2647	9	6	2486	2525	375	45	1472	424	216	32	369	0	67	31
Roland	3001	3118	12	6	3093	3429	551	55	2075	1426	533	51	477	72	131	42

[a] DEL: deletions
[b] INS: insertions
[c] DUP: duplications
[d] INV: inversions

Table S2: Number of deletions and insertions overlapping various genic features and proportion of these features within the reference annotation of Williams82

	Whole genome			Non-repeated genome		
Feature	% in reference	DEL[a] (%)	INS[b] (%)	% in reference	DEL (%)	INS (%)
cds[c]	6.4	1685 (5.5)	386 (1.5)	11.0	1617 (7.2)	372 (2.4)
gene	15.5	4329 (14.1)	3989 (15.2)	24.1	3806 (17.0)	3161 (20.3)
intergenic	60.0	15793 (51.5)	13893 (53.0)	39.3	9628 (43.0)	6509 (41.7)
upstream5kb[d]	18.1	8853 (28.9)	7967 (30.4)	25.6	7343 (32.8)	5562 (35.6)

[a] DEL: deletions
[b] INS: insertions
[c] cds: coding sequence
[d] upstream5kb: within 5 kb upstream of a gene

Table S3: Randomization test on the mean allele frequencies of SVs depending on the genic features overlapped

	Deletions		Insertions	
Genic features	Observed difference[a]	p-value[b]	Observed difference	p-value
cds[c]- gene	-0.0499	$< 10^{-4}$	0.0015	0.465
cds - intergenic	-0.0579	$< 10^{-4}$	-0.0199	0.085
cds - upstream5kb[d]	-0.0581	$< 10^{-4}$	0.0012	0.473
gene - intergenic	-0.0081	0.037	-0.0214	$< 10^{-4}$
gene - upstream5kb	-0.0082	0.049	-0.0003	0.477
intergenic - upstream5kb	-0.0001	0.484	0.0211	$< 10^{-4}$

[a] Observed difference between the mean allele frequencies of the SVs overlapping the corresponding genic features

[b] p-value computed from a one-sided comparison to the distribution of mean differences obtained from the randomizations (10,000 iterations). The significance threshold was set to $\alpha = 0.05 \div 6 = 0.008$ to correct for multiple testing.

[c] cds: coding sequence

[d] upstream5kb: within 5 kb upstream of a gene

Table S4: List of Gene Ontology Biological Process terms that are overrepresented ($\alpha = 0.05$) among coding sequences impacted by SVs with frequency > 0.5

GO ID	Description	Number of genes[a]	Expected[b]	Observed[c]	p-value[d]
GO:0006952	defense response	3218	31.86	73	2.771e-08
GO:1901420	negative regulation of response to alcohol	157	1.57	10	0.006
GO:0009788	negative regulation of abscisic acid-activated signaling pathway	157	1.57	10	0.006
GO:0032922	circadian regulation of gene expression	52	0.52	6	0.01771
GO:0010483	pollen tube reception	34	0.34	5	0.02852
GO:1905957	regulation of cellular response to alcohol	334	3.33	13	0.0494

[a] Number of genes with corresponding GO annotation within the gene universe (whole genome except unanchored scaffolds)
[b] Expected number of genes with given GO annotation within the subset of interest considering a hypergeometric distribution
[c] Observed number of genes with given GO annotation within the subset of interest
[d] Bonferroni-corrected p-values computed using the conditional hypergeometric test in the GOstats R package [54]

Table S5: List of Gene Ontology Biological Process terms that are underrepresented ($\alpha = 0.05$) among coding sequences impacted by SVs with frequency > 0.5

GO ID	Description	Number of genes[a]	Expected[b]	Observed[c]	p-value[d]
GO:1901576	organic substance biosynthetic process	10207	101.83	57	7.826e-05
GO:0034641	cellular nitrogen compound metabolic process	9963	99.39	56	0.0001638
GO:0009058	biosynthetic process	10423	103.98	60	0.000188
GO:0009059	macromolecule biosynthetic process	7143	71.26	34	0.0002087
GO:0044271	cellular nitrogen compound biosynthetic process	6818	68.02	32	0.0003073
GO:0044237	cellular metabolic process	20215	201.67	151	0.0004943
GO:0034645	cellular macromolecule biosynthetic process	6988	69.71	34	0.0005673
GO:0044249	cellular biosynthetic process	9988	99.64	58	0.000584
GO:0008152	metabolic process	23463	234.07	185	0.00105
GO:0034654	nucleobase-containing compound biosynthetic process	5166	51.54	22	0.002042
GO:1901360	organic cyclic compound metabolic process	9577	95.54	57	0.002998
GO:0018130	heterocycle biosynthetic process	5705	56.91	27	0.005327
GO:0006725	cellular aromatic compound metabolic process	9332	93.10	56	0.005933
GO:0019438	aromatic compound biosynthetic process	5953	59.39	29	0.00599
GO:0006139	nucleobase-containing compound metabolic process	8237	82.17	47	0.006126
GO:0046483	heterocycle metabolic process	8933	89.12	53	0.007338
GO:0016070	RNA metabolic process	6800	67.84	36	0.008725
GO:1901362	organic cyclic compound biosynthetic process	6214	61.99	32	0.01332
GO:0044238	primary metabolic process	19412	193.66	150	0.01586
GO:0032774	RNA biosynthetic process	4661	46.50	21	0.02269
GO:0097659	nucleic acid-templated transcription	4648	46.37	21	0.02466
GO:0071704	organic substance metabolic process	20977	209.27	167	0.03247
GO:0010556	regulation of macromolecule biosynthetic process	4874	48.62	23	0.03312
GO:0051171	regulation of nitrogen compound metabolic process	5638	56.25	29	0.03994
GO:2000112	regulation of cellular macromolecule biosynthetic process	4842	48.30	23	0.04037
GO:0090304	nucleic acid metabolic process	7519	75.01	44	0.04127
GO:0051252	regulation of RNA metabolic process	4681	46.70	22	0.04733
GO:0031323	regulation of cellular metabolic process	6248	62.33	34	0.04791

[a] Number of genes with corresponding GO annotation within the gene universe (whole genome except unanchored scaffolds)
[b] Expected number of genes with given GO annotation within the subset of interest considering a hypergeometric distribution
[c] Observed number of genes with given GO annotation within the subset of interest
[d] Bonferroni-corrected p-values computed using the conditional hypergeometric test in the GOstats R package [54]

Table S6: List of PFAM domains that are overrepresented ($\alpha = 0.05$) among coding sequences impacted by SVs with frequency > 0.5

PFAM ID	Description	Number of genes[a]	Expected[b]	Observed[c]	p-value[d]
PF00931	NB-ARC domain	353	3.46	42	3.011e-30
PF13676	TIR domain	156	1.53	20	1.844e-14
PF12776	Myb/SANT-like DNA-binding domain	90	0.88	14	4.998e-11
PF05699	hAT family C-terminal dimerisation region	98	0.96	10	8.656e-06
PF13947	Wall-associated receptor kinase galacturonan-binding	76	0.74	8	0.0001527
PF14380	Wall-associated receptor kinase C-terminal	36	0.35	6	0.0002372
PF13855	Leucine rich repeat	603	5.90	20	0.0004999
PF08263	Leucine rich repeat N-terminal domain	555	5.43	19	0.000564
PF14291	Domain of unknown function (DUF4371)	44	0.43	6	0.0008046
PF00560	Leucine Rich Repeat	443	4.34	16	0.001755
PF07714	Protein tyrosine and serine/threonine kinase	1089	10.66	25	0.01602
PF13962	Domain of unknown function	87	0.85	6	0.04042

[a] Number of genes with corresponding PFAM annotation within the gene universe (whole genome except unanchored scaffolds)
[b] Expected number of genes with given PFAM annotation within the subset of interest considering a hypergeometric distribution
[c] Observed number of genes with given PFAM annotation within the subset of interest
[d] Bonferroni-corrected p-values computed using the hypergeometric test in the GOstats R package [54]

Table S7: Metadata on the Illumina sequencing data of the 102 Canadian accessions

ID	Name	Read length[a]	Number of spots[b]	Average depth[c]	Core[d]	ONT[e]	SRA Run accession
CAD1001	OAC Bayfield	100	54,476,049	9.0	X		SRR5085868
CAD1002	OAC Prudence	100	36,410,197	6.1	X	X	SRR5085835
CAD1003	OAC Wallace	100	41,982,923	6.9	X		SRR5085790
CAD1004	AC Orford	100	50,401,243	8.6	X		SRR5085866
CAD1005	Gaillard	100	72,779,030	11.7	X		SRR5085843
CAD1006	OAC Prodigy	100	42,822,685	6.5			SRR5085847
CAD1007	AC Proteina	100	52,144,192	8.7	X		SRR5085740
CAD1008	Mandarin	100	49,771,834	8.4	X		SRR5085762
CAD1009	Evans	100	41,387,675	4.9	X		SRR5085794
CAD1010	Maple Presto	100	39,684,574	5.9	X	X	SRR5085755
CAD1011	OAC Ayton	100	40,549,216	6.5	X		SRR5085777
CAD1012	OAC Kent	100	49,397,571	7.3	X		SRR5085865
CAD1013	OAC Thames	100	36,279,267	6.5			SRR5085849
CAD1014	Dares	100	43,857,912	7.9	X		SRR5085854
CAD1015	OAC Lakeview	100	46,501,650	8.6	X	X	SRR5085805
CAD1016	OAC Morris	100	42,193,094	7.7	X		SRR5085773
CAD1017	Maple Glen	100	40,443,051	7.4	X		SRR5085792
CAD1018	Alta	100	44,399,003	8.2	X	X	SRR5085838
CAD1019	OAC Champion	100	40,955,178	7.4			SRR5085793
CAD1020	OT94-47	100	36,019,874	6.5	X		SRR5085763
CAD1021	OAC Erin	100	41,267,871	7.4			SRR5085769
CAD1022	OAC Petrel/OAC 07-03C	100	44,288,503	8.1	X	X	SRR5085761
CAD1023	Majesta	100	41,111,261	7.6			SRR5085756
CAD1025	12S01C-057	101	119,562,351	19.5			SRR5085784
CAD1026	12S04C-043	101	30,017,940	5.2			SRR5085764
CAD1027	QS06099.38J	101	38,526,261	5.7			SRR5085870
CAD1028	QS06099.38JB	101	40,253,878	6.8			SRR5085787
CAD1029	QS06009.25J	101	35,955,228	6.0			SRR5085749
CAD1030	QS06012.30J	101	81,031,579	12.7			SRR5085746

Continued on next page

Table S7: Metadata on the Illumina sequencing data of the 102 Canadian accessions *(continued)*

ID	Name	Read length[a]	Number of spots[b]	Average depth[c]	Core[d]	ONT[e]	SRA Run accession
CAD1031	QS06017.35j	101	26,858,820	4.5			SRR5085788
CAD1032	12S01C-074	101	16,831,411	2.6			SRR5085839
CAD1033	12S01C-075	101	41,895,598	7.2			SRR5085809
CAD1034	QS06008.6J	101	25,266,655	4.2			SRR5085863
CAD1035	12S01C-045	101	12,953,296	2.1			SRR5085766
CAD1036	12S02C-024	101	35,593,630	6.1			SRR5085770
CAD1037	QS06094.7J	101	42,294,490	7.0			SRR5085768
CAD1038	12S02C-026	101	11,452,044	1.9			SRR5085853
CAD1039	CERM13.5	101	73,000,044	11.7			SRR5085845
CAD1040	X5302-1-52-3-2-B	101	51,629,010	8.1			SRR5085850
CAD1041	X5346-1-3-4-4-B	101	41,403,699	6.3			SRR5085840
CAD1042	QS06087.4J	101	27,186,710	4.6			SRR5085871
CAD1043	QS07001-6	101	18,620,839	3.1			SRR5085765
CAD1044	X5377-1-43-4-2-B	101	47,728,960	7.8			SRR5085760
CAD1045	X5367-1-96-4-2-B	101	65,066,079	10.7			SRR5085848
CAD1046	QS4003.28B	101	13,828,872	2.3			SRR5085810
CAD1047	OAC Sparta	101	71,764,506	13.0			SRR5085791
CAD1048	OAC Inwood	101	81,965,973	13.8			SRR5085781
CAD1049	OAC Oxford	101	59,516,563	10.5	X	X	SRR5085758
CAD1050	QS4004P.4J	101	47,062,446	8.6	X		SRR5085796
CAD1051	Altesse	101	83,989,623	14.6	X		SRR5085867
CAD1052	OAC Embro	101	131,865,127	23.1		X	SRR5085774
CAD1053	OAC Ginty	101	101,126,961	18.1	X		SRR5085780
CAD1054	OAC Glencoe	101	35,447,080	6.0			SRR5085743
CAD1055	OAC Clinton	101	51,570,118	8.6	X		SRR5085795
CAD1056	OAC Drayton	101	64,429,867	11.7	X	X	SRR5085747
CAD1057	QS5055.43G	101	61,725,475	11.2	X		SRR5085776
CAD1058	OAC Atwood	101	31,532,679	5.3			SRR5085837
CAD1059	OAC Huron	101	15,567,042	2.7			SRR5085753
CAD1060	OAC 07-06C	101	40,678,337	7.3	X		SRR5085785

Continued on next page

Table S7: Metadata on the Illumina sequencing data of the 102 Canadian accessions *(continued)*

ID	Name	Read length[a]	Number of spots[b]	Average depth[c]	Core[d]	ONT[e]	SRA Run accession
CAD1061	QS4039P.15B(G)	101	71,277,321	12.7			SRR5085750
CAD1062	OAC Salem	101	51,294,544	8.1			SRR5085778
CAD1063	OAC Bright	101	28,185,844	4.8			SRR5085836
CAD1064	OAC Carman	101	51,517,811	8.8	X	X	SRR5085820
CAD1065	OAC Madoc	101	49,302,022	8.9	X	X	SRR5085775
CAD1066	QS4067P.17j	101	90,480,130	15.1	X		SRR5085754
CAD1067	QS5085.8Bp	101	39,175,548	6.6	X		SRR5085751
CAD1068	OAC Eclipse	101	62,222,843	10.7			SRR5085771
CAD1069	QS4042.6Bp	101	88,983,712	15.2	X		SRR5085741
CAD1070	QS5091.50j	101	15,715,364	2.6	X	X	SRR5085799
CAD1071	OAC Gretna	101	54,876,398	9.5	X		SRR5085822
CAD1072	DH618(OAC 06-18)	101	37,539,404	6.8	X		SRR5085786
CAD1073	Bloomfield	101	55,323,074	9.9	X		SRR5085834
CAD1074	OAC Stratford	101	91,317,467	16.4	X	X	SRR5085797
CAD1075	OT11-01	101	34,714,642	6.0	X		SRR5085782
CAD1076	SECAN09-42C	101	85,149,774	14.8	X		SRR5085798
CAD1077	AC 2001	101	79,719,863	13.9	X	X	SRR5085869
CAD1078	Kamichis	101	34,610,049	5.8	X		SRR5085824
CAD1079	AC Proteus	101	61,554,739	10.6	X		SRR5085744
CAD1080	Acme	101	40,866,110	7.5			SRR5085759
CAD1081	Maple Amber	101	78,290,219	14.0	X		SRR5085846
CAD1082	QGC16T	101	40,165,969	7.1			SRR5085812
CAD1083	AC Bravor	101	55,485,925	9.2	X		SRR5085752
CAD1084	Morsoy	101	73,648,479	13.0			SRR5085757
CAD1085	Rodeo	101	20,570,603	3.6			SRR5085841
CAD1086	OT05-18	101	65,940,587	11.3			SRR5085851
CAD1087	Roland	101	60,552,884	10.6	X	X	SRR5085821
CAD1088	SECAN08-24C	125	64,610,211	13.3	X		SRR5085800
CAD1089	OAC 09-35C	125	20,586,387	4.4	X	X	SRR5085789
CAD1090	9004	125	22,928,737	4.9	X		SRR5085823

Continued on next page

Table S7: Metadata on the Illumina sequencing data of the 102 Canadian accessions *(continued)*

ID	Name	Read length[a]	Number of spots[b]	Average depth[c]	Core[d]	ONT[e]	SRA Run accession
CAD1091	Heather	125	39,960,862	8.6	X		SRR5085842
CAD1092	Maple Isle	125	49,892,008	10.4	X	X	SRR5085745
CAD1093	9063	125	27,576,151	6.0	X		SRR5085864
CAD1094	OAC Eramosa	125	20,233,589	4.3			SRR5085783
CAD1095	Jari	125	16,971,129	3.7	X		SRR5085767
CAD1096	OT09-03	126	34,386,189	7.4	X	X	SRR5085844
CAD1097	Pagoda	126	52,954,119	11.3			SRR5085852
CAD1098	OT02-18	126	39,060,467	8.2			SRR5085772
CAD1099	X5165-1-28-3-5-B(Garry)	126	34,091,802	7.6			SRR5085742
CAD1100	Dundas	126	22,569,934	4.6	X		SRR5085861
CAD1101	Maple Donovan	126	27,507,002	6.2	X		SRR5085748
CAD1102	Toki	126	25,487,800	5.4	X		SRR5085811
CAD1103	Katrina	126	25,958,340	5.7	X		SRR5085779

[a] The length of a single sequence read
[b] The number of sequenced spots. Each spot corresponds to one forward and one reverse read.
[c] The average sequencing depth computed from reads mapped to assembled chromosome pseudomolecules (Gm01 .. Gm20)
[d] An "X" indicates that the sample belongs to the core set from which most Oxford Nanopore samples were selected
[e] An "X" indicates that the sample was sequenced using Oxford Nanopore Technologies sequencing

Table S8: DNA extraction and preparation methods for Oxford Nanopore sequencing

Sample[a]	Growth[b]	Grinding[c]	Extraction[d]	RNase T (°C)[e]	Size selection[f]	Combined[g]
AC2001 (1)	Lab	LN + TL	CTAB	37	BP 15kb	no
AC2001 (2)	Lab	LN + TL	CTAB	37	SRE	no
Alta	Lab	LN + TL	CTAB	37	BP 15kb	no
Maple Isle (1)	Lab	LN + TL	CTAB	37	BP 15kb	no
Maple Isle (2)	Lab	LN + TL	CTAB	60	SRE	no
Maple Presto	Field	CD + TL	DNeasy	65	BP 6kb	yes
OAC 09-35C	Lab	LN + TL	CTAB	37	BP 15kb	no
OAC Carman	Field	CD + TL	DNeasy	65	BP 6kb	yes
OAC Drayton	Lab	LN + TL	CTAB	37	BP 15kb	no
OAC Embro	Greenhouse	LN + MP	Gentra	37	None	no
OAC Lakeview (1)	Lab	LN + TL	CTAB	37	BP 15kb	no
OAC Lakeview (2)	Lab	LN + TL	CTAB	60	SRE	no
OAC Madoc	Lab	LN + TL	CTAB	37	BP 15kb	no
OAC Oxford	Lab	LN + TL	CTAB	37	BP 15kb	no
OAC Petrel	Lab	LN + TL	CTAB	60	SRE	no
OAC Prudence	Lab	LN + TL	CTAB	37	BP 15kb	no
OAC Stratford	Lab	LN + TL	CTAB	37	BP 15kb	no
OT09-03	Lab	LN + TL	CTAB	65	SRE	no
QS5091.50j	Field	CD + TL	DNeasy	65	BP 6kb	no
Roland	Lab	LN + TL	CTAB	60	SRE	no

[a] The number in parentheses identifies samples sequenced on two different flowcells

[b] Field: leaf harvested at Agriculture and Agri-Food Canada in Ottawa, and cryodessicated on site

[c] Grinding method: LN + TL, Frozen in liquid nitrogen and ground using Qiagen TissueLyser;
CD + TL, Cryodessicated at harvest and ground using Qiagen TissueLyser;
LN + MP, Frozen in liquid nitrogen and ground using mortar and pestle

[d] Extraction protocol used: CTAB, CTAB-based protocol described in main text;
DNeasy, Qiagen DNeasy Plant Mini Kit;
Gentra, Qiagen Gentra Puregene Tissue Kit

[e] Incubation temperature of the RNase A treatment

[f] Size-selection protocol: None, no size-selection;
BP 6kb, BluePippin with 6-kb size-selection threshold;
BP 15kb, BluePippin High-Pass Plus cassette with 15-kb size-selection threshold;
SRE, Circulomics Short Read Eliminator Kit

[g] Indicates whether the DNA from two different extractions was combined for sequencing

Table S9: Metadata on Oxford Nanopore sequencing runs

Sample[a]	Date[b]	Active pores[c]	Mass (ng)[d]	Run time (h)[e]	Yield (Gb)[f]	Read N50 (kb)[g]
AC2001 (1)	13-03-2020	1652	210	72	9.3	6.0
AC2001 (2)	27-07-2020	1586	309	72	13.9	19.7
Alta	13-03-2020	1640	196	72	30.4	11.2
Maple Isle (1)	11-06-2020	1477	166	72	6.6	4.1
Maple Isle (2)	10-08-2020	1518	354	72	15.7	14.8
Maple Presto	01-10-2019	1568	364	48	10.2	5.2
OAC 09-35C	21-05-2020	1613	109	72	12.8	17.5
OAC Carman	09-10-2019	1678	146	48	15.6	3.8
OAC Drayton	15-06-2020	1461	118	72	11.1	18.3
OAC Embro	14-03-2019	NA	244	48	16.3	6.9
OAC Lakeview (1)	15-06-2020	1478	112	72	4.8	21.0
OAC Lakeview (2)	10-08-2020	1347	339	72	15.3	8.1
OAC Madoc	21-05-2020	1589	187	72	10.2	12.0
OAC Oxford	26-05-2020	1471	218	72	16.7	22.0
OAC Petrel	31-07-2020	1559	407	72	9.6	29.5
OAC Prudence	11-06-2020	1402	262	72	13.5	20.7
OAC Stratford	26-05-2020	1177	225	72	12.5	18.9
OT09-03	25-02-2020	1392	248	72	10.1	24.8
QS5091.50j	11-09-2019	1190	225	48	11.4	6.7
Roland	07-07-2020	1774	168	72	10.9	25.6

[a] The number in parentheses identifies samples sequenced on two different flowcells
[b] Date when the sequencing run was started, format dd-mm-yyyy
[c] Number of active pores on the flowcell prior to sequencing
[d] Total estimated mass of the DNA library that was loaded on the flowcell
[e] Duration of the sequencing run
[f] Total number of bases in the fastq files following basecalling
[g] Read N50 of all reads following basecalling

Table S10: Classification of deletions and insertions for four samples used to test the SV refinement pipeline

Sample	Refined[a]	Below thresholds[b]	Above max. SVLEN[c]	No alignment[d]
Maple Presto	9137	1597	19	2881
OAC Carman	6937	1434	6	4192
OAC Embro	11469	2684	24	584
QS5091.50j	9344	1804	17	734

[a] SVs that passed all filters and had their breakpoints and/or sequence content updated by the pipeline

[b] Did not pass one or several thresholds applied when comparing the proposed refined SV to the raw SV

[c] SV length above the maximum permissible length (50 kb used here)

[d] No assembly or no alignment produced by the pipeline

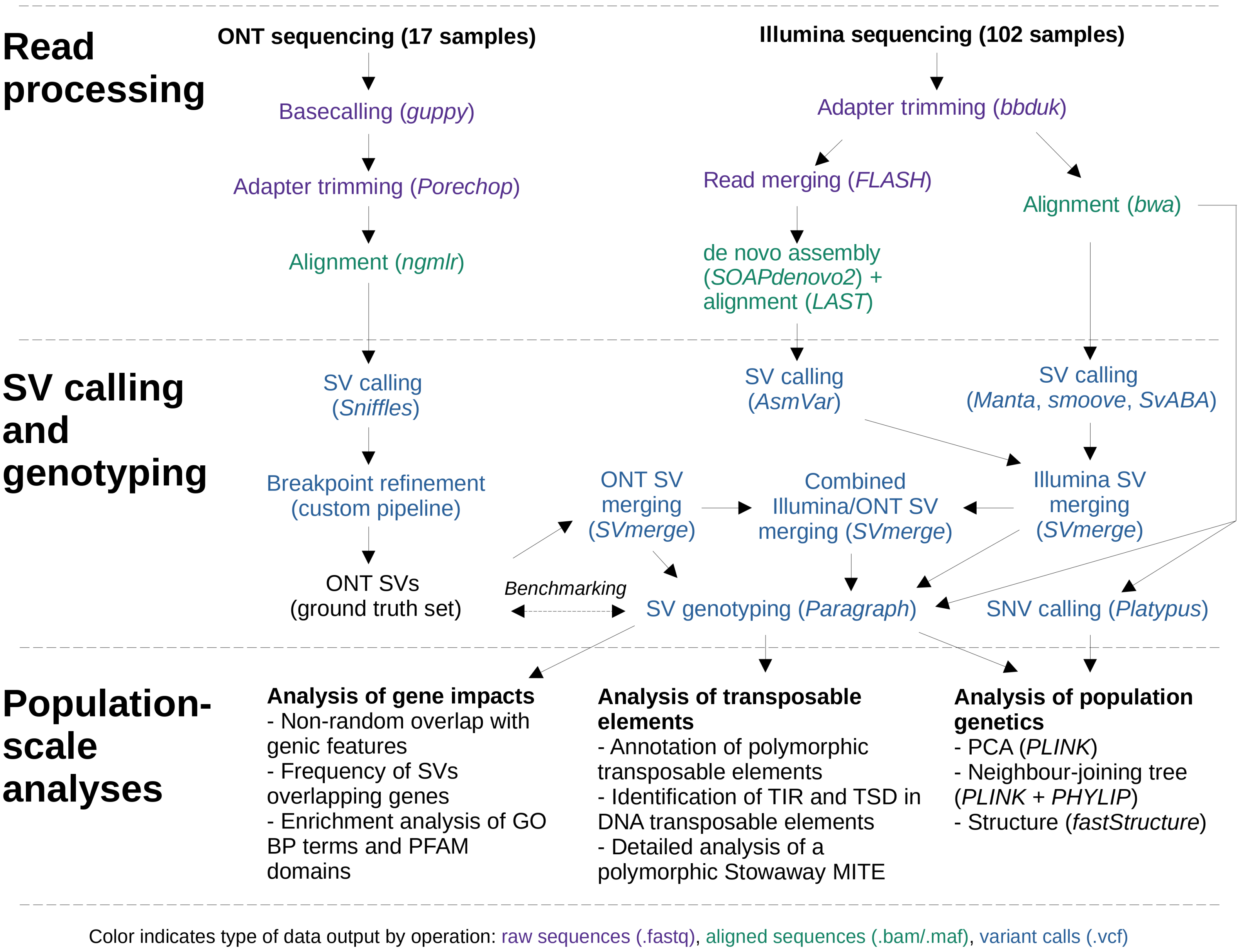

Caption on next page

Figure S1: Graphical summary of the methods used in this paper. The various steps of the analysis are color-coded depending on the data output by that step (see legend at the bottom of the figure). The words in italics in parentheses indicate the name of program used to compute that part of the analysis, where applicable. Arrows between steps indicate that one step is a prerequisite to the step it is pointing to. An exception to this is the dotted arrow indicating that both predicted SV genotypes and the ground truth Oxford Nanopore SV dataset are necessary to benchmarking. Benchmarks were performed for three different candidate SV datasets genotyped using Illumina data: SVs discoverered from Illumina data only, SVs discovered from Oxford Nanopore data only, and the combination of SVs discovered from the Illumina and Oxford Nanopore data. Population-scale analyses were performed on the SV dataset based on combined Illumina and Oxford Nanopore SVs. ONT: Oxford Nanopore Technologies, SV: structural variant, SNV: single-nucleotide variant, GO: Gene Ontology, BP: Biological Process, TIR: terminal inverted repeat, TSD: target site duplication, MITE: miniature inverted-repeat transposable element, PCA: principal component analysis.

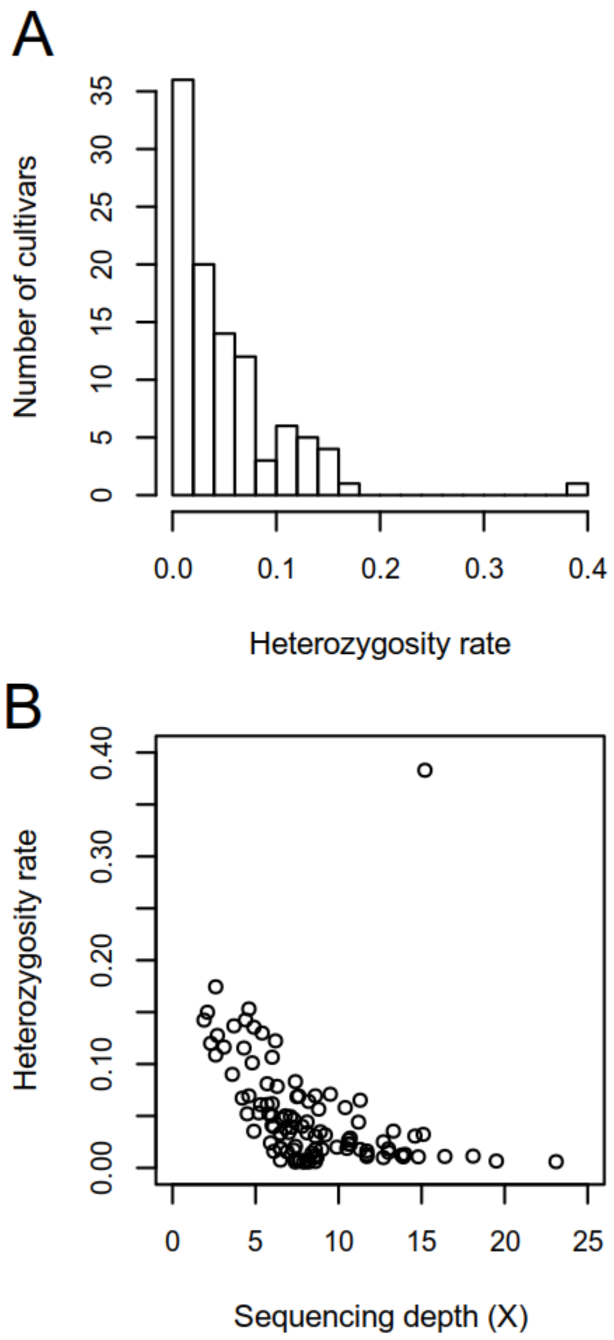

Figure S2: Heterozygosity rates of the population of 102 Canadian soybean cultivars as assessed from a set of filtered SNVs called by Platypus. (A) Distribution of heterozygosity rates of the 102 cultivars displayed as a histogram. (B) Relationship between the heterozygosity rate of a cultivar and its Illumina sequencing depth. The sequencing depth values shown are the same as in table S7 and represent the effective sequencing depth computed from mapped reads. With the exception of one outlier, heterozygosity rates higher than 0.1 are attributable to shallow sequencing, which can lead to genotyping errors resulting in seemingly higher heterozygosity. Heterozygous calls in the context of inbred lines may occur due to various causes including genotype-calling errors in low-depth samples (as shown here), copy number variants generating spurious heterozygous genotype calls [6], or genuine residual heterozygosity.

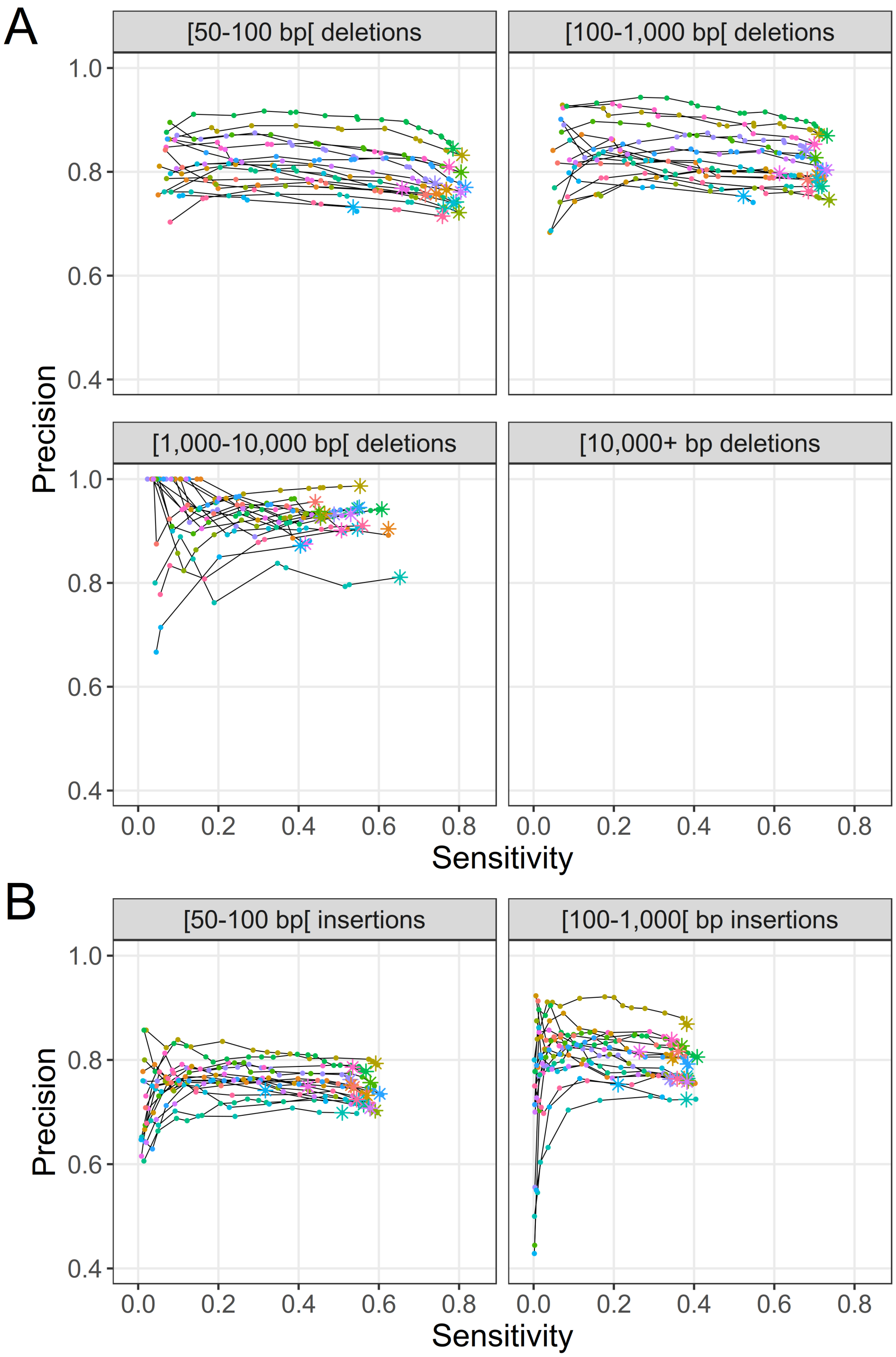

Figure S3: Genotyping sensitivity and precision of (A) deletions and (B) insertions discovered from the Illumina data in non-repeat regions. Only SVs with less than 20% overlap to regions annotated as repeats were used for these benchmarks. Each line and color represents one of 17 samples. The points correspond to different filtering thresholds on the minimum number of Illumina reads required to support a genotype call. The asterisks indicate a minimum number of supporting reads of 2; points to the left of these for a given line represent increasingly stringent filtering threshold values. Note that no results were available for deletions larger than 10 kb because these all overlapped repeat regions.

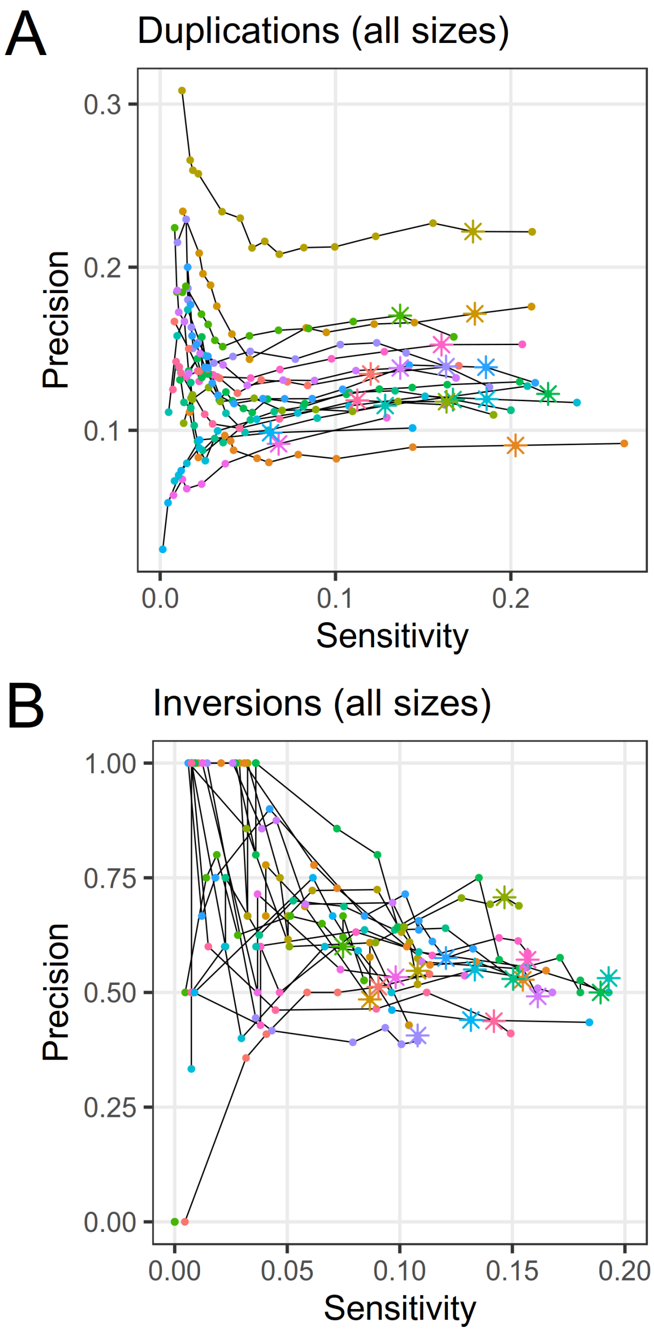

Figure S4: Genotyping sensitivity and precision of (A) duplications and (B) inversions discovered from the Illumina data. Each line and color represents one of 17 samples. The points correspond to different filtering thresholds on the minimum number of Illumina reads required to support a genotype call. The asterisks indicate a minimum number of supporting reads of 2; points to the left of these for a given line represent increasingly stringent filtering threshold values.

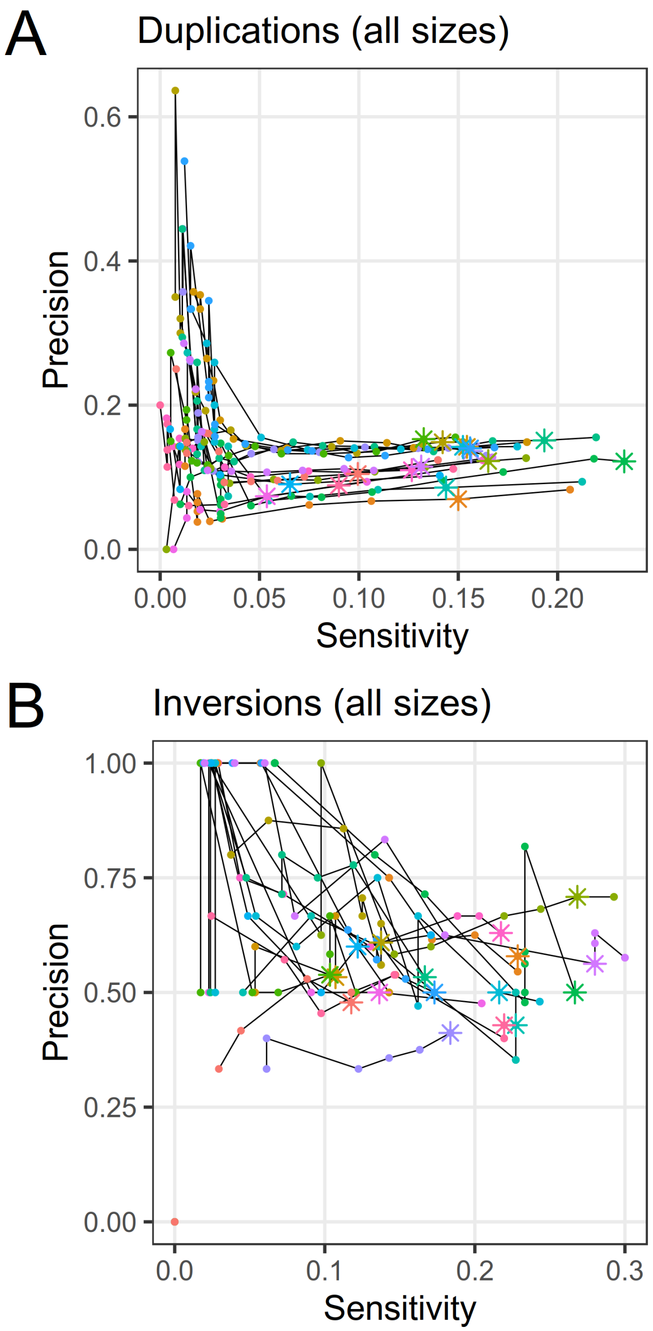

Figure S5: Genotyping sensitivity and precision of (A) duplications and (B) inversions discovered from the Illumina data in non-repeat regions. Only SVs with less than 20% overlap to regions annotated as repeats were used for these benchmarks. Each line and color represents one of 17 samples. The points correspond to different filtering thresholds on the minimum number of Illumina reads required to support a genotype call. The asterisks indicate a minimum number of supporting reads of 2; points to the left of these for a given line represent increasingly stringent filtering threshold values.

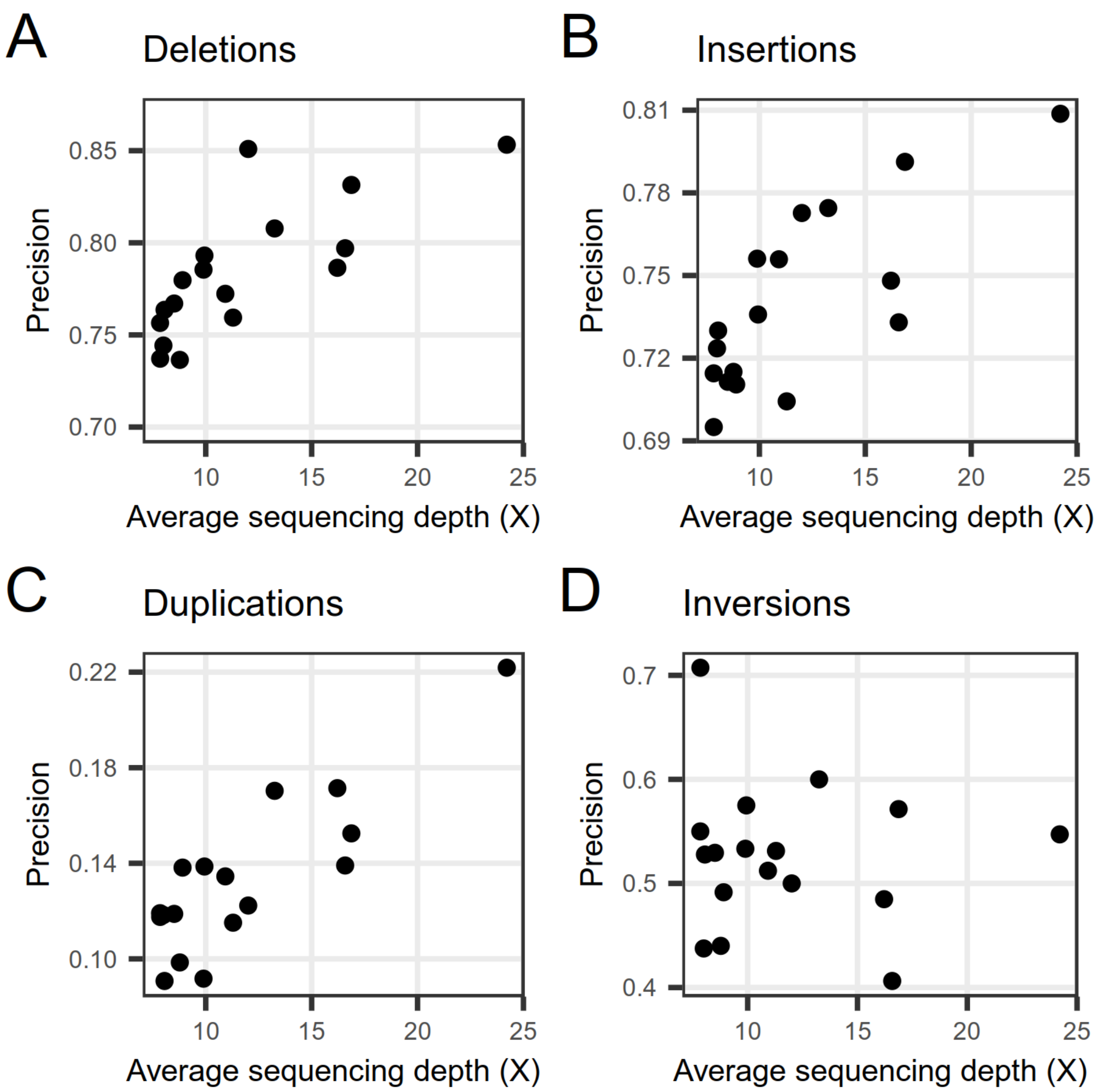

Figure S6: Genotyping precision of (A) deletions, (B) insertions, (C) duplications and (D) inversions discovered from Illumina data (at a minimum threshold of 2 supporting reads) for 17 samples as a function of the average Oxford Nanopore sequencing depth of that sample.

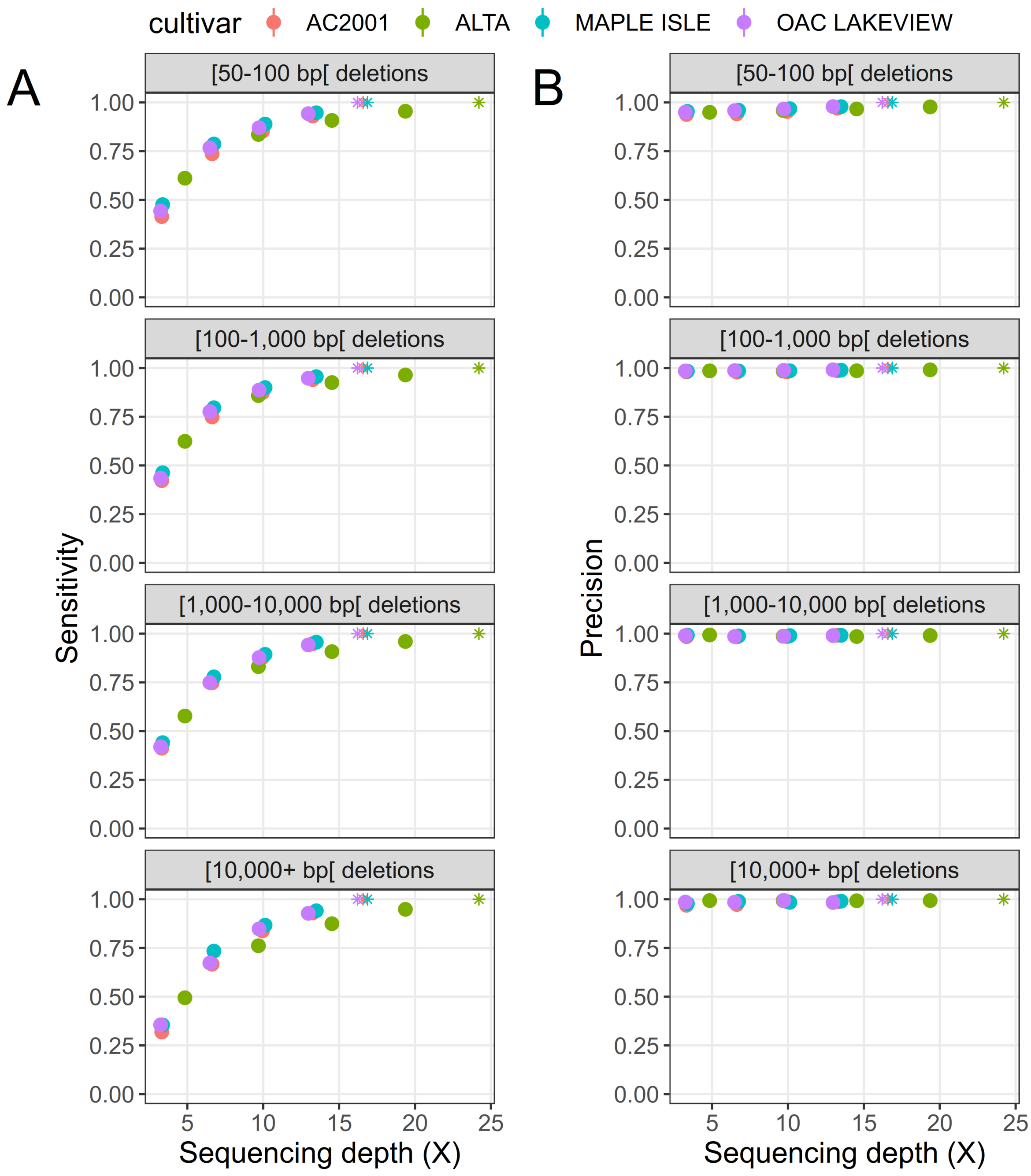

Figure S7: Effects of sequencing depth on the sensitivity and precision of deletion discovery from Oxford Nanopore sequencing data. Oxford Nanopore reads of four cultivars were subsampled using subsampling fractions of 20%, 40%, 60% and 80%, with five replicates of each cultivar/subsampling fraction combination. SVs were called on each of these subsamples and (A) sensitivity and (B) precision computed by comparing the resulting SVs to the SVs called on the full (not sampled) dataset for the corresponding cultivar. Points indicate the median value for a given cultivar/subsampling fraction combination, while vertical error bars indicate the span from the minimum to the maximum value of five replicates. Asterisks indicate the value (1 by definition) obtained for the full dataset. Error bars are not visible in cases where the minimum and maximum values are too close to each other.

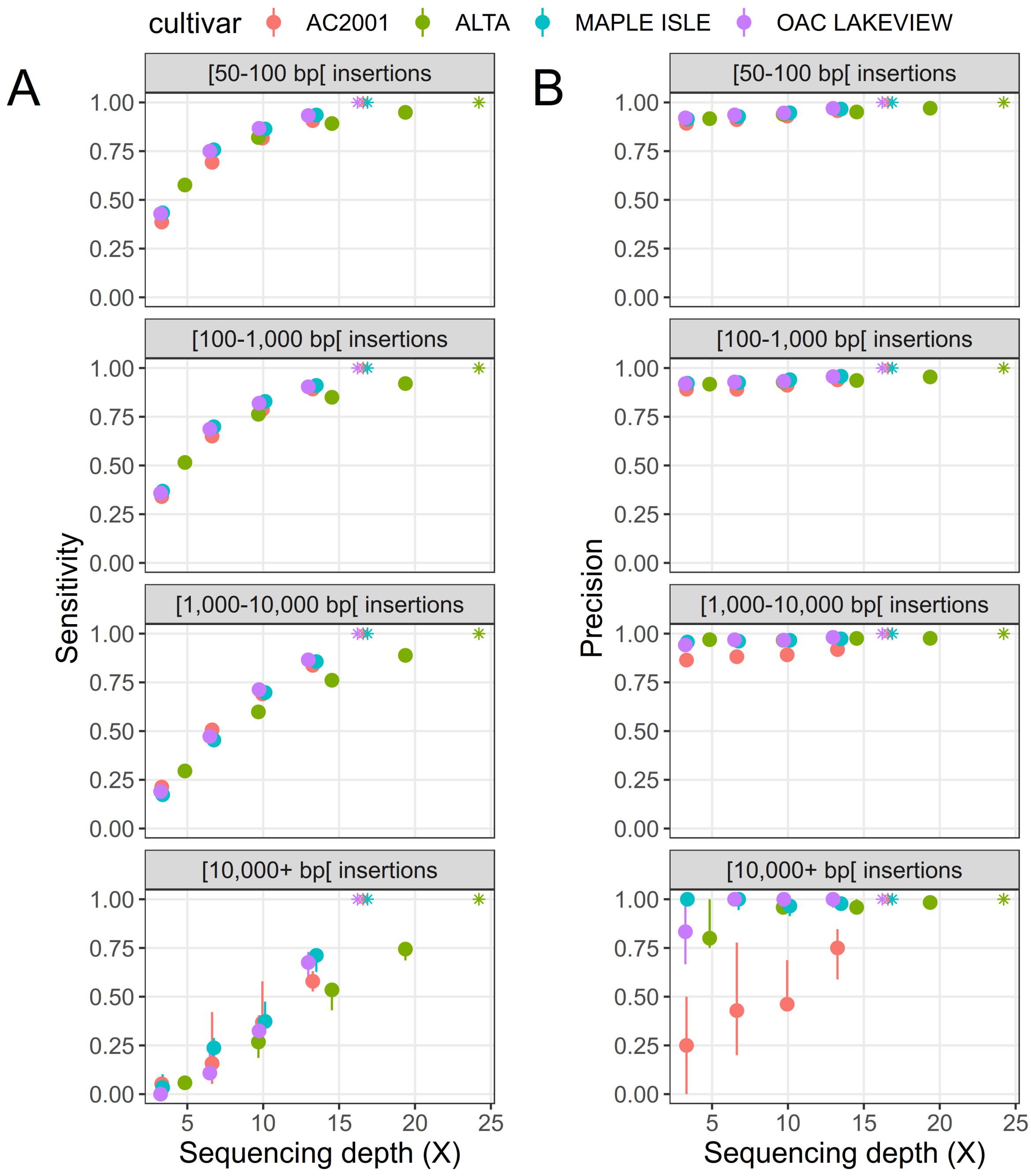

Figure S8: Effects of sequencing depth on the sensitivity and precision of insertion discovery from Oxford Nanopore sequencing data. Oxford Nanopore reads of four cultivars were subsampled using subsampling fractions of 20%, 40%, 60% and 80%, with five replicates of each cultivar/subsampling fraction combination. SVs were called on each of these subsamples and (A) sensitivity and (B) precision computed by comparing the resulting SVs to the SVs called on the full (not sampled) dataset for the corresponding cultivar. Points indicate the median value for a given cultivar/subsampling fraction combination, while vertical error bars indicate the span from the minimum to the maximum value of five replicates. Asterisks indicate the value (1 by definition) obtained for the full dataset. Error bars are not visible in cases where the minimum and maximum values are too close to each other.

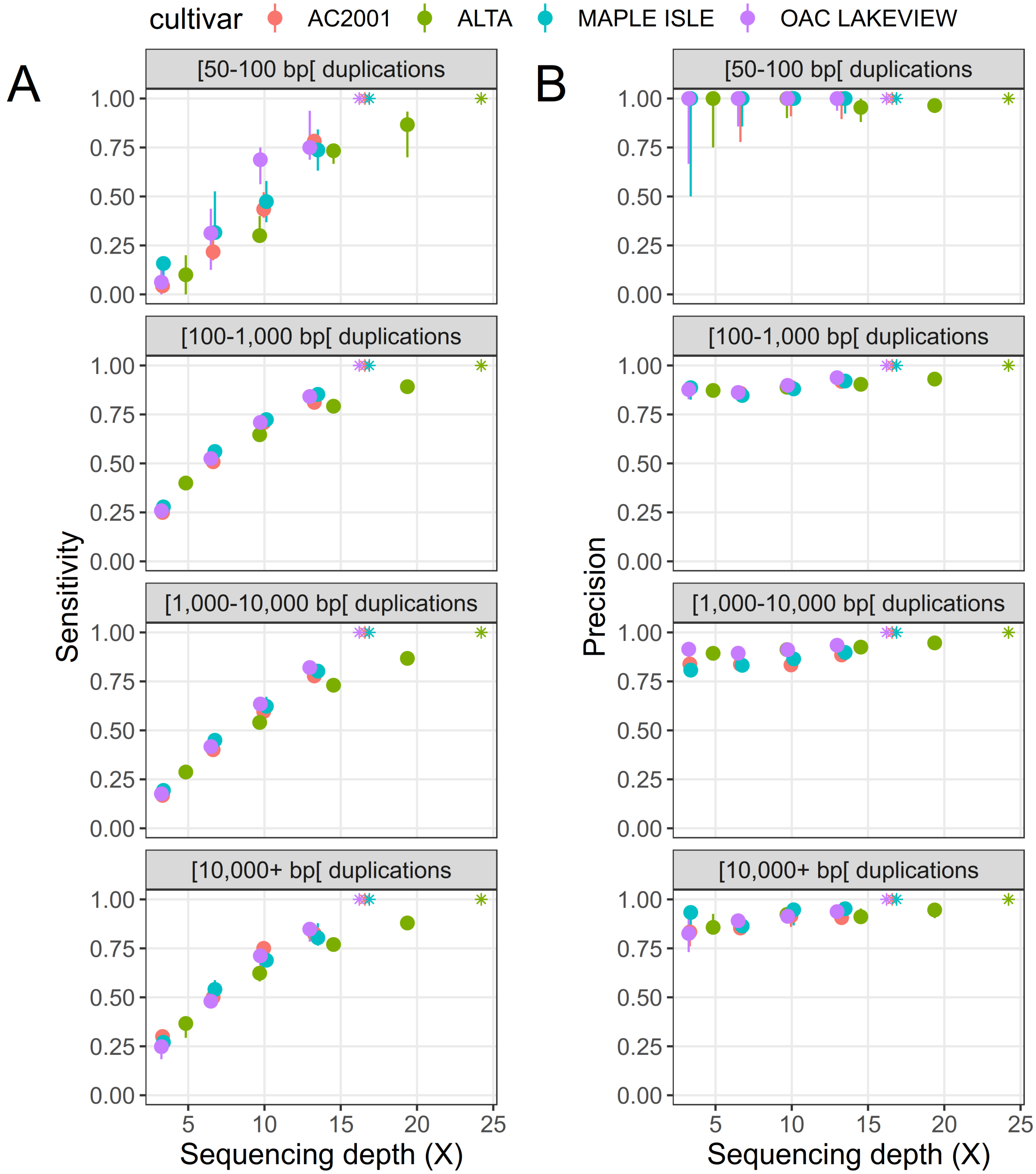

Figure S9: Effects of sequencing depth on the sensitivity and precision of duplication discovery from Oxford Nanopore sequencing data. Oxford Nanopore reads of four cultivars were subsampled using subsampling fractions of 20%, 40%, 60% and 80%, with five replicates of each cultivar/subsampling fraction combination. SVs were called on each of these subsamples and (A) sensitivity and (B) precision computed by comparing the resulting SVs to the SVs called on the full (not sampled) dataset for the corresponding cultivar. Points indicate the median value for a given cultivar/subsampling fraction combination, while vertical error bars indicate the span from the minimum to the maximum value of five replicates. Asterisks indicate the value (1 by definition) obtained for the full dataset. Error bars are not visible in cases where the minimum and maximum values are too close to each other.

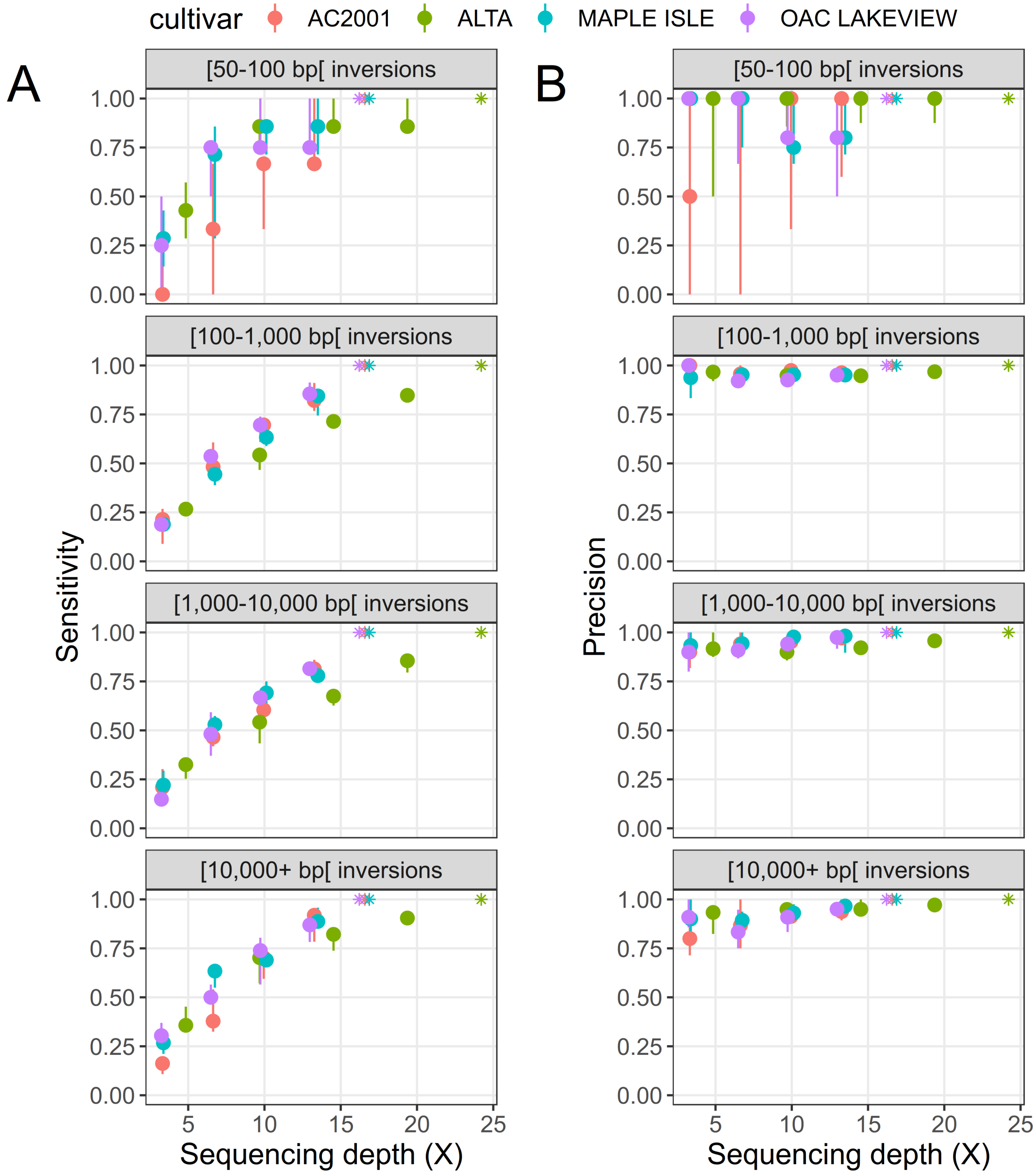

Figure S10: Effects of sequencing depth on the sensitivity and precision of inversion discovery from Oxford Nanopore sequencing data. Oxford Nanopore reads of four cultivars were subsampled using subsampling fractions of 20%, 40%, 60% and 80%, with five replicates of each cultivar/subsampling fraction combination. SVs were called on each of these subsamples and (A) sensitivity and (B) precision computed by comparing the resulting SVs to the SVs called on the full (not sampled) dataset for the corresponding cultivar. Points indicate the median value for a given cultivar/subsampling fraction combination, while vertical error bars indicate the span from the minimum to the maximum value of five replicates. Asterisks indicate the value (1 by definition) obtained for the full dataset. Error bars are not visible in cases where the minimum and maximum values are too close to each other.

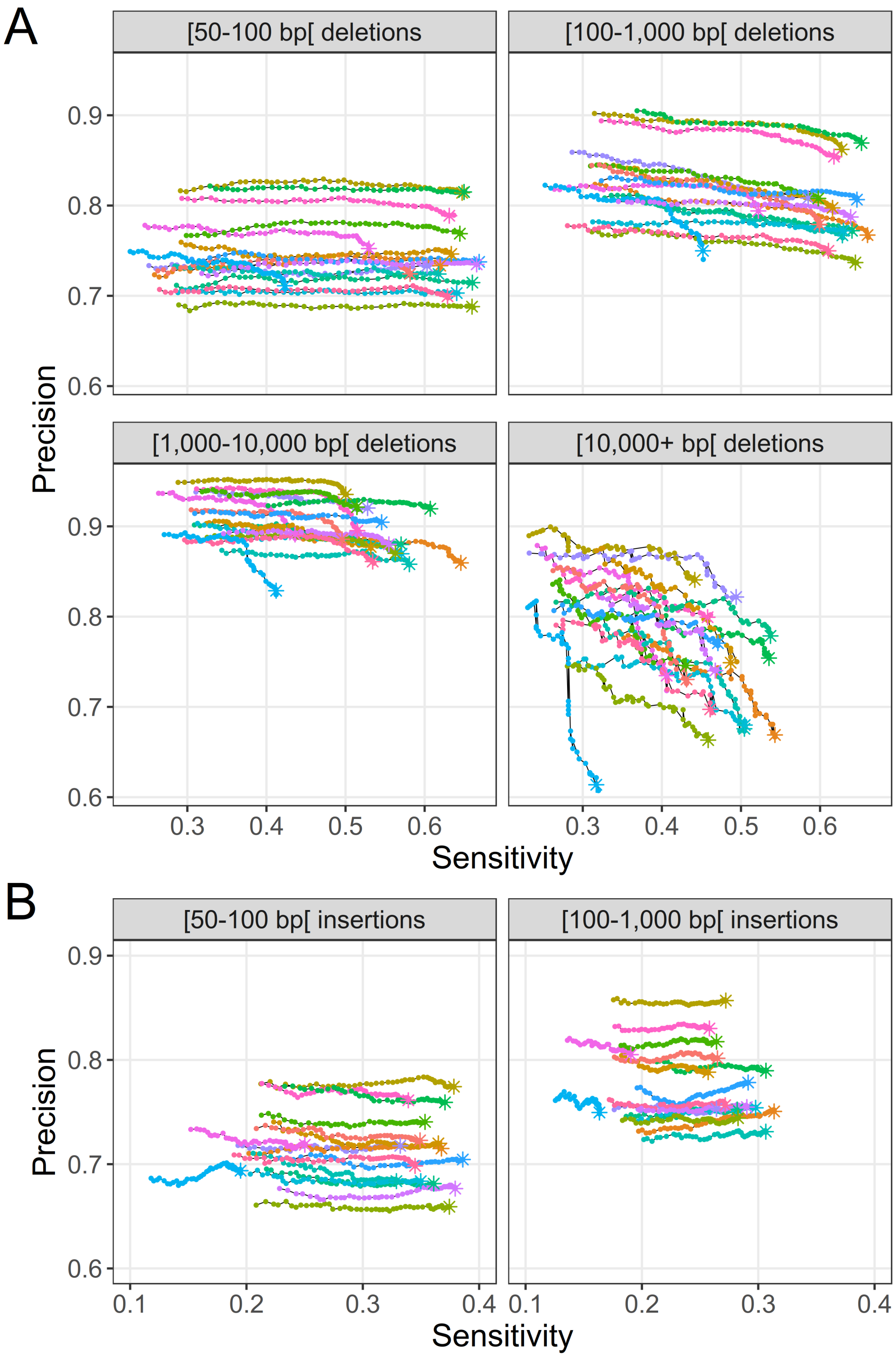

Figure S11: Effect of filtering variants for minimum number of ALT alleles in homozygous genotype calls (homozygous ALT count) on the genotyping performance of (A) deletions and (B) insertions discovered from Illumina data. Each line and color represents a different sample. The curve for a given sample was obtained by varying the filtering threshold for a variant to be considered for benchmarking. The asterisks mark a homozygous ALT count threshold of 4; points to the left of these for a given line represent increasingly stringent filtering threshold values.

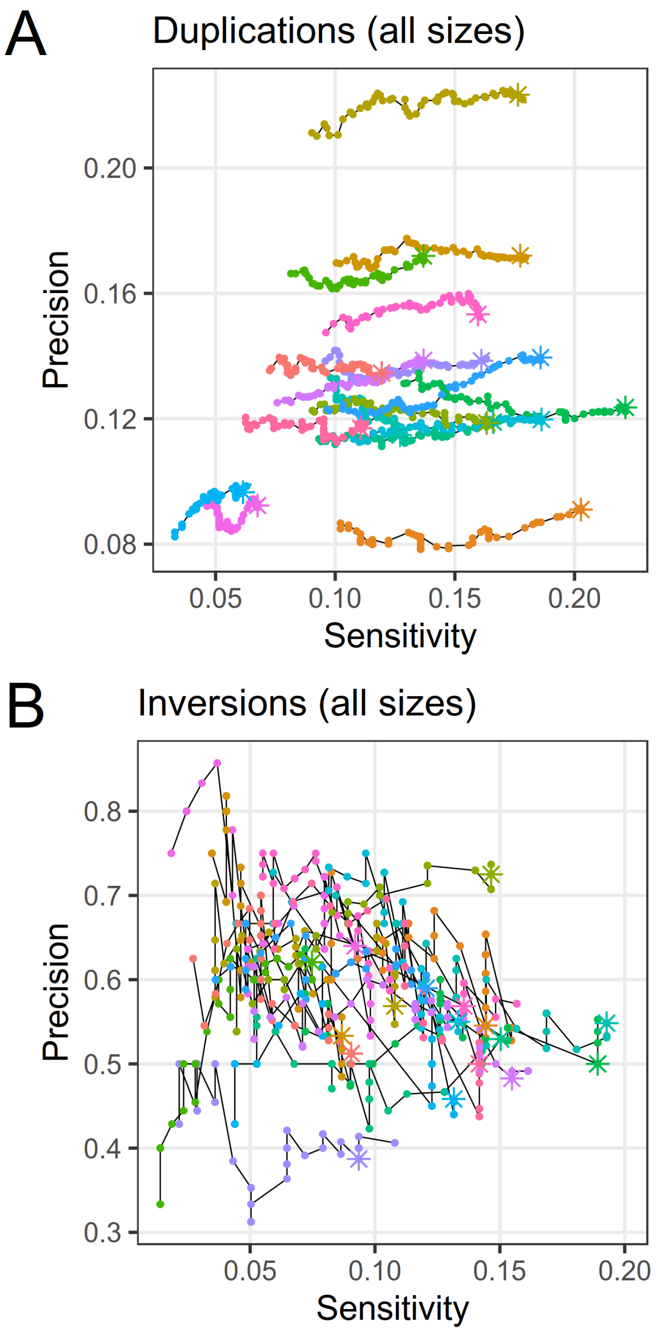

Figure S12: Effect of filtering variants for minimum number of ALT alleles in homozygous genotype calls (homozygous ALT count) on the genotyping performance of (A) duplications and (B) inversions discovered from Illumina data. Each line and color represents a different sample. The curve for a given sample was obtained by varying the filtering threshold for a variant to be considered for benchmarking. The asterisks mark a homozygous ALT count threshold of 6 (for duplications) or 10 (for inversions); points to the left of these for a given line represent increasingly stringent filtering threshold values.

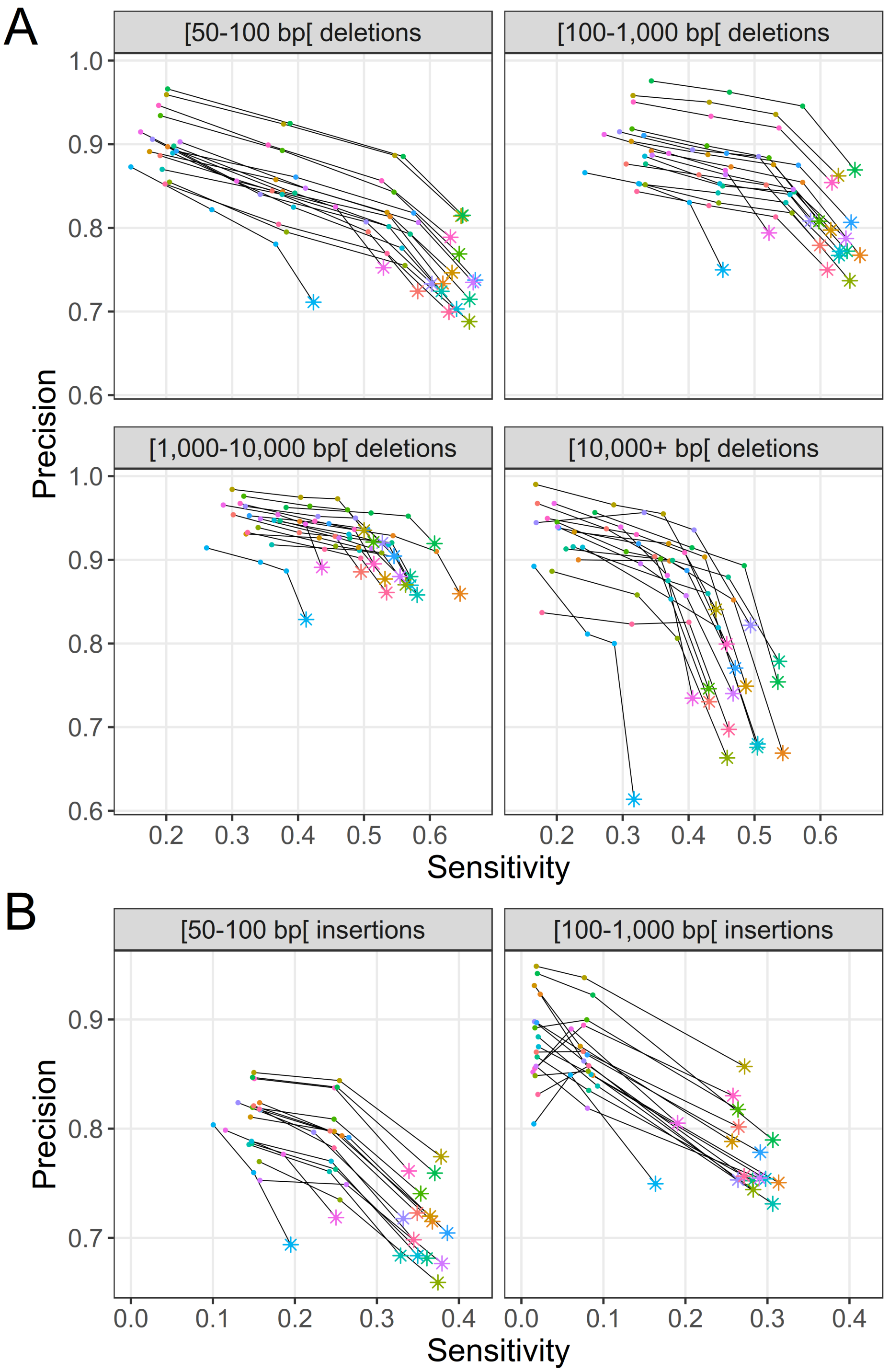

Figure S13: Effect of filtering variants for minimum number of supporting calling tools on the genotyping performance of (A) deletions and (B) insertions discovered from Illumina data. Each line and color represents a different sample. The curve for a given sample was obtained by varying the minimum threshold of number of supporting calling tools for a variant to be considered for benchmarking. The asterisks mark a minimum threshold of one supporting calling tool for a variant to be considered; points to the left of these for a given line represent increasingly stringent filtering threshold values.

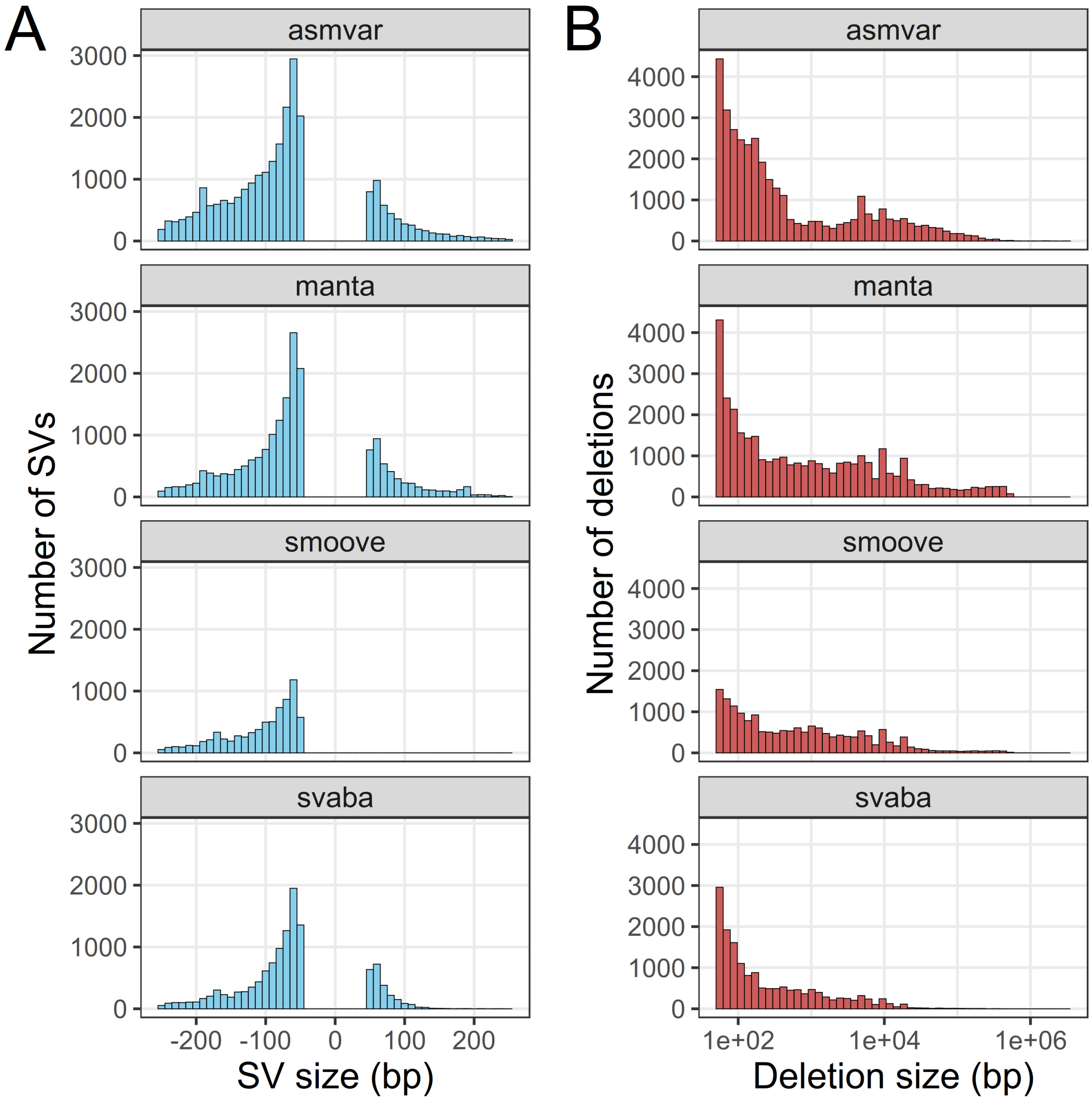

Figure S14: Size distribution of the SVs reported by various calling tools using Illumina data. (A) Distribution of SV sizes in the range from -250 bp to 250 bp. Negative sizes represent deletions and positive sizes represent insertions. Duplications and inversions were omitted from this plot. (B) Distribution of the absolute size of deletions on a logarithmic scale.

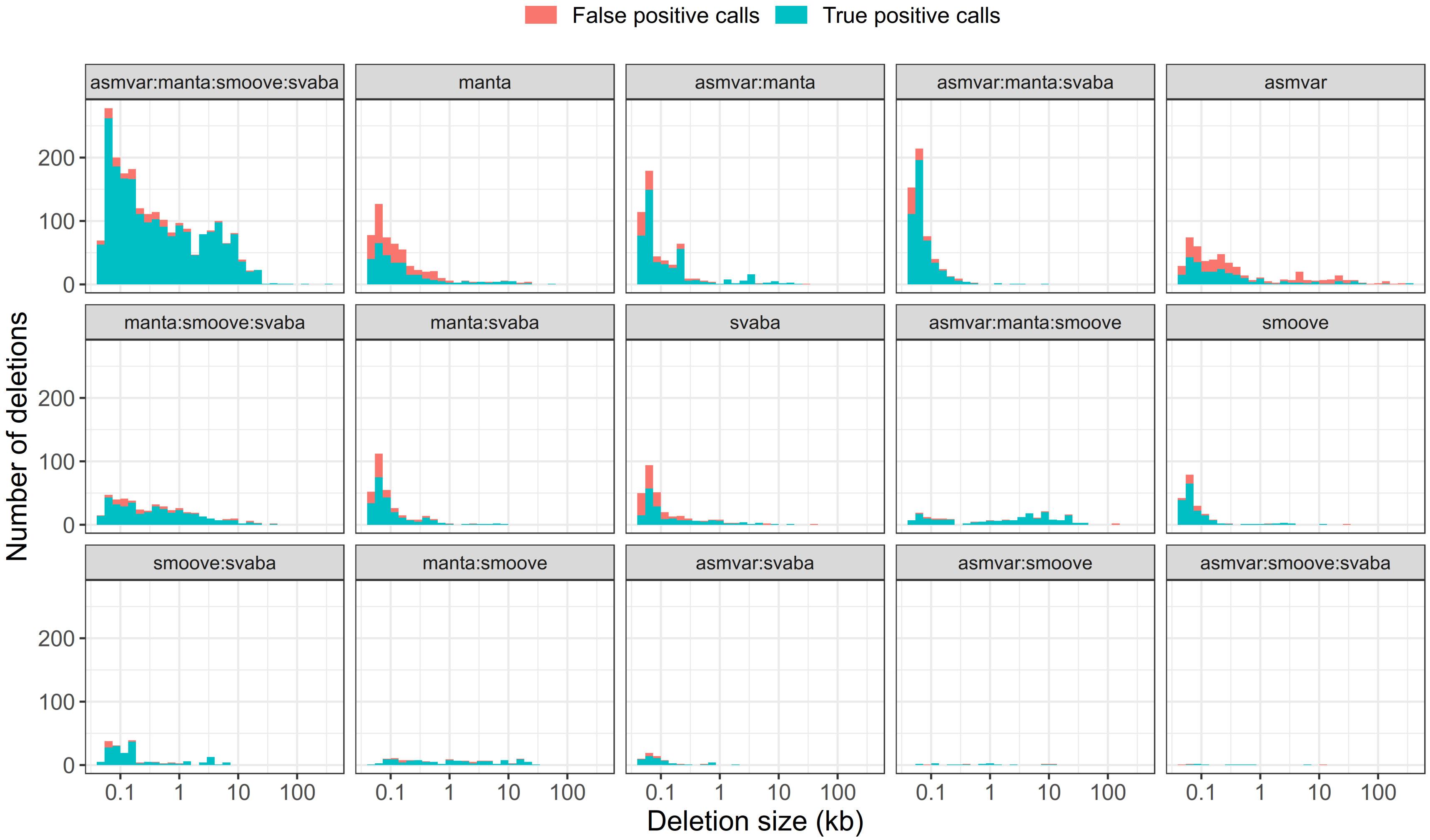

Figure S15: Number of true positive and false positive deletion genotype calls broken down by size and by the combination of tools that reported the variant for sample OAC Oxford. OAC Oxford was chosen as a representative sample because of its median F1 score among all 17 benchmarked samples. The genotype calls considered here were filtered for a minimum number of supporting reads of 2 and a minimum homozygous ALT count of 4 across all 102 samples in the population. Note the logarithmic scale on the x-axis.

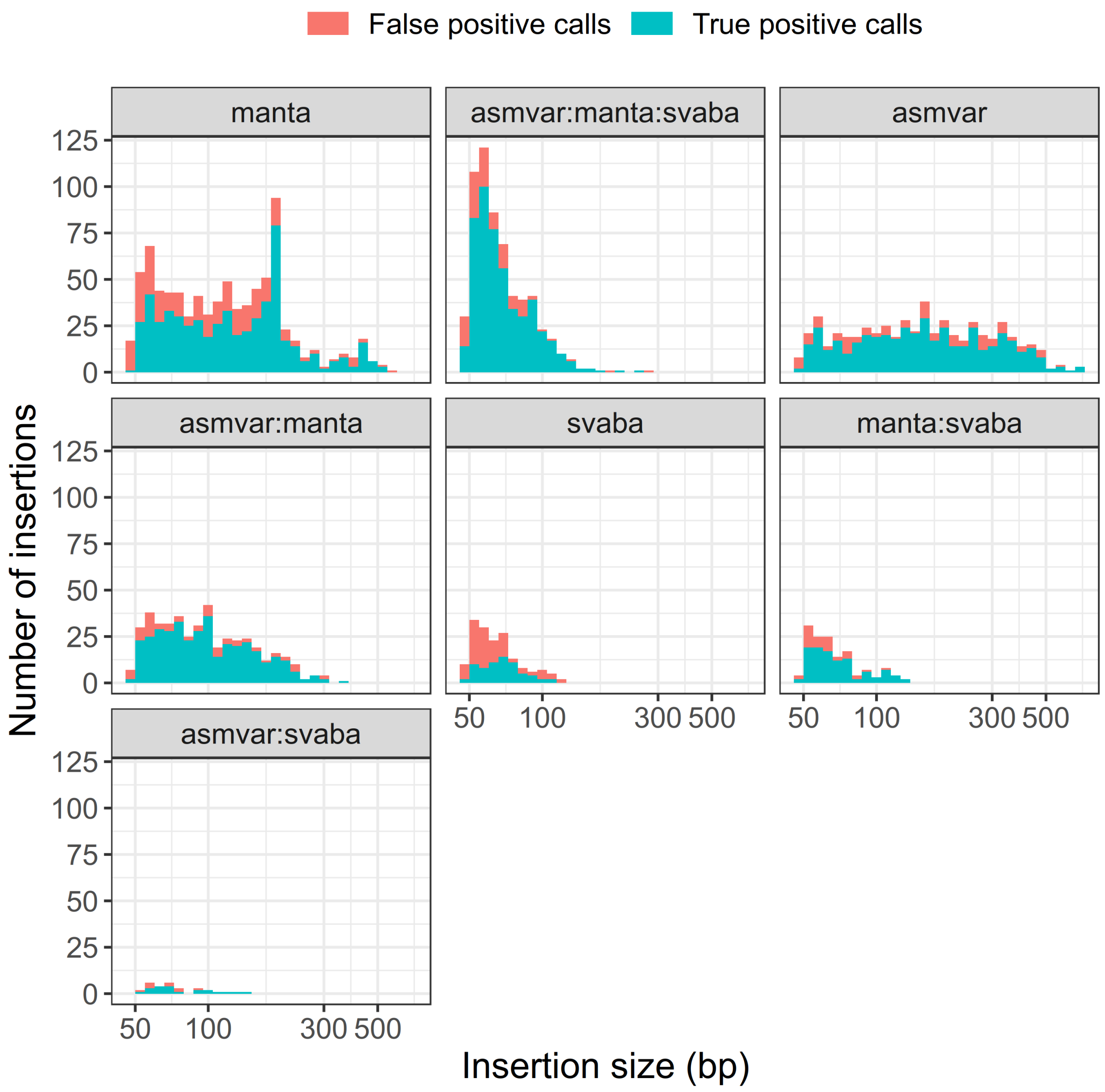

Figure S16: Number of true positive and false positive insertion genotype calls broken down by size and by the combination of tools that reported the variant for sample OAC Lakeview. OAC Lakeview was chosen as a representative sample because of its median F1 score among all 17 benchmarked samples. The genotype calls considered here were filtered for a minimum number of supporting reads of 2 and a minimum homozygous ALT count of 4 across all 102 samples in the population. Note the logarithmic scale on the x-axis.

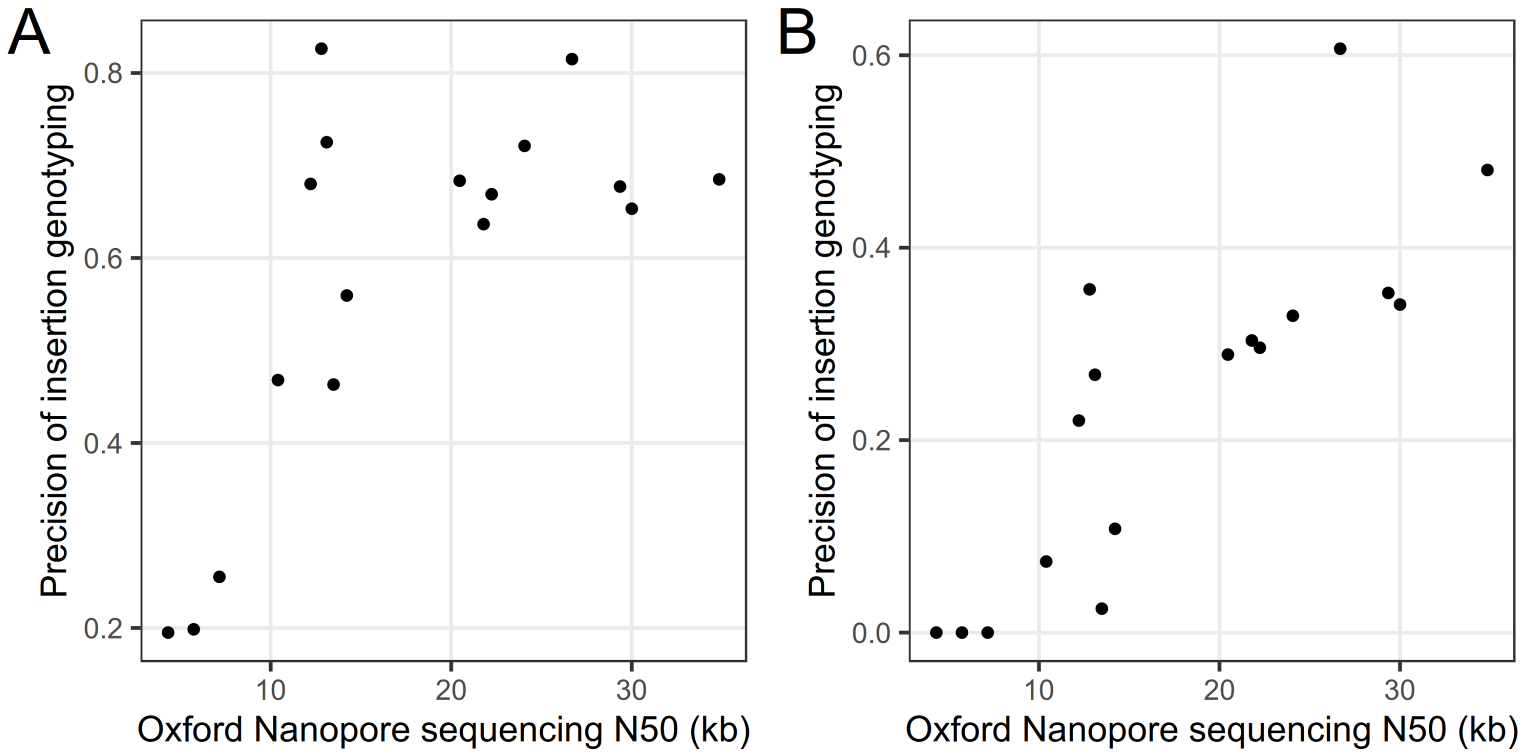

Figure S17: Genotyping precision of insertions discovered from Oxford Nanopore data (at a minimum threshold of 2 supporting reads) of 17 samples as a function of the read N50 of the Oxford Nanopore run(s) of that sample. (A) Precision of genotyping for insertions in the range 1,000 – 10,000 bp. (B) Precision of genotyping for insertions $\geq$ 10,000 bp.

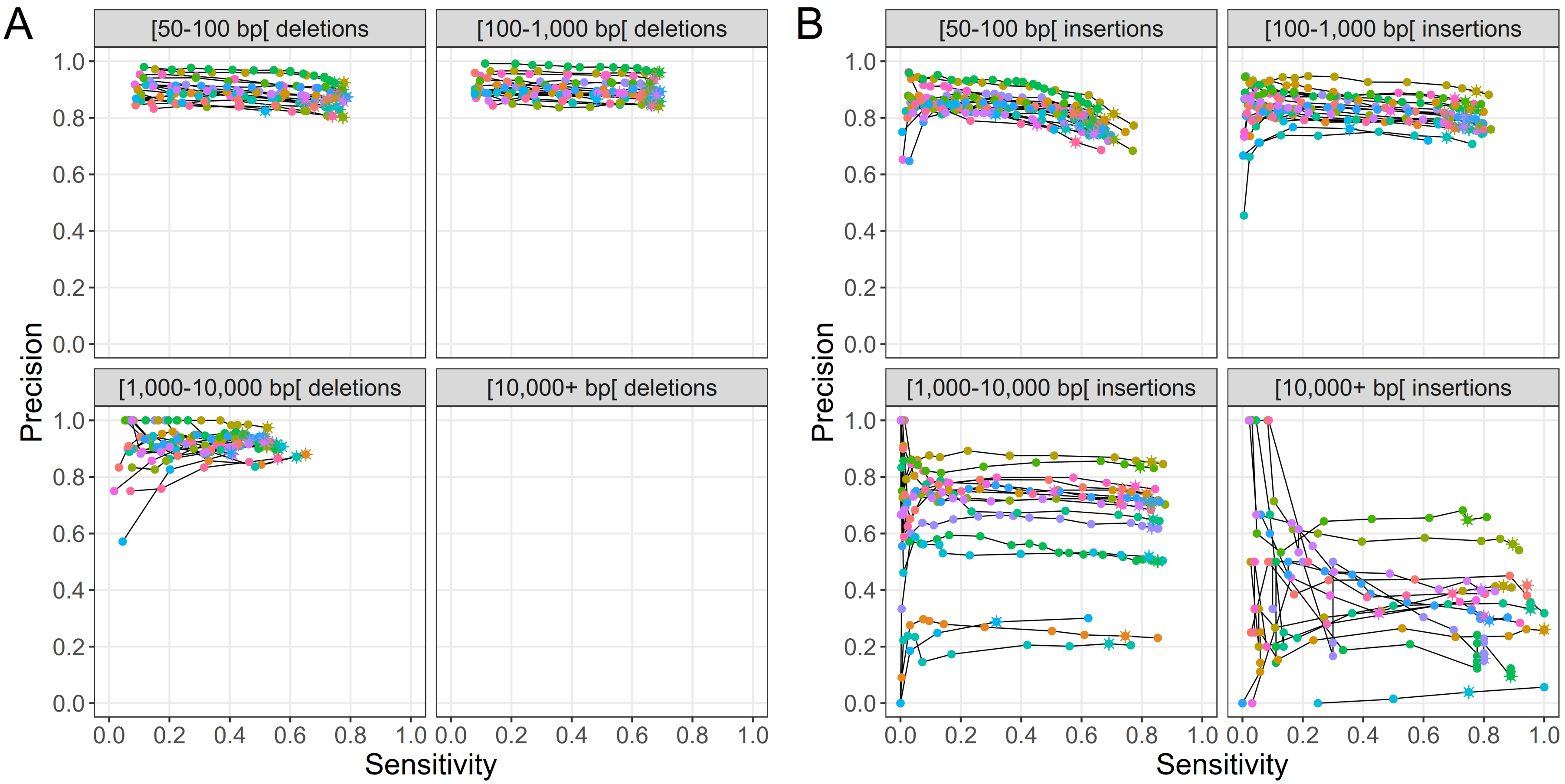

Figure S18: Genotyping sensitivity and precision of (A) deletions and (B) insertions discovered from the Oxford Nanopore data in non-repeat regions. Only SVs with less than 20% overlap to regions annotated as repeats were used for these benchmarks. Each line and color represents one of 17 samples. The points correspond to different filtering thresholds on the minimum number of Illumina reads required to support a genotype call. The asterisks indicate a minimum number of supporting reads of 2; points to the left of these for a given line represent increasingly stringent filtering threshold values. Note that no results were available for deletions larger than 10 kb because these all overlapped repeat regions.

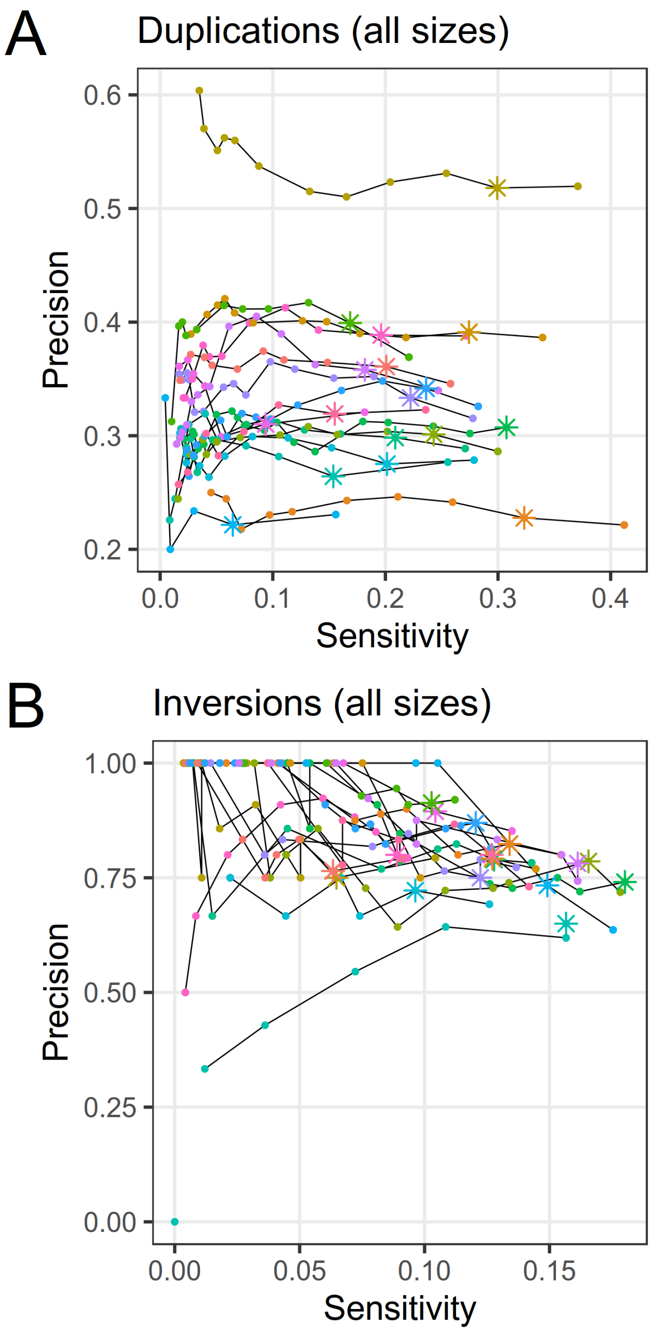

Figure S19: Genotyping sensitivity and precision of (A) duplications and (B) inversions discovered from the Oxford Nanopore data. Each line and color represents one of 17 samples. The points correspond to different filtering thresholds on the minimum number of Illumina reads required to support a genotype call. The asterisks indicate a minimum number of supporting reads of 2; points to the left of these for a given line represent increasingly stringent filtering threshold values.

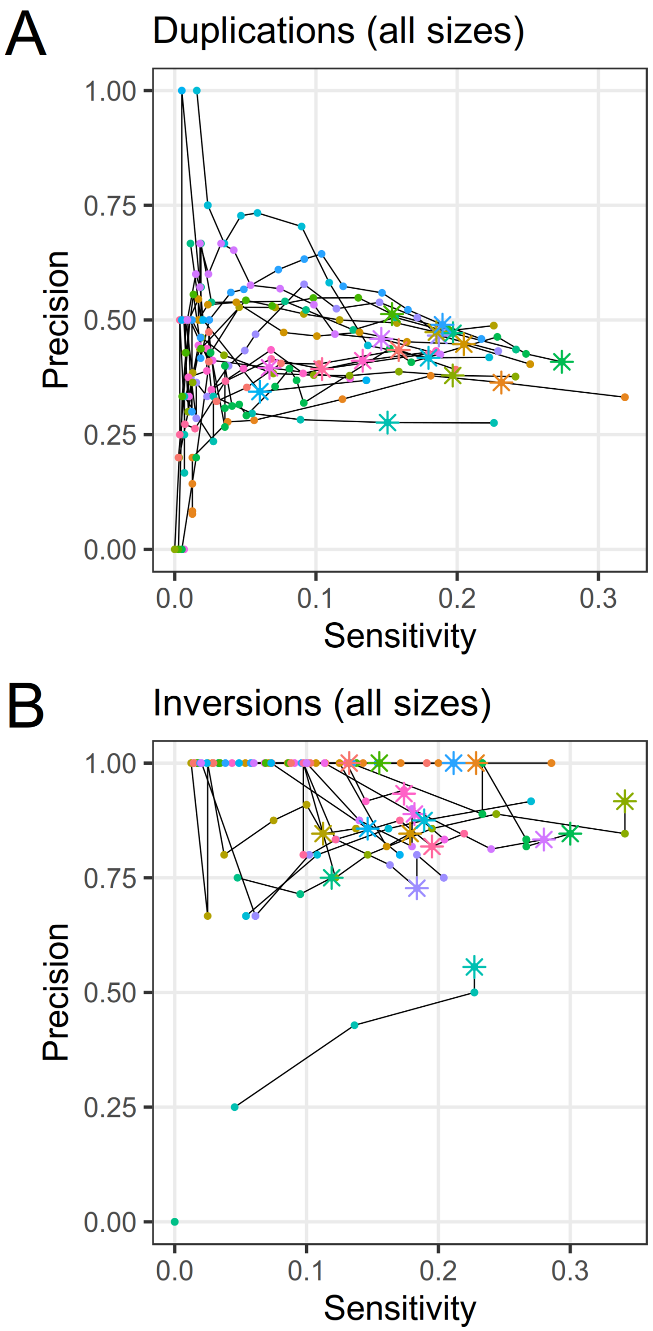

Figure S20: Genotyping sensitivity and precision of (A) duplications and (B) inversions discovered from the Oxford Nanopore data in non-repeat regions. Only SVs with less than 20% overlap to regions annotated as repeats were used for these benchmarks. Each line and color represents one of 17 samples. The points correspond to different filtering thresholds on the minimum number of Illumina reads required to support a genotype call. The asterisks indicate a minimum number of supporting reads of 2; points to the left of these for a given line represent increasingly stringent filtering threshold values.

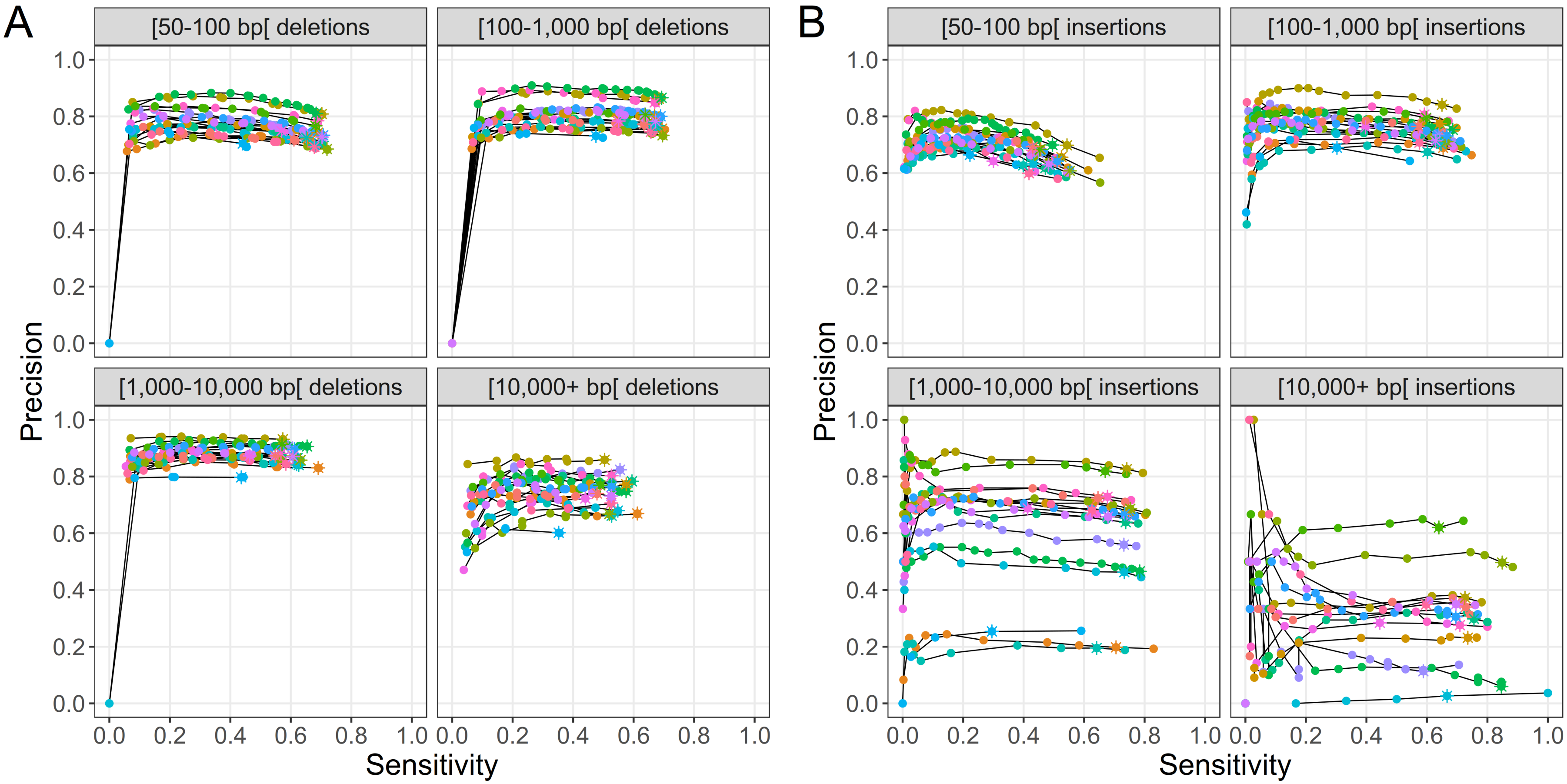

Figure S21: Genotyping sensitivity and precision of (A) deletions and (B) insertions in the merged dataset of SVs discovered from the Illumina and Oxford Nanopore data. Each line and color represents one of 17 samples. The points correspond to different filtering thresholds on the minimum number of Illumina reads required to support a genotype call. The asterisks indicate a minimum number of supporting reads of 2; points to the left of these for a given line represent increasingly stringent filtering threshold values.

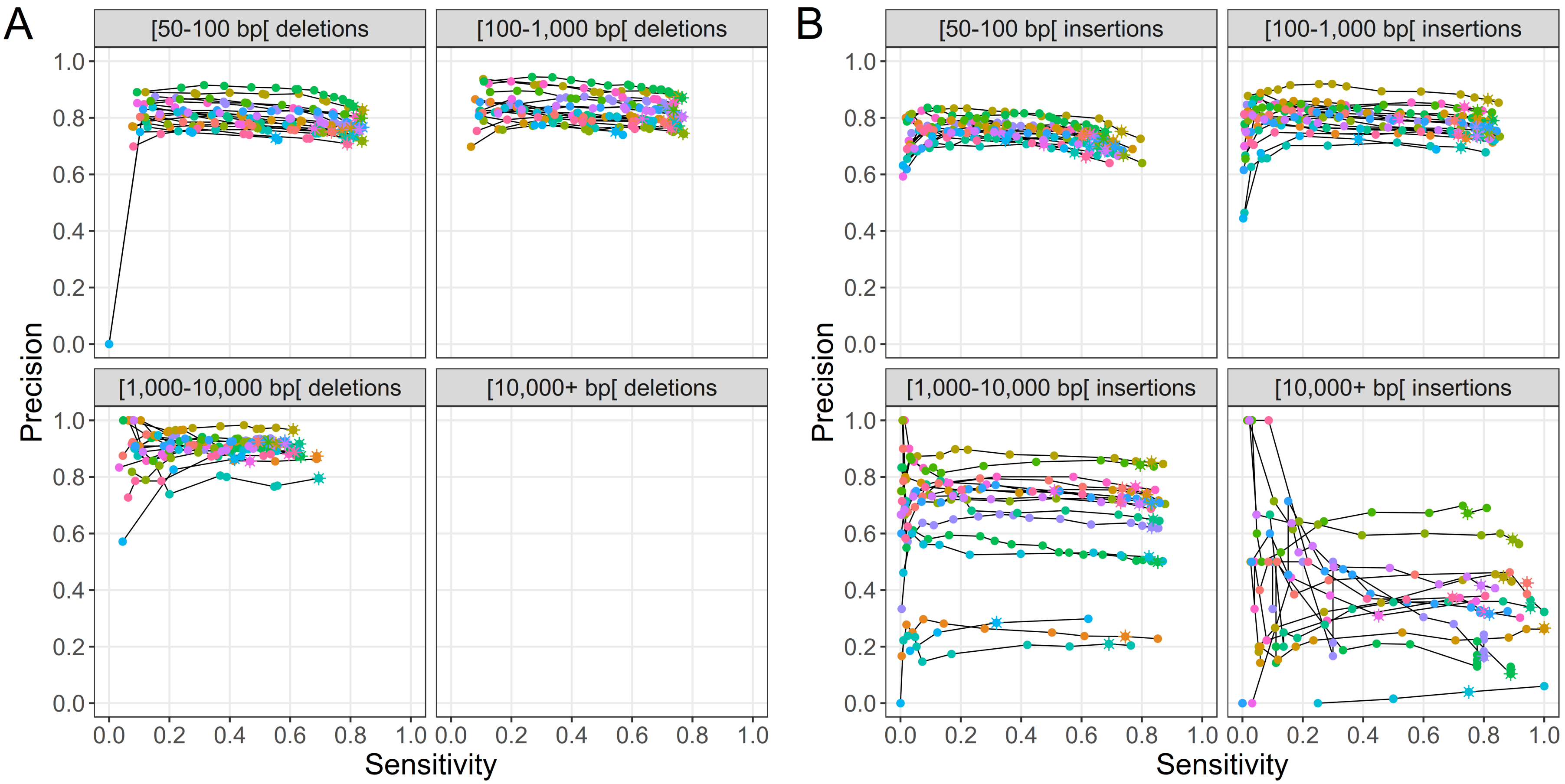

Figure S22: Genotyping sensitivity and precision of (A) deletions and (B) insertions in non-repeat regions in the merged dataset of SVs discovered from the Illumina and Oxford Nanopore data. Only SVs with less than 20% overlap to regions annotated as repeats were used for these benchmarks. Each line and color represents one of 17 samples. The points correspond to different filtering thresholds on the minimum number of Illumina reads required to support a genotype call. The asterisks indicate a minimum number of supporting reads of 2; points to the left of these for a given line represent increasingly stringent filtering threshold values. Note that no results were available for deletions larger than 10 kb because these all overlapped repeat regions.

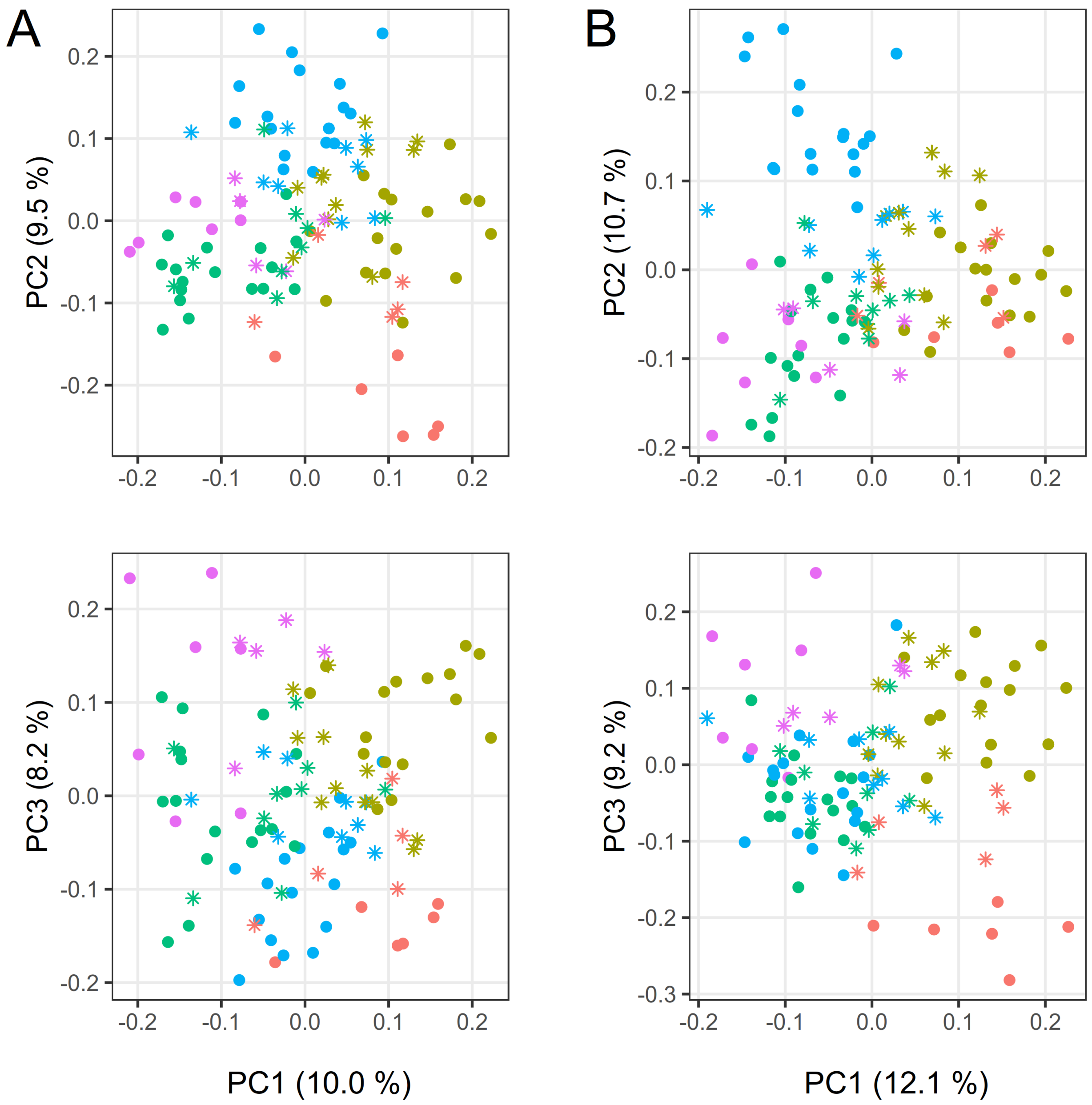

Figure S23: Principal component analyses (PCA) of the whole Canadian soybean panel. PCA were computed based on (A) SVs discovered from Illumina and Oxford Nanopore data, and subsequently genotyped with Illumina data using Paragraph and (B) SNVs called by Platypus from Illumina data. The first three principal components are shown for each PCA. The color of each point indicates the population that the cultivar belongs to according to the fastStructure analysis with SNVs, following the same color scheme as in Figure 4 of the main text. Samples with a maximum q-value below 0.6 (as inferred from fastStructure) are considered admixed and are labeled with asterisks.

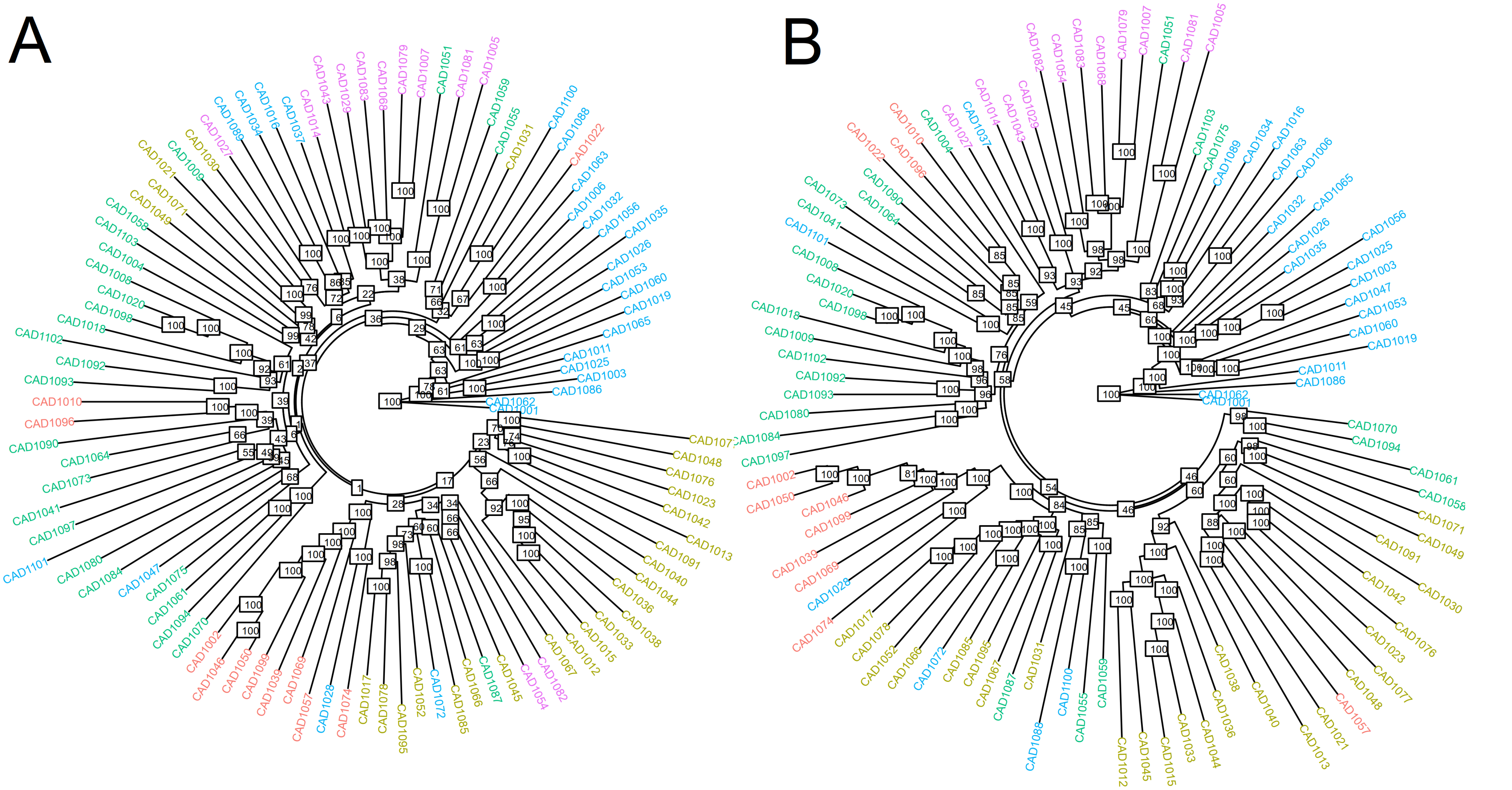

Figure S24: Neighbour-joining phylogenetic trees of the population of 102 Canadian soybean cultivars derived from (A) SVs discovered from Illumina and Oxford Nanopore data, and subsequently genotyped with Illumina data using Paragraph and (B) SNVs called by Platypus from Illumina data. Tip label colors indicate the population that the cultivar belongs to according to the fastStructure analysis with SNVs, following the same color scheme as in Figure 4 of the main text. Node labels indicate the number of trees (out of 100) of the bootstrap analysis that support a given node/grouping.

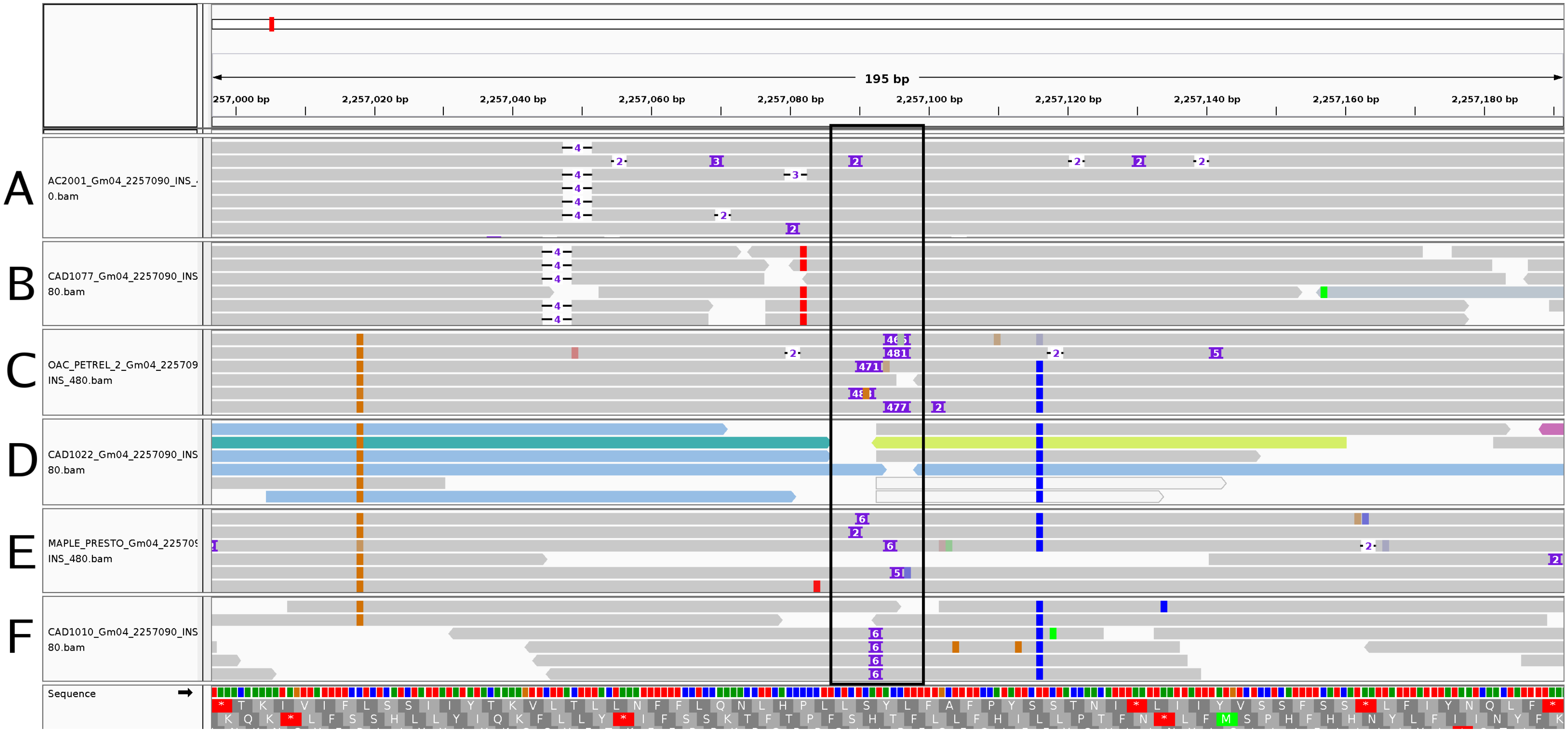

Figure S25: IGV screenshot of Oxford Nanopore and Illumina read alignments of three samples at the location of a polymorphic Stowaway TE insertion at Gm04:2,257,090. (A) Oxford Nanopore and (B) Illumina alignments of sample CAD1077/AC2001, which matches the reference (absence of TE insertion). (C) Oxford Nanopore and (D) Illumina alignments of sample CAD1022/OAC Petrel, which bears the 480-bp Stowaway TE insertion. (E) Oxford Nanopore and (F) Illumina alignments of sample CAD1010/Maple Presto, which bears a 6-bp insertion putatively due to excision of the TE. The black rectangle in the middle of the figure encloses the region where the insertion polymorphisms of interest occur.

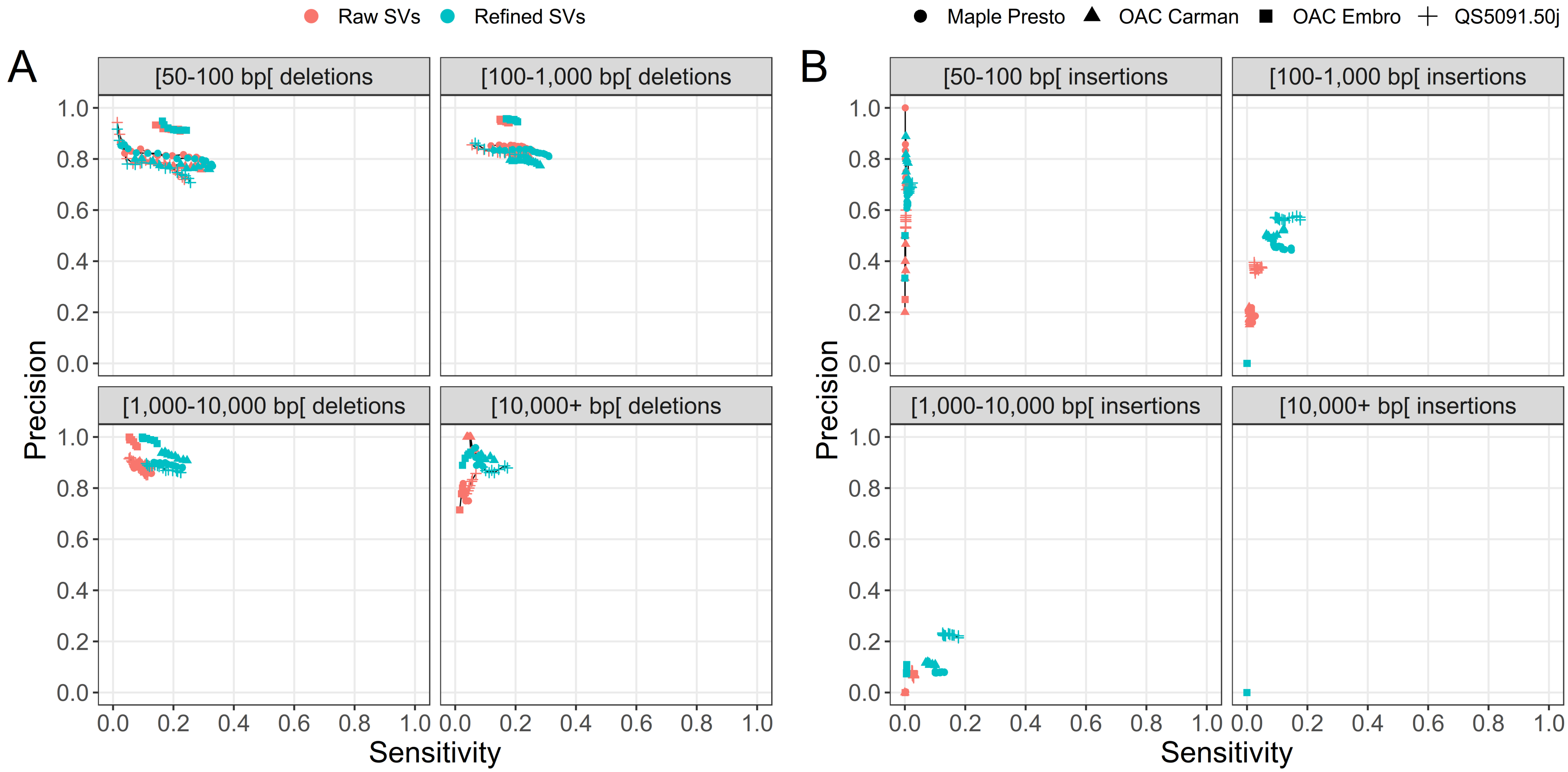

Figure S26: Genotyping sensitivity and precision of raw and refined (A) deletions and (B) insertions discovered by Sniffles on the Oxford Nanopore data for four samples when using BayesTyper as a genotyper. Each line represents data for a single sample whose SV breakpoints have either been refined (refined SVs) or not (raw SVs). The points correspond to different filtering thresholds on the genotype quality (GQ) required to support a genotype call.

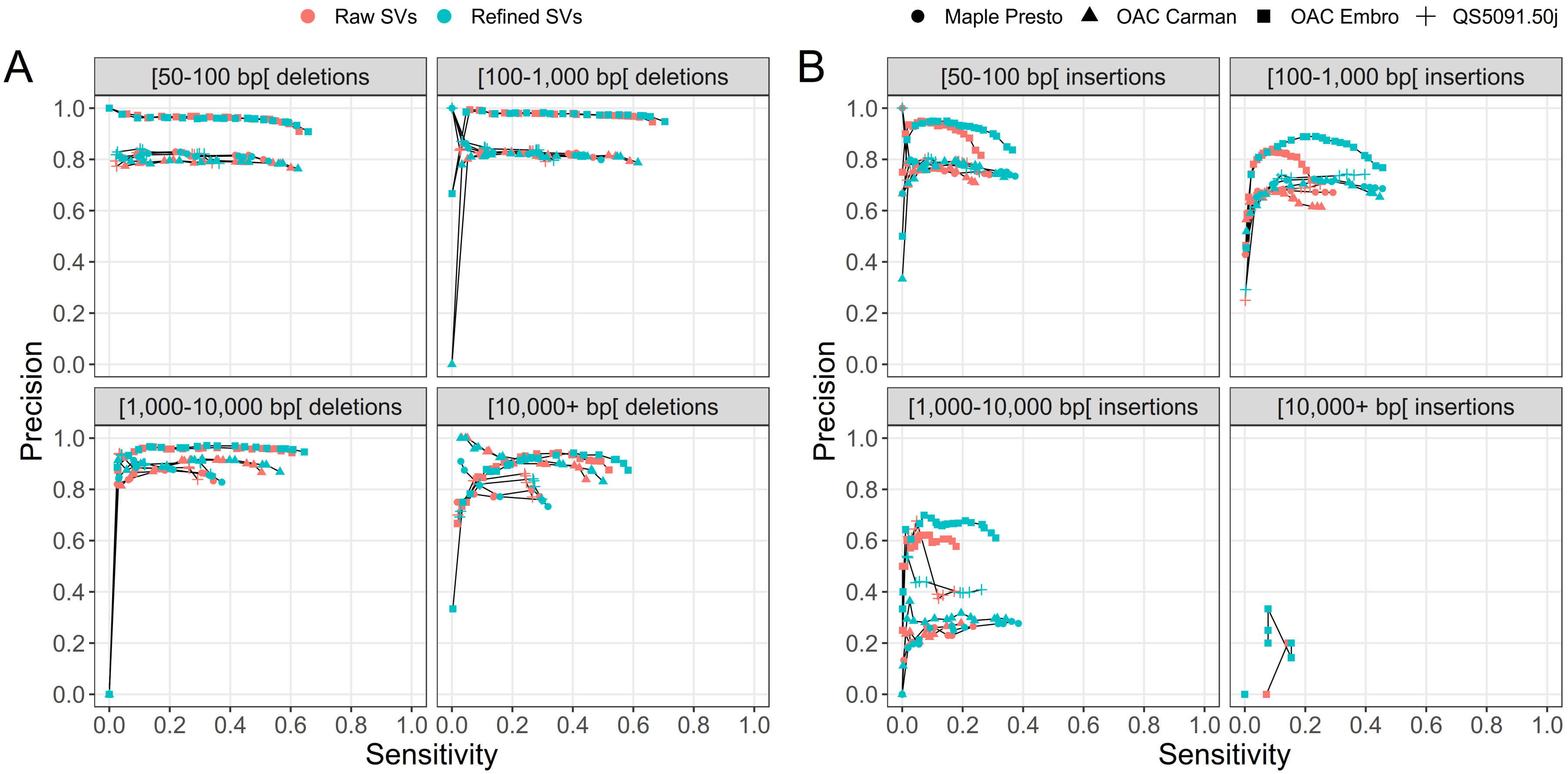

Figure S27: Genotyping sensitivity and precision of raw and refined (A) deletions and (B) insertions discovered by Sniffles on the Oxford Nanopore data for four samples when using vg as a genotyper. Each line represents data for a single sample whose SV breakpoints have either been refined (refined SVs) or not (raw SVs). The points correspond to different filtering thresholds on the genotype quality (GQ) required to support a genotype call.

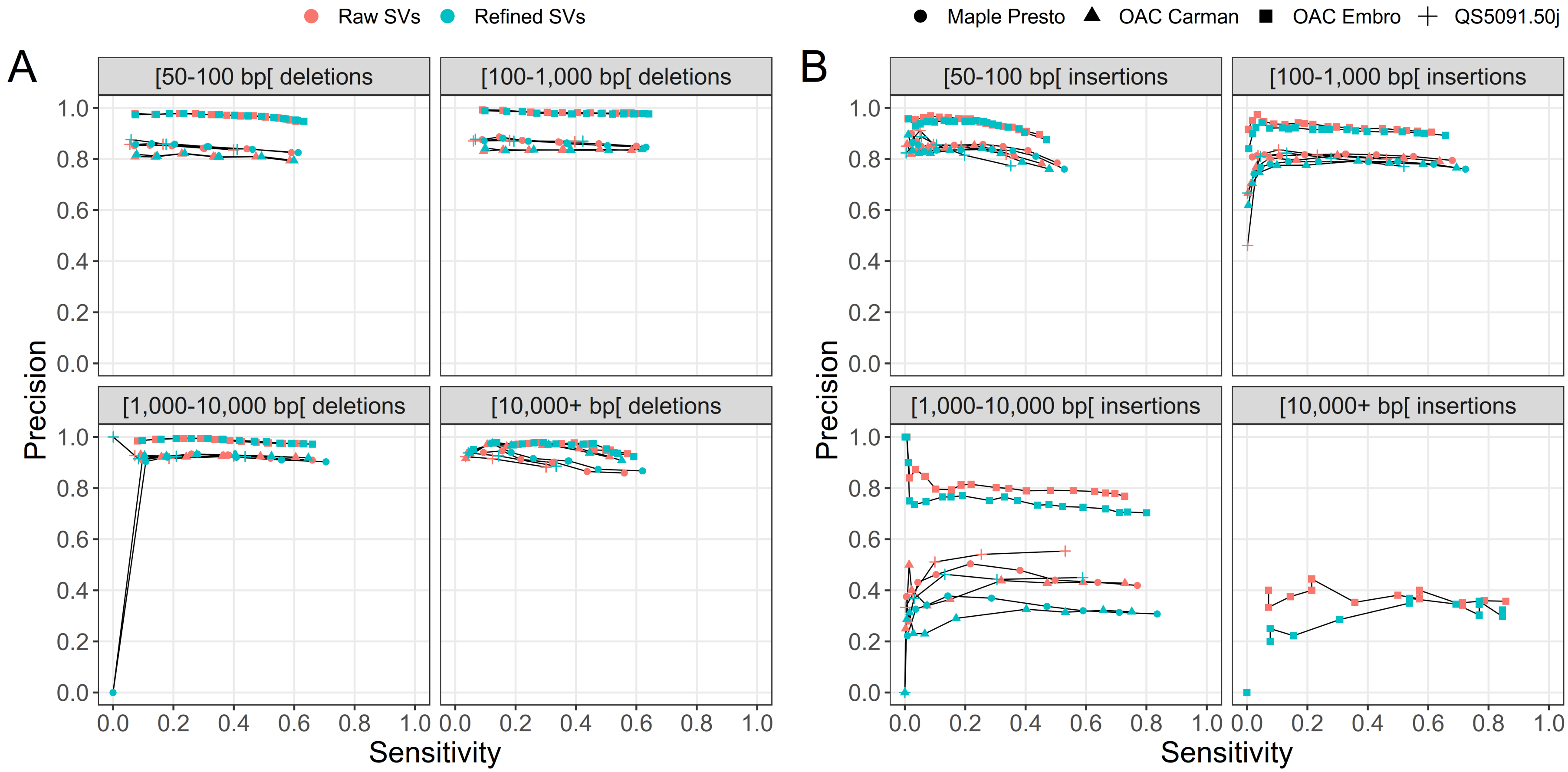

Figure S28: Genotyping sensitivity and precision of raw and refined (A) deletions and (B) insertions discovered by Sniffles on the Oxford Nanopore data for four samples when using Paragraph as a genotyper. Each line represents data for a single sample whose SV breakpoints have either been refined (refined SVs) or not (raw SVs). The points correspond to different filtering thresholds on the minimum number of Illumina reads required to support a genotype call.
